# Sequence analyses of the *distal-less homeobox* gene family in East African cichlid fishes reveal signatures of positive selection

**DOI:** 10.1186/1471-2148-13-153

**Published:** 2013-07-17

**Authors:** Eveline T Diepeveen, Fabienne D Kim, Walter Salzburger

**Affiliations:** 1 Zoological Institute, University of Basel, Vesalgasse 1, 4051 Basel, Switzerland

**Keywords:** *Distal-less homeobox* gene, Molecular evolution, Cichlid fishes, Teleost fishes, Positive selection, Differential selection, Gene duplication, dN/dS

## Abstract

**Background:**

Gen(om)e duplication events are hypothesized as key mechanisms underlying the origin of phenotypic diversity and evolutionary innovation. The diverse and species-rich lineage of teleost fishes is a renowned example of this scenario, because of the fish-specific genome duplication. Gene families, generated by this and other gene duplication events, have been previously found to play a role in the evolution and development of innovations in cichlid fishes - a prime model system to study the genetic basis of rapid speciation, adaptation and evolutionary innovation. The *distal-less homeobox* genes are particularly interesting candidate genes for evolutionary novelties, such as the pharyngeal jaw apparatus and the anal fin egg-spots. Here we study the *dlx* repertoire in 23 East African cichlid fishes to determine the rate of evolution and the signatures of selection pressure.

**Results:**

Four intact *dlx* clusters were retrieved from cichlid draft genomes. Phylogenetic analyses of these eight *dlx* loci in ten teleost species, followed by an in-depth analysis of 23 East African cichlid species, show that there is disparity in the rates of evolution of the *dlx* paralogs. *Dlx3a* and *dlx4b* are the fastest evolving *dlx* genes, while *dlx1a* and *dlx6a* evolved more slowly. Subsequent analyses of the nonsynonymous-synonymous substitution rate ratios indicate that *dlx3b*, *dlx4a* and *dlx5a* evolved under purifying selection, while signs of positive selection were found for *dlx1a*, *dlx2a*, *dlx3a* and *dlx4b*.

**Conclusions:**

Our results indicate that the *dlx* repertoire of teleost fishes and cichlid fishes in particular, is shaped by differential selection pressures and rates of evolution after gene duplication. Although the divergence of the *dlx* paralogs are putative signs of new or altered functions, comparisons with available expression patterns indicate that the three *dlx* loci under strong purifying selection, *dlx3b*, *dlx4a* and *dlx5a*, are transcribed at high levels in the cichlids’ pharyngeal jaw and anal fin. The *dlx* paralogs emerge as excellent candidate genes for the development of evolutionary innovations in cichlids, although further functional analyses are necessary to elucidate their respective contribution.

## Background

Teleost fishes (Teleostei) are among the most diverse lineages on Earth and with nearly 30,000 species the most species-rich vertebrate group. This is in stark contrast to the more basal non-teleost ray-finned fishes that are characterized by small numbers of species. A causal explanation for this discrepancy in speciation rates between the derived Teleostei and the non-teleost ray-finned fishes might be the fish-specific genome duplication (FSGD) that occurred in the ancestor of modern teleosts ([1-4] and references therein). It has been hypothesized that the FSGD has laid down the genetic conditions necessary for the evolution of phenotypic diversity [5], although the exact causes of diversification of such a large clade are likely to be more complex and most probably also include other factors [6].

The Hox gene clusters, which evolved through both tandem and whole genome duplications, represent illustrative examples for the contribution of duplicated genes to morphological evolution across the animal kingdom (see e.g., [7-9]). Together with other homeotic genes, Hox genes play a crucial role in the development of the multicellular body plan (e.g., anterior-posterior patterning; [10]). Furthermore, Hox genes are known to be involved in the development of evolutionary novelties, such as walking limbs and the wings of insects [11-15]. It has been shown that different mechanisms such as *cis*-regulatory evolution, changes in protein function and post-transcriptional regulation of the Hox genes contribute to morphological diversification (reviewed in e.g., [8,15,16]).

East African cichlid fishes show a remarkable level of phenotypic diversity between closely related species and constitute the most diverse adaptive radiations known [17-21]. Although several smaller radiations of cichlid fishes exist outside of Africa (e.g., in Central and South America), an astonishingly high number of cichlid species (close to 1900 species [22]) evolved in and around lakes Malawi, Victoria and Tanganyika in the last few million to several thousand years [23,24]. The various cichlid species differ in body shape, coloration, reproductive biology and mouth morphology [25-27] - traits which are thought to, at least partly, underlie the evolutionary success of cichlid fishes [18,27,28]. Furthermore, several morphological innovations are unique to cichlids or specific lineages thereof. The highly modified and morphological diverse pharyngeal jaw apparatus, for example, correlates with the diversity in foraging strategies exploited by the different cichlid species [27-29]. The occurrence of several color morphs within species, sexual color dimorphism and anal fin egg-spots are three characteristic features of the extremely species-rich and mouthbrooding haplochromine lineage [30].

As a result of their great phenotypic diversity and high number of species, cichlid fishes provide an ideal set up to examine the genetic basis of rapid speciation, evolutionary innovations and adaptation [21,31-37]. An important strategy is the study of so-called candidate genes, i.e., genes with known functions in development in other organisms such as zebrafish. For example, it has been shown that *csf1ra*, which was identified as xanthophore marker in zebrafish [38,39] is involved in the morphogenesis of the egg-spots of haplochromine cichlids [31]. Furthermore, species-specific jaw shapes of different cichlid species correlate with differences in early *bmp4* expression patterns, a gene which has also the potential to change the mandibular morphology in zebrafish [40]. Many of these candidate genes belong to larger gene families such as the endothelin family of ligands and receptors that are putatively involved in the morphogenesis of the pharyngeal jaw apparatus and pigmentation [36], and the above mentioned Hox gene clusters [41].

Recently, Renz *et al. *[35] characterized seven *distal-less homeobox* (*dlx*) genes and examined their expression patterns in the developing pharyngeal arches and/or pharyngeal teeth of the haplochromine cichlid *Astatotilapia burtoni*. The vertebrate *dlx* genes are widely known for their crucial roles in the development (of components) of the nervous system, craniofacial skeleton and connective tissue and in the formation of appendages [reviewed in 42]. These functions seem to be conserved across a wide range of animal taxa. For example, the vertebrate *dlx* genes are homologs of, and share several functions with, the single *Distal-less* (*dll*) gene of *Drosophila *[42]. Within vertebrates, the expression patterns of dlx homologs is similar in early development [35,42-45]. At the same time, *dlx* genes have been implicated with evolutionary novelties such as the eyespots in various butterfly species [46-48], the insect antenna [49,50] and the vertebrate craniofacial bones [51].

Phylogenetic analyses and the chromosomal arrangements of the vertebrate *dlx* genes suggest that the extant *dlx* repertoire has evolved by an initial tandem duplication, followed by two rounds of whole genome duplication in the lineage towards vertebrates and a third one in the lineage towards teleost fishes, the FSGD [35,44,45,52]. These duplication events resulted in multiple so-called *dlx* clusters, in which two *dlx* genes are located in a tail-to-tail arrangement on the respective chromosome. Linked *dlx* genes are transcribed coincidently due to shared *cis*-regulating elements in the intergenic regions [35,42,43]. Four of these *dlx* clusters have been identified in teleost fish; *dlx1a*-*dlx2a*, *dlx3a*-*dlx4a*, *dlx3b*-*dlx4b* and *dlx5a*-*dlx6a *[43,44]. Seven of these *dlx* genes have been identified in the cichlid *A*. *burtoni*, where they are expressed in tissues that make up putative evolutionary innovations [35].

Here, we analyzed the *dlx* repertoire and diversity in detail in a phylogenetically representative set of 23 East African cichlid species in order to study the molecular evolution of this prominent developmental gene family. To this end, we first performed phylogenetic comparisons of the dlx proteins including the sixty amino acids long homeobox domain in a range of teleost fishes in combination with blast searches of these sequences against the draft genomes of four cichlid species. Teleost and cichlid-specific phylogenies were examined to compare the rate of evolution of both between and within *dlx* gene trees. Several studies have shown that loci putatively involved in evolutionary innovations are characterized by adaptive protein evolution in cichlids [31,36,53]. Therefore, all loci were screened for elevated rates of protein evolution by means of *d*_N_/*d*_S_ analyses. Our analyses indicate the presence of *dlx3a* in cichlids and that the *dlx* repertoire of cichlid fishes is shaped by differential selection pressures and rates of evolution, with signs of positive selection on specific sites in *dlx1a*, *dlx2a*, *dlx3a* and *dlx4b*.

## Methods

### Dlx protein sequence comparison in teleost fishes

The sequences of nine dlx proteins (i.e., dlx1a, dlx2a, dlx2b, dlx3a, dlx3b, dlx4a, dlx4b, dlx5a and dlx6a) of seven teleost species (i.e., zebrafish (*Danio rerio*), Atlantic cod (*Gradus morhua*), three-spined stickleback (*Gasterosteus aculeatus*), spotted green pufferfish (*Tetraodon nigroviridis*), Japanese pufferfish (*Takifugu rubripes*), Japanese medaka (*Oryzias latipes*) and Nile Tilapia (*Oreochromis niloticus*)) were obtained from Ensemble (release 68, July 2012; see Additional file 1 for accession numbers). *Dlx2b* was excluded from all further analyses, due to its lineage-specific loss in percomorphs, to which all studied species belong except *D. rerio* and *G. morhua* (see [35]). Sequences were aligned with Tcoffee [54,55], ambiguous sites were removed and tblastx searches were performed to determine dlx protein sequences in the draft cichlid genomes of *Astatotilapia burtoni*, *Neolamprologus brichardi* and *Pundamilia nyererei* (BROAD Institute, unpublished data; see Additional file 1 for scaffold numbers). To determine the rate of evolution for each of the dlx proteins, phylogenetic analyses were performed in PAUP* 4.0 [56] under parsimony settings and the number of amino acid changes was obtained. *D. rerio* or *G. morhua* was used as outgroup species and bootstrap analyses with 100 replicates were conducted to test the robustness of the obtained topologies. Next, the sixty amino acids long homeobox domain was extracted from the sequences and aligned to the homeobox domain of the single *Distal-less* (*Dll*) gene of *Drosophila melanogaster* [Ensemble: FBgn0000157] in Geneious 5.6 [57] for closer inspection of the conservation of the domain and to identify gene-specific substitutions.

### Cichlid samples and genomic DNA sequencing

White muscle and/or fin clip samples were collected during fieldwork in Zambia in 2007 and 2008 using a standard operating procedure described in [29]. In total 23 Lake Tanganyikan cichlid species were included in this study (Additional file 2). Genomic DNA was extracted following a standard Proteinase K protocol [58]. Cichlid-specific PCR primers were designed based on available and/or draft genomic and transcriptomic cichlid sequences, which were identified by tblastx searches of publicly available *dlx* sequences from other teleost species (see Additional file 1 for species and accession numbers). This was done for eight *dlx* loci: *dlx1a*, *dlx2a*, *dlx3a*, *dlx3b*, *dlx4a*, *dlx4b*, *dlx5a* and *dlx6a* (see Additional file 3 for primer sequences). Standard PCR reactions, purification steps and sequencing reactions were set up and performed as described elsewhere [36]. PCR products of the partially sequenced loci were visualized with GelRed (Biotium) on a 1.5% agarose gel and sequenced on a 3130*xl* capillary sequencer (Applied Biosystems). Partial sequences were aligned and visually inspected using Codon Code Aligner 3.7.1 (CodonCode Corporation, Dedham, MA). Exon/intron boundaries were determined by homology comparisons with the sequences from the other teleost species. All generated cichlid *dlx* sequences have been deposited into GenBank [GenBank: KC285366-KC285546] (Additional file 2).

### Phylogenetic analyses of cichlid samples

Individual gene trees were constructed using maximum likelihood in PAUP* 4.0 [56] and Bayesian Inference in MrBayes 3.2 [59,60]. The best-fitting model of nucleotide substitution was determined with the corrected Akaike information criteria and likelihood ratio tests conducted in jModeltest 0.1.1 [61,62]. Bootstrap analyses with 100 replicates were performed in PAUP* and MrBayes was run for 10.500.000 generations. *Oreochromis tanganicae* was used as outgroup (see e.g., [63]). Phylogenetic analysis of a concatenated dataset of 9.2 kb was performed as described above in PAUP* to generate a common input tree file (treeBASE submission 14433) for the subsequent analyses.

Coding sequence data of the 23 cichlid species (treeBASE submission 14433) was assessed with both site- and branch-site models as implemented in the program Codeml of the software package PAML (Phylogenetic Analysis by Maximum Likelihood) 4.3 [64,65]. The following parameters were estimated for all eight *dlx* datasets under different models: the nonsynonymous/synonymous substitution rate ratio, *ω*, the proportion of sites assigned to an *ω* category, *p*_*0,1,2*_, and the *p* and *q* parameters of the *β* distribution. Tests of positively selected sites were conducted by performing Likelihood Ratio Tests (LRT) of the following model comparisons: M1a (Nearly Neutral) with M2a (Positive Selection), M7 (β) with M8 (β & ω_s_ ≥ 1), and M8a (β & ω_s_ = 1) with M8. The comparison between M0 (one-ratio) and M3 (discrete) was used as a test of variable *ω* among sites. The naïve empirical Bayes (NEB; [66,67]) and the Bayes empirical Bayes (BEB; [68]) criteria were used to calculate the posterior probabilities for site classes and the BEB was used to identify sites under positive selection when the LRT was significant. To test whether the *dlx* genes evolved under non-neutral evolution in specific lineages a LRT between the null model (ω_s_ = 1) and the alternative model (ω_s_ ≥ 1) was performed in the branch-site analyses. Branches of interest, or so-called foreground branches, were chosen based on the results of the phylogenetic analyses and branch tests performed in Hyphy ([69], following [36]).

Additional tests of positive selection on the partial *dlx* sequences were performed with the Sitewise Likelihood Ratio estimation of selection program (SLR; [70]) v1.3. The common input tree file was used (see above) and the significance level was set to 95%.

Amino acid substitutions were screened for possible effect on protein function with the program SIFT (Sorting Intolerant from Tolerant; [71].

## Results

### Dlx protein sequence comparison in teleost fishes

**Figure 1:**
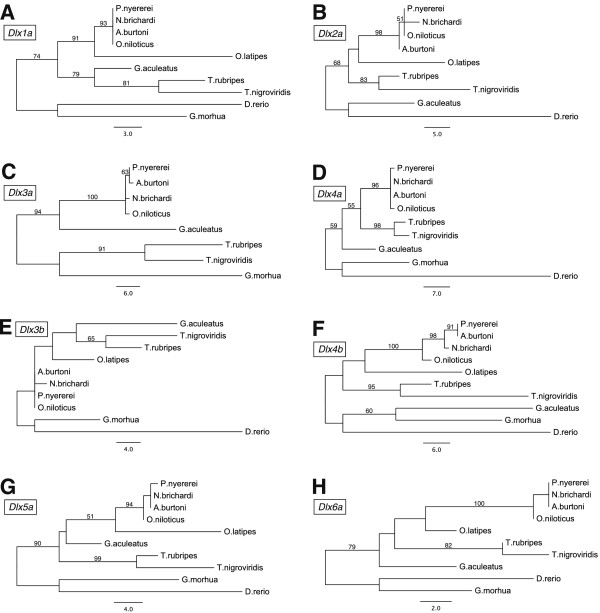
**Maximum Likelihood phylogenetic hypotheses for the eight dlx paralogs in teleost fishes. (A)** Dlx1a (254 amino acids (aa)). **(B)** Dlx2a (276 aa). **(C)** Dlx3a (307 aa). **(D)** Dlx4a (259 aa). **(E)** Dlx3b (283 aa). **(F)** Dlx4b (257 aa). **(G)** Dlx5a (285 aa). **(H)** Dlx6a (247 aa). Bootstrap probabilities (PAUP*) above 50% are shown.

The tblastx searches of the teleost dlx proteins resulted in the retrieval of eight *dlx* genes in all four cichlid species. Furthermore, the genomic locations of these *dlx* loci (Additional file 1) indicate that four *dlx* clusters are present in the cichlid lineage: *dlx1a*-*dlx2a*; *dlx3a*-*dlx4a*; *dlx3b*-*dlx4b* and *dlx5a*-*dlx6a*. All other teleost species examined contain this full set of genes, except zebrafish, in which *dlx3a* could not be located, and medaka, in which *dlx4b* is missing, as previously noted [35,44,45]. Interestingly, in contrast to Renz *et al.*[35] we do find evidence for the existence of *dlx3a* in cichlids, including *A. burtoni* (Figure 1, Additional file 4).

The sixty amino acid long homeobox domain of the eight teleost dlx proteins are highly conserved among teleost fish and even between teleosts and the single Dll protein of *D. melanogaster* (Additional file 4). Despite the high level of conservation, several locus-specific amino acid substitutions are present in the paralogs, making it possible to distinguish between individual dlx homeobox domains.

Phylogenetic analyses of the dlx protein sequences were performed to examine the rate of evolution of the dlx paralogs in teleost fishes. The overall and relative longest trees were found for *dlx4b* and *dlx3a*, while for *dlx1a* and *dlx6a* the shortest tree lengths were observed (Figure 1 and Table 1). Typically the longest branches were observed in the two basal species *D. rerio* and *G. morhua*. Interestingly, relatively long branch lengths for the branch towards the four cichlid species were observed for *dlx3a* and *dlx6a*, indicting elevated rates of molecular evolution. The opposite scenario was observed in the overall more conserved dlx1a and dlx5a proteins. To study these effects in more detail cichlid specific gene trees were constructed.

**Table 1:** Overall and relative tree lengths of teleost protein phylogenies

**dlx paralog**	**Length (# amino acids)**	**Tree length (absolute)**	**Tree length (relative)**
dlx1a	254	93	0.366
dlx2a	276	122	0.442
dlx3a	307	179	0.583
dlx3b	283	108	0.382
dlx4a	259	123	0.475
dlx4b	257	222	0.864
dlx5a	285	125	0.439
dlx6a	247	56	0.227

### The rate of *dlx* gene evolution in East African cichlid fishes

To reconstruct the molecular evolutionary history of the *dlx* homologs in East African cichlid species, we determined the rate of evolution and the signatures of selection pressure in a phylogenetically representative set of 23 species. The gene trees of the obtained partial cichlid *dlx* sequences resulted in various polytomies (Additional file 5), probably due to the limited size of some of the datasets (minimum of 0.7 kb). Although for each gene tree specific branches were observed with relative long branches, there is not a particular species or clade that has evolved under faster rates of evolution in all of the *dlx* loci examined. Interestingly, three branches have relative long branch lengths in multiple topologies: the branch towards the Lamprologini (*dlx2a*, *dlx4a* and *dlx5a*), *C. leptosoma* (*dlx3b*, *dlx4a* and *dlx5a*) and *C. furcifer* (*dlx1a* and *dlx6a*). The relative tree lengths (Additional file 5 and Table 2) of these gene trees reveal similar results as the teleost protein trees, with *dlx4b* and *dlx3a* evolving fastest and *dlx1a* and *dlx6a* evolving more slowly.

**Table 2:** **Overall and relative tree lengths of cichlid *dlx *gene trees**

**dlx paralog**	**Length (# base pairs)**	**Tree length (absolute)**	**Tree length (relative) 10**^**-4**^
dlx1a	737	0.036	0.483
dlx2a	1371	0.094	0.684
dlx3a	666	0.061	0.910
dlx3b	1972	0.120	0.609
dlx4a	1166	0.104	0.892
dlx4b	722	0.068	0.937
dlx5a	1538	0.093	0.607
dlx6a	1710	0.093	0.542

### Observed signatures of selection pressure in cichlid *dlx* loci

To investigate signatures of selection pressure in the *dlx* loci, we performed detailed analyses of the *d*_N_/*d*_S_ ratios. Maximum likelihood parameter estimations for *ω*, *p*_*0,1,2*_ and *p* and *q* under different evolutionary models can be found in Table 3 for all eight *dlx* loci. Estimations of *ω* under the M0 model suggest that the *dlx* genes evolved under purifying selection with *ω* ranging from 0.0001 (*dlx5a*) to 0.457 (*dlx2a*). A small proportion of sites, 0.00001-24.2%, was estimated to have evolved neutrally (*ω* = 1) under the M1a model. By using models that allow *ω* to vary among sites, 0.7-12.3% of sites was detected with *ω* > 1 in *dlx1a*, *dlx2a*, *dlx3a*, *dlx4b* and *dlx6a*. Overall, most sites are estimated to have evolved under purifying selection, with highest proportions found in *dlx3b*, *dlx4a* and *dlx5a*.

**Table 3:** **Site model parameter estimates generated by the CodeML analyses for the eight *dlx *paralogs**

**Locus**	**Parameter estimates under different models**						
	**M0 (one ratio)**	**M1a (neutral)**	**M2a (selection)**	**M3 (discrete)**	**M7 (ß)**	**M8 (ß & ω)**	**M8a (ß & ω**_**s**_ **= 1)**
*dlx1a*	*ω* = 0.111	*p*_*0*_ = 0.940, *ω*_*0*_ = 0	*p*_*0*_ = 0.980, *ω*_*0*_ = 0	*p*_*0*_ = 0.640, *ω*_*0*_ = 0	*p* = 0.005, *q* = 0.049	*p* = 0.005, *q* = 22.651	*p* = 1.333, *q* = 24.671
		*p*_*1*_ = 0.060, *ω*_*1*_ = 1	*p*_*1*_ = 0, *ω*_*1*_ = 1	*p*_*1*_ = 0.340, *ω*_*1*_ = 0		*p*_*0*_ = 0.980	*p*_*0*_ = 0.936
			***p***_***2***_ **= 0.020, *ω***_***2***_ **= 12.580**	***p***_***2***_ **= 0.020, *ω***_***2***_ **= 12.580**		***p***_***1***_ **= 0.020, *ω*** **= 12.580**	*p*_*1*_ = 0.065, *ω* = 1
*dlx2a*	*ω* = 0.457	*p*_*0*_ = 0.833, *ω*_*0*_ = 0	*p*_*0*_ = 0.879, *ω*_*0*_ = 0	*p*_*0*_ = 0, *ω*_*0*_ = 0	*p* = 0.005, *q* = 0.011	*p* = 0.005, *q* = 0.046	*p* = 0.005, *q* = 12.618
		*p*_*1*_ = 0.167, *ω*_*1*_ = 1	*p*_*1*_ = 0.110, *ω*_*1*_ = 1	*p*_*1*_ = 0.986, *ω*_*1*_ = 0.081		*p*_*0*_ = 0.989	*p*_*0*_ = 0.833
			***p***_***2***_ **= 0.010, *ω***_***2***_ **= 18.903**	***p***_***2***_ **= 0.014, *ω***_***2***_ **= 16.629**		***p***_***1***_ **= 0.011, *ω*** **= 18.053**	*p*_*1*_ = 0.167, *ω* = 1
*dlx3a*	*ω* = 0.320	*p*_*0*_ = 0.758, *ω*_*0*_ = 0	*p*_*0*_ = 0.921, *ω*_*0*_ = 0	*p*_*0*_ = 0.877, *ω*_*0*_ = 0	*p* = 0.005, *q* = 0.017	*p* = 0.015, *q* = 0.088	*p* = 0.005, *q* = 13.826
		*p*_*1*_ = 0.242, *ω*_*1*_ = 1	*p*_*1*_ = 0.034, *ω*_*1*_ = 1	***p***_***1***_ **= 0.120, *ω***_***1***_ **= 2.099**		*p*_*0*_ = 0.032	*p*_*0*_ = 0.758
			***p***_***2***_ **= 0.045, *ω***_***2***_ **= 4.451**	***p***_***2***_ **= 0.003, *ω***_***2***_ **= 11.657**		***p***_***1***_ **= 0.032, *ω*** **= 5.096**	*p*_*1*_ = 0.242, *ω* = 1
*dlx3b*	*ω* = 0.047	*p*_*0*_ = 1, *ω*_*0*_ = 0.047	*p*_*0*_ = 1, *ω*_*0*_ = 0.047	*p*_*0*_ = 0.243, *ω*_*0*_ = 0.047	*p* = 4.896, *q* = 99.00	*p* = 4.895, *q* = 99.00	*p* = 4.895, *q* = 99.00
		*p*_*1*_ = 1E-5, *ω*_*1*_ = 1	*p*_*1*_ = 0, *ω*_*1*_ = 1	*p*_*1*_ = 0.262, *ω*_*1*_ = 0.047		*p*_*0*_ = 1	*p*_*0*_ = 1
			*p*_*2*_ = 0, *ω*_*2*_ = 1	*p*_*2*_ = 0.495, *ω*_*2*_ = 0.047		*p*_*1*_ = 1E-5, *ω* = 1	*p*_*1*_ = 1E-5, *ω* = 1
*dlx4a*	*ω* = 0.050	*p*_*0*_ = 1, *ω*_*0*_ = 0.050	*p*_*0*_ = 1, *ω*_*0*_ = 0.050	*p*_*0*_ = 0.216, *ω*_*0*_ = 0.050	*p* = 5.267, *q* = 99.00	*p* = 5.266, *q* = 99.00	*p* = 0.137, *q* = 2.305
		*p*_*1*_ = 1E-5, *ω*_*1*_ = 1	*p*_*1*_ = 0, *ω*_*1*_ = 1	*p*_*1*_ = 0.384, *ω*_*1*_ = 0.050		*p*_*0*_ = 1	*p*_*0*_ = 1
			*p*_*2*_ = 0, *ω*_*2*_ = 1	*p*_*2*_ = 0.400, *ω*_*2*_ = 0.050		*p*_*1*_ = 1E-5, *ω* = 1	*p*_*1*_ = 1E-5, *ω* = 1
*dlx4b*	*ω* = 0.259	*p*_*0*_ = 0.872, *ω*_*0*_ = 0	*p*_*0*_ = 0.993, *ω*_*0*_ = 0.090	*p*_*0*_ = 0, *ω*_*0*_ = 0	*p* = 0.005, *q* = 0.042	*p* = 9.871, *q* = 99.00	*p* = 0.005, *q* = 30.817
		*p*_*1*_ = 0.128, *ω*_*1*_ = 1	*p*_*1*_ = 0, *ω*_*1*_ = 1	*p*_*1*_ = 0.993, *ω*_*1*_ = 0.090		*p*_*0*_ = 0.993	*p*_*0*_ = 0.872
			***p***_***2***_ **= 0.007, *ω***_***2***_ **= 12.858**	***p***_***2***_ **= 0.007, *ω***_***2***_ **= 12.858**		***p***_***1***_ **= 0.007, *ω*** **= 12.859**	*p*_*1*_ = 0.128, *ω* = 1
*dlx5a*	*ω* = 1E-4	*p*_*0*_ = 1, *ω*_*0*_ = 0	*p*_*0*_ = 1, *ω*_*0*_ = 0	*p*_*0*_ = 0.424, *ω*_*0*_ = 0	*p* = 0.005, *q* = 1.294	*p* = 0.005, *q* = 99.00	*p* = 0.005, *q* = 44.274
		*p*_*1*_ = 1E-5, *ω*_*1*_ = 1	*p*_*1*_ = 0, *ω*_*1*_ = 1	*p*_*1*_ = 0.420, *ω*_*1*_ = 0		*p*_*0*_ = 1	*p*_*0*_ = 1
			*p*_*2*_ = 0, *ω*_*2*_ = 1	*p*_*2*_ = 0.156, *ω*_*2*_ = 0		*p*_*1*_ = 1E-5, *ω* = 1	*p*_*1*_ = 1E-5, *ω* = 1
*dlx6a*	*ω* = 0.029	*p*_*0*_ = 0.967, *ω*_*0*_ = 0	*p*_*0*_ = 0.992, *ω*_*0*_ = 0	*p*_*0*_ = 0.899, *ω*_*0*_ = 0	*p* = 0.066, *q* = 1.696	*p* = 0.005, *q* = 2.390	*p* = 0.005, *q* = 1.672
		*p*_*1*_ = 0.033, *ω*_*1*_ = 1	*p*_*1*_ = 0, *ω*_*1*_ = 1	*p*_*1*_ = 0.093, *ω*_*1*_ = 0		*p*_*0*_ = 0.992	*p*_*0*_ = 0.967
			***p***_***2***_ **= 0.008, *ω***_***2***_ **= 6.336**	***p***_***2***_ **= 0.008, *ω***_***2***_ **= 6.336**		***p***_***1***_ **= 0.008, *ω*** **= 6.336**	*p*_*1*_ = 0.033, *ω* = 1

*Note:* p_0-2_ are the proportions of sites assigned to an *ω* category or to a beta distribution with p and q as parameters. *ω* ratios greater than one and their corresponding proportions are depicted in bold.

**Figure 2:**
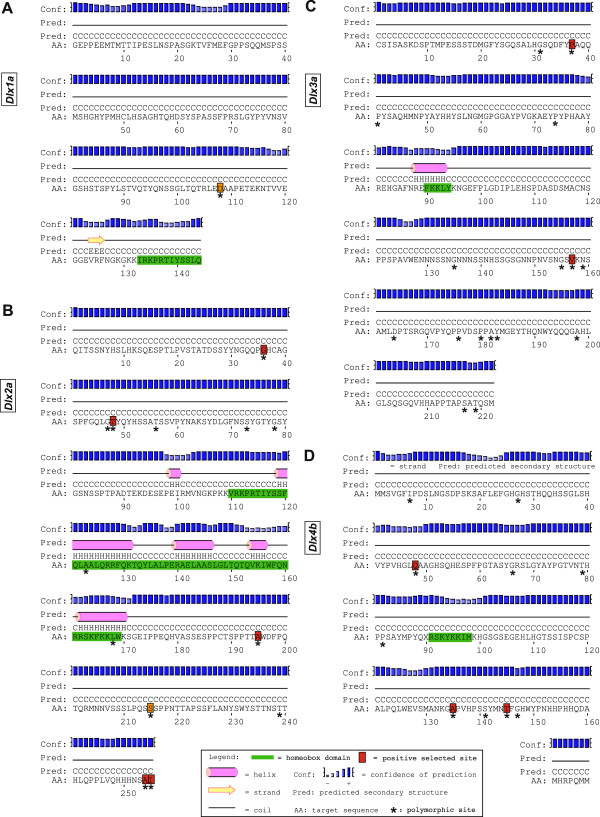
**Secondary structure and positively selected sites for four partially sequenced *Astatotilapia burtoni *Dlx proteins.** Secondary structure predictions were obtained from the PSIPRED server (http://bioinf.cs.ucl.ac.uk/psipred/). Positively selected sites identified by the site model analyses (CodeML) and the SLR analyses are highlighted in red (BEB and/or SLR) or orange (NEB) boxes. **(A)** Dlx1a. **(B)** Dlx2a. **(C)** Dlx3a. **(D)** Dlx4b.

Likelihood ratio tests of the subsequent model comparisons (Table 4) resulted in the rejection of the null models in only the following comparisons per loci: *dlx1a* (M8a-M8), *dlx2a* (all four comparisons), *dlx3a* (M0-M3; M8a-M8) and *dlx4b* (all four comparisons). Positively selected sites were detected with the BEB in *dlx2a* (5 sites), *dlx3a* (1 site) and *dlx4b* (3 sites; see Table 4, Figure 2). The less constraining analyses with the NEB resulted in two more putative positively selected sites in *dlx1a* (1) and *dlx2a* (1; Figure 2). Fewer positively selected sites were identified by the SLR analyses for *dlx2a* (position: 36; significance: 99%), *dlx3a* (37, 157; 99%, 95%) and *dlx4b* (145; 99%).

**Table 4:** **Likelihood ratio test (LTR) statistics of site model comparisons for *dlx1a*, *dlx2a*, *dlx3a *and *dlx4b***

**Locus**	**Test**	**LRT (2∆ *l *)**	**p**	**Selected sites (BEB)**
*dlx1a*	M0 vs M3	8.416	0.077	-
	M1a vs M2a	3.396	ns	-
	M7 vs M8	3.680	ns	-
	M8a vs M8	5.084	0.012	-
*dlx2a*	M0 vs M3	70.438	<0.001	-
	M1a vs M2a	39.198	<0.001	**36A**, **48 T**, *255I*
	M7 vs M8	43.093	<0.001	**36A**, **48 T**, *195A*, *254A*, *255I*
	M8a vs M8	39.168	<0.001	See M7 vs M8 comparison
*dlx3a*	M0 vs M3	12.605	0.013	**-**
	M1a vs M2a	3.858	ns	**-**
	M7 vs M8	4.258	ns	**-**
	M8a vs M8	3.872	0.025	*37S*
*dlx4b*	M0 vs M3	39.110	<0.001	-
	M1a vs M2a	16.940	<0.001	*48Q*, *135A*, **145 T**
	M7 vs M8	17.367	<0.001	*48Q*, *135A*, **145 T**
	M8a vs M8	16.931	<0.001	See M7 vs M8 comparison

LRT values, p-values and positively selected sites identified by the BEB (p < 0.01 (in bold) and p < 0.05 (in italic); CodeML) are shown.

None of the performed LTRs of the branch-site analyses were significant (1 ≥ p ≥ 0.20) indicating that although the *ω* ratios do vary among sites (see above), the *ω* ratios do not vary significantly among lineages.

### Amino acid substitutions and their predicted effect on function

Next, the individual amino acid substitutions were examined in more detail. The total protein length and the number of amino acid substitutions per locus are shown in Table 5 (see also Figure 2 and Additional file 6). A relative large number of substitutions was observed in *dlx2a* (13), *dlx3a* (16) and *dlx4b* (10), while in *dlx5a* no substitution was found. Most of the amino acid substitutions are species-specific (i.e., observed in a single species), although lineage-specific substitutions were observed for the lamprologines (*dlx2a*, *dlx3a*, *dlx4b*), ectodines (*dlx2a*) and haplochromines (*dlx2a*, *dlx3a*). None of the observed amino acid substitutions have a predicted effect on the protein functions (see Table 5), although two substitutions were observed in the homeobox domain of *dlx2a* (Figure 2).

**Table 5:** **Amino acid substitution and their predicted effect on function for the eight cichlid *dlx *loci**

**Locus**	**L**	**S**	**E**
*dlx1a*	144	1	Tolerated
*dlx2a*	255	13	Tolerated
*dlx3a*	222	16	Tolerated
*dlx3b*	160	4	Tolerated
*dlx4a*	96	1	Tolerated
*dlx4b*	167	10	Tolerated
*dlx5a*	271	0	-
*dlx6a*	236	2	Tolerated

Length of obtained protein sequence (L), the number of amino acid substitutions (S) and the predicted effect of the substitutions on the function generated by the SIFT analyses (E) are shown.

### Selection regimes on the *dlx* clusters

It is known that the paired members of each of the four *dlx* clusters (Additional file 4) are transcribed concurrently [35,42-45]. To characterize if the members of the same *dlx* cluster evolved at similar rates and under similar selection regimes, we had a closer inspection of these paired genes. First, the teleost dlx protein and cichlid gene trees show that overall and relative tree lengths (or the rate of evolution) differ between the two genes within a cluster. Loci with the highest (*dlx3a*: 0.583/0.910 and *dlx4b*: 0.864/0.937) or the smallest (*dlx1a*: 0.366/0.483 and *dlx6a*: 0.227/0.542) tree lengths are never observed within the same cluster. Furthermore, the mode of selection seems to differ between members of the same dlx clusters as well. While strong purifying selection was observed for *dlx3b*, *dlx4a* and *dlx5a*, their paired cluster members *dlx4b, dlx3a* and *dlx6a* show sign of elevated *ω*-values. A notable exception to this observation is the *dlx1a-dlx2a* cluster. For both genes a proportion of sites was found with elevated *ω*-values (note that the proportion is considerably bigger for *dlx2a*). These observations indicate that although clusters are transcribed concurrently, selection seems to act on the individual gene level rather than on the level of the *dlx* gene clusters. Also the observed patterns are not in concordance with the two groups of homeobox domains that emerged from the initial tandem duplication (see [52] and Additional file 4).

## Discussion

In this work, we present a detailed evolutionary characterization of the *dlx* gene repertoire in East African cichlid fishes. Previously, Renz *et al.*[35] studied the embryonic expression patterns of *dlx* genes in cichlids and showed that they are expressed in e.g., the developing jaw apparatus and anal fin, tissues that contribute to two putative evolutionary innovations: the pharyngeal jaw and the egg-spots on the anal fin of the cichlid *A. burtoni*. Here, we study the molecular evolution of *dlx* genes in a representative set of 23 East African cichlid species. We performed comparative phylogenetic analyses and detailed screens of nonsynonymous-synonymous substitution rate ratios to determine the selective pressure acting upon these candidate genes for evolutionary novelties in cichlid fishes.

### *Dlx3a* did not get lost in the cichlid lineage

Our phylogenetic analyses of dlx proteins extends previous analyses (e.g., [35]) by the inclusion of cod [72] and four different cichlid species (i.e., *O. niloticus*, *N. brichardi*, *A. burtoni* and *P. nyererei*; BROAD Institute). Although our results agree with most of the available hypotheses on the evolutionary loss of *dlx* genes in specific teleost lineages (i.e., *dlx3a* in zebrafish and *dlx4a* in medaka), we did detect *dlx3a* in cichlids and thus refute the cichlid-specific gene loss hypothesis of *dlx3a* put forward by Renz *et al.*[35]. Not only were we able to locate this gene in all four cichlid genomes examined (Additional file 1), we also gathered partial gene sequences for this locus in all 23 cichlid species included (Additional files 4 and 6). Furthermore, in-house tblastx searches of this newly identified paralog against preliminary cichlid EST libraries (BROAD Institute, unpublished data) resulted in multiple hits, providing proof of its expression in – at least - *Astatotilapia burtoni*, *Oreochromis niloticus* and *Metriaclima zebra*.

### Selection on *dlx* paralogs in relation to gene duplication events

Gene-wide estimates of the *d*_N_/*d*_S_ ratios indicate that all loci evolved under purifying selection (*ω* < 1), indicating strong selection against deleterious mutations, commonly observed in functional proteins. Additional analyses of individual codons indicate that the sequenced regions of *dlx3b*, *dlx4a* and *dlx5a* evolved under purifying selection, while positive selection acting on specific codons was detected for a small proportion of sites (i.e., up to 12%) for *dlx1a*, *dlx2a*, *dlx3a* and *dlx4b* (i.e., a smaller number of positively selected sites was found with more stringent SLR analyses for *dlx2a*, *dlx3a* and *dlx4b*). Plausible reasons for the excess of nonsynonymous mutations in these loci are either lowered functional constraints or directional selection, as Sumiyama and colleagues suggested for *Dlx7* in mouse [73]. Different modes of selection are thus found to have acted on the *dlx* paralogs in cichlids after the genome duplication events.

Differential selection after gen(om)e duplication is a commonly observed phenomenon and is associated with the fate of the gene duplicates i.e., non-, sub- or neofunctionalization. Sub- and neofunctionalization are adaptive processes by which either spatial or temporal partitioning of the ancestral function or the evolution of complete new functions take place [5,74-76]. While ancestral functions can be maintained by retaining the protein sequences and preventing deleterious mutations through purifying selection, relaxed selection on the other duplicate can lead to the introduction of mutations and subsequent divergence [5,75,76]. Most of these changes are deleterious and are followed by the loss of the gene over time (i.e., nonfunctionalization). On rare occasions the mutations can lead to an altered function of the protein (i.e., neofunctionalization; change within the protein) or altered expression pattern (subfunctionalization; change in regulatory regions), which can be characterized by elevated *ω* values and the maintenance of the mutations results in divergence of the two duplicates.

Many studies have focused on duplicated genes in relation to divergence of duplicates (see e.g., [77-80] and references therein). An interesting case of subfunctionalization was described in leaf-eating Colobine monkeys, in which the pancreatic ribonuclease gene (*RNASE1*), necessary to digest its specialized diet, was duplicated [81,82]. Although the two gene-products are used in the same process (i.e., digestion of bacterial RNA), the duplicate gene shows many substitutions, while the ancestral locus did not change [81]. Similar patterns of heterogeneity in amino acid substitutions or differential selection were also observed by Dermitzakis and Clark [83] between duplicates of several developmental gene families (e.g., *Notch*, *Bmp* and *Hox9*) in mouse and human. Interestingly, differential selection regimes acting on paralogs were also found in the murine *Dlx3*-*Dlx*7 cluster, with *Dlx7* evolving more rapidly than *Dlx3*[73]. Our results of differential selection acting on the cichlid *dlx* paralogs are thus comparable to previously studied cases of duplicated genes. We even detect a similar pattern as Sumiyama *et al.*[73], with *dlx4b* evolving more rapidly than *dlx3b* (i.e., relative tree length 0.937 vs 0.609).

The adaptive protein evolution as observed in *dlx1a*, *dlx2a*, *dlx3a* and *dlx4b* together with the evolutionary history of the gene family, could thus be a sign of possible new or altered functions of these *dlx* paralogs in cichlids. Although we did not observe amino acid substitutions with predicted apparent effect on the protein function in our partial sequences, other mechanisms, such as *cis*-regulatory evolution might have altered the expression patterns after gene duplication. Gene expression analyses in cichlid and zebrafish indicate that clusters are often transcribed concurrently and that the *dlx* duplicates exhibit overlapping expression patterns in particular during the development of brain and pharyngeal arches [35,44,45]. This co-expression of the *dlx* clusters is controlled through intergenic *cis*-regulatory regions [35,42,43]. While mutations in these regions are expected to affect the expression of both paralogs, changes in the coding regions of the *dlx* loci are likely to affect the individual *dlx* locus’ function, which could lead to neofunctionalization.

### Selection pressure on *dlx* paralogs in relation to evolutionary innovations

We found an interesting pattern comparing our *d*_N_/*d*_S_ results with the expression patterns found by Renz *et al.*[35] in relation to evolutionary novelties in cichlids. In the developing pharyngeal teeth and the anal fin *dlx3b, dlx4a* (not in anal fin) and *dlx5a*, the exact loci for which we found strong patterns of purifying selection, are expressed at high levels. Although this observation seems to contradict other cases in which candidate genes showed accelerated rates of protein evolution (see [31,53,84]), they do not stand alone (see e.g., [36]). It has been shown that minor changes in the complex genetic pathways underlying the development of morphological structures can lead to the evolution of novelties (see e.g., [85]). Furthermore, many cases of morphological adaptation are driven by *cis*-regulatory evolution (reviewed in [86]). Several intergenic *cis*-regulatory elements have been identified in the *dlx* clusters in *A. burtoni* by Renz *et al.*[35], but the functional characterization in cichlids is yet to be performed. It is thus possible that only a small fraction of genes involved in the evolutionary novelties in cichlids show signs of adaptive evolution and that the three *dlx* loci were co-opted for their ancestral functions.

According to Renz *et al.*[35], the five *dlx* genes for which we found signatures of positive selection, are either not expressed at all or at low levels during pharyngeal teeth and anal fin development in the cichlid *A. burtoni*. Low levels of gene expression were observed for *dlx2a* in the developing pharyngeal teeth in cichlids [35], while higher *dlx2a* expression levels were observed in other teleost species [33,44,45]. *Dlx4b* and *dlx6a* expression has previously been shown in the developing pharyngeal teeth of zebrafish and/or medaka [44,45], but has not been observed in cichlids (yet). Furthermore, multiple *dlx* genes, including loci with signatures of positive selection, appear to be expressed in the developing anal fin tissue at time points coinciding with egg-spot development in *A. burtoni* (E. Santos, personal communications). Therefore, it is likely that several *dlx* paralogs, for which we found signs of positive selection, are involved in the development of evolutionary innovations in cichlids, in contrast to the initial findings of Renz *et al.*[35]. Future detailed and extended functional analyses should be conducted to elucidate their role in the development of these evolutionary important traits in cichlid fishes.

## Conclusions

In this study, we provide an in depth molecular evolutionary analysis of the *dlx* gene repertoire in teleost fishes. We located and generated partial sequences for *dlx3a* in 23 East African cichlid species, refuting the hypothesis of Renz *et al.*[35] that *dlx3a* got lost in the cichlid lineage. Phylogenetic analyses of the teleost *dlx* gene repertoire show that substantial differences exist in the rate of evolution among teleost *dlx* paralogs. In addition, analyses of the nonsynonymous-synonymous substitution rates of the cichlid *dlx* paralogs revealed strong differences in the selection pressure acting upon *dlx* paralogs and cluster members. Although differential selection pressure after gene duplication is a putative sign of new or altered functions, we observed a link between the *dlx* loci under strong purifying selection, in particular, and high expression levels in two cichlids’ novelties; the pharyngeal jaw and anal fin. This indicates that other mechanisms than adaptive protein evolution are likely to be involved in the co-option of these genes. Furthermore, several (preliminary) studies found that at least three other *dlx* paralogs, for which we found signs of positive selection, are actually expressed in the developing pharyngeal teeth and/or haplochromine anal fin. Hence, the *dlx* paralogs appear as candidate genes for the development of evolutionary innovations in cichlids, although further functional analyses should elucidate the role of positive selection therein.

## Availability of supporting data

The datasets supporting the results of this article are publicly available in the GenBank repository under accession numbers: KC285366-KC285546 and in the treeBASE repository under submission number 14433, http://purl.org/phylo/treebase/phylows/study/TB2:S14433.

## Competing interest

The authors declare that they have no competing interests.

## Authors’ contributions

ETD, FDK and WS conceived the study. FDK generated the data. ETD and FDK analyzed the data. ETD and WS wrote the paper. All authors read and approved the final manuscript.

## Authors’ information

ETD is a PhD student and FDK a master student in the group of WS. WS is a Professor of Zoology and Evolutionary Biology at the University of Basel. The research of his team focuses on the genetic basis of adaptation, evolutionary innovation and animal diversification of mainly the exceptionally diverse cichlid fishes.

## Supplementary Material

Additional file 1 **Accession numbers and/or genomic location of the teleost *dlx *sequences.** Click here for file

Additional file 2 Specimen information and GenBank Accession numbers. Click here for file

Additional file 3 Primer information and primer sequences. Click here for file

Additional file 4 **Protein comparison of the teleost dlx homeobox domains.** Depicted are the amino acid sequences of the homeobox domains for each of the four teleost clusters: dlx1a-dlx2a, dlx4a-dlx3a, dlx4b-dlx3b and dlx6a-dlx5a in comparison with the single Dll homeobox sequence (here depicted in duplo) of *Drosophila melanogaster*. Sequences can be divided in two groups; dlx1a, dlx4a, dlx4b and dlx6a versus dlx2a, dlx3a, dlx3b and dlx5a. The two sixty amino acid long homeobox domains of each cluster are depicted in separate boxes. The top graph displays the mean pairwise identity of all sequences (i.e., green = 100% identity and brown ≥ 30% identity). Numbers represent the amino acid position within the homeobox. Click here for file

Additional file 5 **Maximum likelihood gene trees based on 23 cichlid species for the eight *dlx *loci.** Bootstrap values (PAUP*) and Bayesian posterior probabilities (MrBayes) above 50% are shown respectively above and below the branches. A color key for the ten studied cichlid lineages is given in the box below the figure. (**a**) *Dlx1a* (737 base pairs (bp); TPM3uf model). Two major polytomies were recovered. The lamprologines cluster together with the Boulengerochromini, Bathybatini and the Cyphotilapiini. *A. burtoni* is found at the base with *O. tanganicae*. (**b**) *Dlx2a* (1371 bp; HKY + I model). Polytomous tree with all members of the lineages Lamprologines, Ectodines, Haplochromines and Limnochromines recovered as monophyletic clades. (**c**) *Dlx3a* (666 bp; HKY model). Polytomous tree, with only the Lamprologines recovered as monophyletic clade. (**d**) *Dlx4a* (1166 bp; TPM3uf + I + G). Polytomous relationships were observed between multiple lineages, although most lineages are monophyletic except the Haplochromines (**e**) *Dlx3b* (1972 bp; GTR + I + G). Moderately resolved tree. (**f**) *Dlx4b* (722 bp; TPM3uf). Mostly polytomous relationships between species, except the Limnochromini and most members of the Lamprologines. (**g**) *Dlx5a* (1538 bp; TIM2 + G). Basal polytomy divides ingroup species except *G. permaxillaris*, in two big clades. (**h**) *Dlx6a* (1710 bp; TIM3 + G). Limnochromines, Lamprologines and Haplochromines recovered as monophyletic clades, although the relationships between lineages are largely polytomous. Click here for file

Additional file 6 **Four partially sequenced cichlid Dlx proteins.** Depicted are the amino acid sequences of *Astatotilapia burtoni* (**a**, **c**, **d**) and *Ctenochromis horei* (**b**). Secondary structure predictions were obtained from the PSIPRED server (http://bioinf.cs.ucl.ac.uk/psipred/). (**a**) Dlx3b. (**b**) Dlx4a. (**c**) Dlx5a. (**d**) Dlx6a. Click here for file
