# Supplementary material for: Sequence analyses of the distal-less homeobox gene family in East African cichlid fishes reveal signatures of positive selection
Source: BMC Evol Biol. 2013 Jul 17;13:153. doi: 10.1186/1471-2148-13-153 (PMC3728225; doi:10.1186/1471-2148-13-153)
Supplement: Additional file 1 — Accession numbers and/or genomic location of the teleost dlx sequences. [file 1471-2148-13-153-S1.doc]

**Additional File 1**

**Ensemble/EMBL accession numbers or genomic location of teleost *dlx* sequences**

| **Species** | **Ensemble/GenBank accession numbers and preliminary genome information** | | | |
| --- | --- | --- | --- | --- |
|  | ***dlx1a*** | ***dlx2a*** | ***dlx3a*** | ***dlx3b*** |
| *Danio rerio* | ENSDARG00000013125 | ENSDARG00000079964 | - | ENSDARG00000014626 |
| *Gradus morhua* | ENSGMOG00000002250 | ENSGMOG00000002233 | ENSGMOG00000018564 | ENSGMOG00000013282 |
| *Gasterosteus aculeatus* | ENSGACG00000004931 | ENSGACG00000004914 | ENSGACG00000005117 | ENSGACG00000009888 |
| *Tetraodon nigroviridis* | ENSTNIG00000016938 | ENSTNIG00000016939 | ENSTNIG00000011165 | ENSTNIG00000012856 |
| *Takifugu rubripes* | ENSTRUG00000017741 | ENSTRUG00000017732 | ENSTRUG00000007563 | ENSTRUG00000016555 |
| *Oryzias latipes* | ENSORLG00000017364 | ENSORLG00000017372 | scaffold784^3^ | ENSORLG00000004116 |
| *Oreochromis niloticus* | ENSONIG00000008725 | ENSONIG00000008722 | ENSONIG00000006437 | ENSONIG00000019897 |
| *Astatotilapia burtoni*^1^ | scaffold_41 | scaffold_41 | scaffold_363 | scaffold_149 |
| *Astatotilapia burtoni*^2^ | FN667596 | FN667597 | - | FN667598 |
| *Neolamprologus brichardi*^1^ | scaffold_13 | scaffold_13 | scaffold_134 | scaffold_76 |
| *Pundamilia nyererei*^1^ | scaffold_8 | scaffold_8 | scaffold_294 | scaffold_4 |

**Additional File 1** **cont.**

| **Species** | **Ensemble/GenBank accession numbers and preliminary genome information** | | | |
| --- | --- | --- | --- | --- |
|  | ***dlx4a*** | ***dlx4b*** | ***dlx5a*** | ***dlx6a*** |
| *Danio rerio* | ENSDARG00000011956 | ENSDARG00000071560 | ENSDARG00000042296 | ENSDARG00000042291 |
| *Gradus morhua* | ENSGMOG00000018574 | ENSGMOG00000013275 | ENSGMOG00000013639 | ENSGMOG00000013631 |
| *Gasterosteus aculeatus* | ENSGACG00000005120 | ENSGACG00000009881 | ENSGACG00000006792 | ENSGACG00000006780 |
| *Tetraodon nigrovirdis* | ENSTNIG00000011166 | ENSTNIG00000012857 | ENSTNIG00000017761 | ENSTNIG00000017762 |
| *Takifugu rubripes* | ENSTRUG00000007722 | ENSTRUG00000016558 | ENSTRUG00000000270 | ENSTRUG00000007566 |
| *Oryzias latipes* | - | ENSORLG00000004106 | ENSORLG00000004561 | ENSORLG00000004542 |
| *Oreochromis niloticus* | ENSONIG00000006433 | ENSONIG00000019896 | ENSONIG00000010755 | ENSONIG00000010756 |
| *Astatotilapia burtoni*^1^ | scaffold_363 | scaffold_149 | scaffold_97 | scaffold_97 |
| *Astatotilapia burtoni*^2^ | FN667600 | FN667599 | FN667601 | FN667602 |
| *Neolamprologus brichardi*^1^ | scaffold_134 | scaffold_76 | scaffold_12 | scaffold_12 |
| *Pundamilia nyererei*^1^ | scaffold_294 | scaffold_4 | scaffold_29 | scaffold_29 |

^1^ v1 assembly of the preliminary whole genome sequences by the Cichlid Genome Consortium (cichlid.umd.edu/CGCindex.html)

^2^ cDNA sequences by Renz *et al*

^3^ Identified by Debiais-Thibaud *et al*. (2008); uncharacterized gene/protein could not be retrieved in Ensemble and uniprot
