# Supplementary material for: Sequence analyses of the distal-less homeobox gene family in East African cichlid fishes reveal signatures of positive selection
Source: BMC Evol Biol. 2013 Jul 17;13:153. doi: 10.1186/1471-2148-13-153 (PMC3728225; doi:10.1186/1471-2148-13-153)
Supplement: Additional file 2 — Specimen information and GenBank Accession numbers. [file 1471-2148-13-153-S2.doc]

**Additional Table 2**

Specimen information and GenBank accession numbers

| **Species** | **Tribe** | **GenBank accession numbers**  **Dlx1a** **Dlx2a** **Dlx3a** **Dlx3b**  **Dlx4a** **Dlx4b** **Dlx5a Dlx6a** |
| --- | --- | --- |
| *Bathybates graueri* | Bathybatini | KC285376 KC285403 KC285413 KC285454  KC285458 KC285481 KC285513 KC285526 |
| *Boulengerochromis microlepis* | Boulengerochromini | KC285381 KC285404 KC285414 KC285440  KC285459 KC285482 KC285518 KC285527 |
| *Cyphotilapia frontosa* | Cyphotilapiini | KC285382 KC285397 KC285415 KC285441  KC285462 KC285484 KC285519 KC285528 |
| *Cyprichromis leptosoma* | Cyprichromini | KC285383 KC285398 KC285418 KC285442  KC285463 KC285485 KC285520 KC285529 |
| *Callochromis macrops* | Ectodini | KC285384 KC285399 KC285419 KC285443  KC285479 KC285493 KC285521 - |
| *Cyathopharynx furcifer* | Ectodini | KC285385 KC285400 KC285416 KC285444  KC285461 KC285483 KC285503 KC285530 |
| *Astatotilapia burtoni* | Haplochromini | KC285386 KC285401 KC285411 KC285445  KC285478 KC285480 KC285522 KC285531 |
| *Ctenochromis horei* | Haplochromini | KC285387 KC285402 KC285417 KC285446  KC285460 KC285496 KC285523 KC285532 |
| *Altolamprologus fasciatus* | Lamprologini | KC285373 KC285393 KC285412 KC285437  KC285457 KC285502 KC285510 KC285540 |
| *Lepidiolamprologus elongatus* | Lamprologini | KC285374 KC285394 KC285422 KC285438  KC285465 KC285489 KC285511 KC285541 |
| *Neolamprologus furcifer* | Lamprologini | KC285388 KC285406 KC285425 KC285450  KC285468 KC285490 KC285524 KC285533 |
| *Neolamprologus pulcher* | Lamprologini | KC285366 KC285407 KC285426 KC285451  KC285469 KC285491 KC285504 KC285534 |
| *Variabilichromis moorii* | Lamprologini | KC285375 KC285395 KC285433 KC285453  KC285477 KC285492 KC285512 KC285542 |
| *Gnathochromis permaxillaris* | Limnochromini | KC285377 KC285410 KC285420 KC285455  KC285464 KC285501 KC285514 KC285543 |
| *Limnochromis staneri* | Limnochromini | KC285367 KC285389 KC285424 KC285434  KC285466 KC285486 KC285505 KC285535 |
| *Perissodus microlepis* | Perissodini | KC285368 - KC285430 KC285452  KC285471 KC285487 KC285506 KC285536 |
| *Plecodus straeleni* | Perissodini | KC285369 KC285408 KC285431 KC285447  KC285473 KC285488 KC285507 - |
| *Oreochromis tanganicae* | Tilapiini | KC285378 KC285409 KC285427 KC285456  KC285470 KC285494 KC285515 KC285544 |
| *Limnotilapia dardenii* | Tropheini | KC285372 KC285392 KC285421 KC285436  KC285476 KC285495 KC285509 KC285539 |
| *Lobochilotes labiatus* | Tropheini | KC285370 KC285390 KC285423 KC285449  KC285467 KC285497 KC285525 KC285537 |
| *Petrochromis famula* | Tropheini | KC285379 KC285405 KC285429 KC285448  KC285472 KC285498 KC285516 KC285545 |
| *Pseudosimochromis curvifrons* | Tropheini | KC285380 KC285396 KC285428 KC285439  KC285474 KC285499 KC285517 KC285546 |
| *Tropheus moori* | Tropheini | KC285371 KC285391 KC285432 KC285435  KC285475 KC285500 KC285508 KC285538 |
