# Supplementary material for: Sequence analyses of the distal-less homeobox gene family in East African cichlid fishes reveal signatures of positive selection
Source: BMC Evol Biol. 2013 Jul 17;13:153. doi: 10.1186/1471-2148-13-153 (PMC3728225; doi:10.1186/1471-2148-13-153)
Supplement: Additional file 3 — Primer information and primer sequences. [file 1471-2148-13-153-S3.doc]

**Additional File 3**

**Primer Sequences**

| **Locus** | **Forward sequence 5’ - 3’** | **Reverse sequence 5’ - 3’** |
| --- | --- | --- |
| ***dlx1a*** | CCGTCCTTCACTGAGATCAT | CTCCGGTAGCGCCAAATAC |
| ***dlx2a*** | CTCTCCCCCAGTTCCAAGAT | TTTCTGGCTCGCTCTCATCT |
|  | TCGAACCAGATTACCTCAA | TACACCCGACGTTTTCTCGT |
| ***dlx3a*** | gttccatctcggcttcca | acctcyctgttgaatgct |
|  | gtcgctccaagtttaagaag | agtagacggctcccatactc |
| ***dlx3b*** | TGCGAGTATTTTGACCGATCT | CGAGGTATTGTGCCTTCTGG |
|  | AGGTTCCCTTGGAGCACAG | ACAGTGCAGTCGTTCCGTTT |
| ***dlx4a*** | TTACAACAAACCGGGAATCG | GGACTGTGGTAGGGTGGGTA |
|  | GGACTGTGGTAGGGTGGGTA | CCTCCTCATACAAATGAAAAGC |
| ***dlx4b*** | CTGTTCGTGATGTAAAGACG | GCTGTTGCTGTGATTGCTGT |
|  | GCGCTCCAAGTACAAAAAGA | TTTTATTCTCGCACGGCATT |
| ***dlx5a*** | ACGCGCTCTCTCTCGAAGTA | GAGGTGAGGATGGTGAATGG |
|  | GAGGTGAGGATGGTGAATGG | GACTGCCATCAAATACATTGC |
| ***dlx6a*** | AGTGCTCTCAAGGCAGAAAA | TCCGGTAAAGCGAGGTACTG |
|  | AGCAGCAGAAGACGACAGTG | ACCTTGCTTTCGTCGTCAGT |
