# Supplementary material for: Sequence analyses of the distal-less homeobox gene family in East African cichlid fishes reveal signatures of positive selection
Source: BMC Evol Biol. 2013 Jul 17;13:153. doi: 10.1186/1471-2148-13-153 (PMC3728225; doi:10.1186/1471-2148-13-153)
Supplement: Additional file 4 — Protein comparison of the teleost dlx homeobox domains. Depicted are the amino acid sequences of the homeobox domains for each of the four teleost clusters: dlx1a-dlx2a, dlx4a-dlx3a, dlx4b-dlx3b and dlx6a-dlx5a in comparison with the single Dll homeobox sequence (here depicted in duplo) of Drosophila melanogaster. Sequences can be divided in two groups; dlx1a, dlx4a, dlx4b and dlx6a versus dlx2a, dlx3a, dlx3b and dlx5a. The two sixty amino acid long homeobox domains of each cluster are depicted in separate boxes. The top graph displays the mean pairwise identity of all sequences (i.e., green = 100% identity and brown ≥ 30% identity). Numbers represent the amino acid position within the homeobox. [file 1471-2148-13-153-S4.doc]

**Additional Table 4**

**Input tree file and sequence files (fasta format) for the PAML analyses**

Input tree file (based on a concatenated dataset of 9.2 kb):

((((((((((((Npul:0.00148,(Vmoo:0.003181,Nfur:0.003007):0.000445):0.002076,(Afas:0.003877,Lelo:0.002103):0.002711):0.004143,(((Bgra:0.005906,Otan:0.014106):0.000638,Bmic:0.002503):0.001119,Cfro:0.002355):0.000394):0.000325,(Lsta:0.004174,Gper:0.002544):0.001405):0.000644,((Pmic:0.001325,Pstr:0.000914):0.002134,Clep:0.00461):0.001363):0.000718,(Cmac:0.003115,Cfur:0.003039):0.002852):0.001739,Abur:0.002978):0.001436,Tmoo:0.002356):0.000786,Llab:0.001519):0.000563,Pfam:0.001172):0.000257,Chor:0.002166):0.000139,Ldar:0.000754,Pcur:0.002183)

Dlx1a input file:

>dlx1a_Neopul

GGGGAACCGCCAGAAGAGATGACAATGACTACGATTCCAGAAAGTCTTAATAGCCCTGCC

TCAGGAAAAACCGTTTTCATGGAGTTTGGACCCCCGAGTCAACAAATGTCGCCTTCCTCC

ATGTCTCACGGACACTATCCCATGCACTGTTTACATTCTGCAGGTCACACACAGCACGAC

AGCTACAGTCCAGCCTCGTCGTTCCCCAGATCCTTGGGTTATCCATACGTAAACTCCGTC

GGCAGCCATTCCACCAGTCCATATCTCAGCACAGTACAGACTTACCAAAACAGCTCGGGA

CTCACGCAGACACGATTAGAGGATGCAGCGCCAGAAACAGAGAAAAACACAGTGGTGGAA

GGCGGAGAGGTGCGCTTCAACGGCAAAGGGAAAAAGATTAGAAAACCAAGGACTATATAT

TCGAGTTTACAA

>dlx1a_Limsta

GGGGAACCGCCAGAAGAGATGACAATGACTACGATTCCAGAAAGTCTTAATAGCCCTGCC

TCAGGAAAAACCGTTTTCATGGAGTTTGGACCCCCGAGTCAACAAATGTCGCCTTCCTCC

ATGTCTCACGGACACTATCCCATGCACTGTTTACATTCTGCAGGTCACACACAGCACGAC

AGCTACAGTCCAGCCTCGTCGTTCCCCAGATCCTTGGGTTATCCATACGTAAACTCCGTC

GGCAGCCATTCCACCAGTCCATATCTCAGCACAGTACAGACTTACCAAAACAGCTCGGGA

CTCACGCAGACACGATTAGAGGATGCAGCGCCAGAAACAGAGAAAAACACAGTGGTGGAA

GGCGGAGAGGTGCGCTTCAACGGCAAAGGGAAAAAGATTAGAAAACCAAGGACTATATAT

TCGAGTTTACAA

>dlx1a_Permic

GGGGAACCGCCAGAAGAGATGACAATGACTACGATTCCAGAAAGTCTTAATAGCCCTGCC

TCAGGAAAAACCGTTTTCATGGAGTTTGGACCCCCGAGTCAACAAATGTCGCCTTCCTCC

ATGTCTCACGGACACTATCCCATGCACTGTTTACATTCTGCAGGTCACACACAGCACGAC

AGCTACAGTCCAGCCTCGTCGTTCCCCAGATCCTTGGGTTATCCATACGTAAACTCCGTC

GGCAGCCATTCCACCAGTCCATATCTCAGCACAGTACAGACTTACCAAAACAGCTCGGGA

CTCACACAGACACGATTAGAGGATGCAGCGCCAGAAACAGAGAAAAACACAGTGGTTGAA

GGCGGAGAGGTGCGCTTCAACGGCAAAGGGAAAAAGATTAGAAAACCAAGGACTATATAT

TCGAGTTTACAA

>dlx1a_Plesta

GGGGAACCGCCAGAAGAGATGACAATGACTACGATTCCAGAAAGTCTTAATAGCCCTGCC

TCAGGAAAAACCGTTTTCATGGAGTTTGGACCCCCGAGTCAACAAATGTCGCCTTCCTCC

ATGTCTCACGGACACTATCCCATGCACTGTTTACATTCTGCAGGTCACACACAGCACGAC

AGCTACAGTCCAGCCTCGTCGTTCCCCAGATCCTTGGGTTATCCATACGTAAACTCCGTC

GGCAGCCATTCCACCAGTCCATATCTCAGCACAGTACAGACTTACCAAAACAGCTCGGGA

CTCACGCAGACACGATTAGAGGATGCAGCGCCAGAAACAGAGAAAAACACAGTGGTTGAA

GGCGGAGAGGTGCGCTTCAACGGCAAAGGGAAAAAGATTAGAAAACCAAGGACTATATAT

TCGAGTTTACAA

>dlx1a_Loblab

GGGGAACCGCCAGAAGAGATGACAATGACTACGATTCCAGAAAGTCTTAATAGCCCTGCC

TCAGGAAAAACCGTTTTCATGGAGTTTGGACCCCCGAGTCAACAAATGTCGCCTTCCTCC

ATGTCTCACGGACACTATCCCATGCACTGTTTACATTCTGCAGGTCACACACAGCACGAC

AGCTACAGTCCAGCCTCGTCGTTCCCCAGATCCTTGGGTTATCCATACGTAAACTCCGTC

GGCAGCCATTCCACCAGTCCATATCTCAGCACAGTACAGACTTACCAAAACAGCTCGGGA

CTCACGCAGACACGATTAGAGGATGCAGCGCCAGAAACAGAGAAAAACACAGTGGTGGAA

GGCGGAGAGGTGCGCTTCAACGGCAAAGGGAAAAAGATTAGAAAACCAAGGACTATATAT

TCGAGTTTACAA

>dlx1a_Tromoo

GGGGAACCGCCAGAAGAGATGACAATGACTACGATTCCAGAAAGTCTTAATAGCCCTGCC

TCAGGAAAAACCGTTTTCATGGAGTTTGGACCCCCGAGTCAACAAATGTCGCCTTCCTCC

ATGTCTCACGGACACTATCCCATGCACTGTTTACATTCTGCAGGTCACACACAGCACGAC

AGCTACAGTCCAGCCTCGTCGTTCCCCAGATCCTTGGGTTATCCATACGTAAACTCCGTC

GGCAGCCATTCCACCAGTCCATATCTCAGCACAGTACAGACTTACCAAAACAGCTCGGGA

CTCACGCAGACACGATTAGAGGATGCAGCGCCAGAAACAGAGAAAAACACAGTGGTGGAA

GGCGGAGAGGTGCGCTTCAACGGCAAAGGGAAAAAGATTAGAAAACCAAGGACTATATAT

TCGAGTTTACAA

>dlx1a_Tylpol

GGGGAACCGCCAGAAGAGATGACAATGACTACGATTCCAGAAAGTCTTAATAGCCCTGCC

TCAGGAAAAACCGTTTTCATGGAGTTTGGACCCCCGAGTCAACAAATGTCGCCTTCCTCC

ATGTCTCACGGACACTATCCCATGCACTGTTTACATTCTGCAGGTCACACACAGCACGAC

AGCTACAGTCCAGCCTCGTCGTTCCCCAGATCCTTGGGTTATCCATACGTAAACTCCGTC

GGCAGCCATTCCACCAGTCCATATCTCAGCACAGTACAGACTTACCAAAACAGCTCGGGA

CTCACGCAGACACGATTAGAGGATGCAGCGCCAGAAACAGAGAAAAACACAGTGGTGGAA

GGCGGAGAGGTGCGCTTCAACGGCAAAGGGAAAAAGATTAGAAAACCAAGGACTATATAT

TCGAGTTTACAA

>dlx1a_Altfas

GGGGAACCGCCAGAAGAGATGACAATGACTACGATTCCAGAAAGTCTTAATAGCCCTGCC

TCAGGAAAAACCGTTTTCATGGAGTTTGGACCCCCGAGTCAACAAATGTCGCCTTCCTCC

ATGTCTCACGGACACTATCCCATGCACTGTTTACATTCTGCAGGTCACACACAGCACGAC

AGCTACAGTCCAGCCTCGTCGTTCCCCAGATCCTTGGGTTATCCATACGTAAACTCCGTC

GGCAGCCATTCCACCAGTCCATATCTCAGCACAGTACAGACTTACCAAAACAGCTCGGGA

CTCACGCAGACACGATTAGAGGAGGCAGCGCCAGAAACAGAGAAAAACACAGTGGTGGAA

GGCGGAGAGGTGCGCTTCAACGGCAAAGGGAAAAAGATTAGAAAACCAAGGACTATATAT

TCGAGTTTACAA

>dlx1a_Lepelo

GGGGAACCGCCAGA--AGATGACAATGACTACGATTCCAGAAAGTCTTAATAGCCCTGCC

TCAGGAAAAACCGTTTTCATGGAGTTTGGACCCCCGAGTCAACAAATGTCGCCTTCCTCC

ATGTCTCACGGACACTATCCCATGCACTGTTTACATTCTGCAGGTCACACACAGCACGAC

AGCTACAGTCCAGCCTCGTCGTTCCCCAGATCCTTGGGTTATCCATACGTAAACTCCGTC

GGCAGCCATTCCACCAGTCCATATCTCAGCACAGTACAGACTTACCAAAACAGCTCGGGA

CTCACGCAGACACGATTAGAGGAGGCAGCGCCAGAAACAGAGAAAAACACAGTGGTGGAA

GGCGGAGAGGTGCGCTTCAACGGCAAAGGGAAAAAGATTAGAAAACCAAGGACTATATAT

TCGAGTTTACAA

>dlx1a_Varmoo

GGGGAACCGCCAGAAGAGATGACAATGACTACGATTCCAGAAAGTCTTAATAGCCCTGCC

TCAGGAAAAACCGTTTTCATGGAGTTTGGACCCCCGAGTCAACAAATGTCGCCTTCCTCC

ATGTCTCACGGACACTATCCCATGCACTGTTTACATTCTGCAGGTCACACACAGCACGAC

AGCTACAGTCCAGCCTCGTCGTTCCCCAGATCCTTGGGTTATCCATACGTAAACTCCGTC

GGCAGCCATTCCACCAGTCCATATCTCAGCACAGTACAGACTTACCAAAACAGCTCGGGA

CTCACGCAGACACGATTAGAGGATGCAGCGCCAGAAACAGAGAAAAACACAGTGGTGGAA

GGCGGAGAGGTGCGCTTCAACGGCAAAGGGAAAAAGATTAGAAAACCAAGGACTATATAT

TCGAGTTTACAA

>dlx1a_Batgra

GGGGAACCGCCAGAAGAGATGACAATGACTACGATTCCAGAAAGTCTTAATAGCCCTGCC

TCAGGAAAAACCGTTTTCATGGAGTTTGGACCCCCGAGTCAACAAATGTCGCCTTCCTCC

ATGTCTCACGGACACTATCCCATGCACTGTTTACATTCTGCAGGTCACACACAGCACGAC

AGCTACAGTCCAGCCTCGTCGTTCCCCAGATCCTTGGGTTATCCATACGTAAACTCCGTC

GGCAGCCATTCCACCAGTCCATATCTCAGCACAGTACAGACTTACCAAAACAGCTCGGGA

CTCACGCAGACACGATTAGAGGATGCAGCGCCAGAAACAGAGAAAAACACAGTGGTGGAA

GGCGGAGAGGTGCGCTTCAACGGCAAAGGGAAAAAGATTAGAAAACCAAGGACTATATAT

TCGAGTTTACAA

>dlx1a_Gnaper

GGGGAACCGCCAGAAGAGATGACAATGACTACGATTCCAGAAAGTCTTAATAGCCCTGCC

TCAGGAAAAACCGTTTTCATGGAGTTTGGACCCCCGAGTCAACAAATGTCGCCTTCCTCC

ATGTCTCACGGACACTATCCCATGCACTGTTTACATTCTGCAGGTCACACACAGCACGAC

AGCTACAGTCCAGCCTCGTCGTTCCCCAGATCCTTGGGTTATCCATACGTAAACTCCGTC

GGCAGCCATTCCACCAGTCCATATCTCAGCACAGTACAGACTTACCAAAACAGCTCGGGA

CTCACGCAGACACGATTAGAGGATGCAGCGCCAGAAACAGAGAAAAACACAGTGGTGGAA

GGCGGAGAGGTGCGCTTCAACGGCAAAGGGAAAAAGATTAGAAAACCAAGGACTATATAT

TCGAGTTTACAA

>dlx1a_Oretan

GGGGAACCGCCAGAAGAGATGACAATGACTACGATTCCAGAAAGTCTTAATAGCCCTGCC

TCAGGAAAAACCGTTTTCATGGAGTTTGGACCCCCGAGTCAACAAATGTCGCCTTCCTCC

ATGTCTCACGGACACTATCCCATGCACTGTTTACATTCTGCAGGTCACACACAGCACGAC

AGCTACAGTCCAGCCTCGTCGTTCCCCAGATCCTTGGGTTATCCATACGTAAACTCCGTC

GGCAGCCATTCCACCAGTCCATATCTCAGCACAGTACAGACTTACCAAAACAGCTCGGGA

CTCACGCAGACACGATTAGAGGATGCAGCGCCAGAAACAGAGAAAAACACAGTGGTGGAA

GGCGGAGAGGTGCGTTTCAACGGCAAAGGGAAAAAGATTAGAAAACCAAGGACTATATAT

TCGAGTTTACAA

>dlx1a_Petfam

GGGGAACCGCCAGAAGAGATGACAATGACTACGATTCCAGAAAGTCTTAATAGCCCTGCC

TCAGGAAAAACCGTTTTCATGGAGTTTGGACCCCCGAGTCAACAAATGTCGCCTTCCTCC

ATGTCTCACGGACACTATCCCATGCACTGTTTACATTCTGCAGGTCACACACAGCACGAC

AGCTACAGTCCAGCCTCGTCGTTCCCCAGATCCTTGGGTTATCCATACGTAAACTCCGTC

GGCAGCCATTCCACCAGTCCATATCTCAGCACAGTACAGACTTACCAAAACAGCTCGGGA

CTCACGCAGACACGATTAGAGGATGCAGCGCCAGAAACAGAGAAAAACACAGTGGTGGAA

GGCGGAGAGGTGCGCTTCAACGGCAAAGGGAAAAAGATTAGAAAACCAAGGACTATATAT

TCGAGTTTACAA

>dlx1a_Psecur

GGGGAACCGCCAGAAGAGATGACAATGACTACGATTCCAGAAAGTCTTAATAGCCCTGCC

TCAGGAAAAACCGTTTTCATGGAGTTTGGACCCCCGAGTCAACAAATGTCGCCTTCCTCC

ATGTCTCACGGACACTATCCCATGCACTGTTTACATTCTGCAGGTCACACACAGCACGAC

AGCTACAGTCCAGCCTCGTCGTTCCCCAGATCCTTGGGTTATCCATACGTAAACTCCGTC

GGCAGCCATTCCACCAGTCCATATCTCAGCACAGTACAGACTTACCAAAACAGCTCGGGA

CTCACGCAGACACGATTAGAGGATGCAGCGCCAGAAACAGAGAAAAACACAGTGGTGGAA

GGCGGAGAGGTGCGCTTCAACGGCAAAGGGAAAAAGATTAGAAAACCAAGGACTATATAT

TCGAGTTTACAA

>dlx1a_Boumic

GGGGAACCGCCAGAAGAGATGACAATGACTACGATTCCAGAAAGTCTTAATAGCCCTGCC

TCAGGAAAAACCGTTTTCATGGAGTTTGGACCCCCGAGTCAACAAATGTCGCCTTCCTCC

ATGTCTCACGGACACTATCCCATGCACTGTTTACATTCTGCAGGTCACACACAGCACGAC

AGCTACAGTCCAGCCTCGTCGTTCCCCAGATCCTTGGGTTATCCATACGTAAACTCCGTC

GGCAGCCATTCCACCAGTCCATATCTCAGCACAGTACAGACTTACCAAAACAGCTCGGGA

CTCACGCAGACACGATTAGAGGATGCAGCGCCAGAAACAGAGAAAAACACAGTGGTGGAA

GGCGGAGAGGTGCGCTTCAACGGCAAAGGGAAAAAGATTAGAAAACCAAGGACTATATAT

TCGAGTTTACAA

>dlx1a_Cypfro

GGGGAACCGCCAGAAGAGATGACAATGACTACGATTCCAGAAAGTCTTAATAGCCCTGCC

TCAGGAAAAACCGTTTTCATGGAGTTTGGACCCCCGAGTCAACAAATGTCGCCTTCCTCC

ATGTCTCACGGACACTATCCCATGCACTGTTTACATTCTGCAGGTCACACACAGCACGAC

AGCTACAGTCCAGCCTCGTCGTTCCCCAGATCCTTGGGTTATCCATACGTAAACTCCGTC

GGCAGCCATTCCACCAGTCCATATCTCAGCACAGTACAGACTTACCAAAACAGCTCGGGA

CTCACGCAGACACGATTAGAGGATGCAGCGCCAGAAACAGAGAAAAACACAGTGGTGGAA

GGCGGAGAGGTGCGCTTCAACGGCAAAGGGAAAAAGATTAGAAAACCAAGGACTATATAT

TCGAGTTTACAA

>dlx1a_Cyplep

GGGGAACCGCCAGAAGAGATGACAATGACTACGATTCCAGAAAGTCTTAATAGCCCTGCC

TCAGGAAAAACCGTTTTCATGGAGTTTGGACCCCCGAGTCAACAAATGTCGCCTTCCTCC

ATGTCTCACGGACACTATCCCATGCACTGTTTACATTCTGCAGGTCACACACAGCACGAC

AGCTACAGTCCAGCCTCGTCGTTCCCCAGATCCTTGGGTTATCCATACGTAAACTCCGTC

GGCAGCCATTCCACCAGTCCATATCTCAGCACAGTACAGACTTACCAAAACAGCTCGGGA

CTCACGCAGACACGATTAGAGGATGCAGCGCCAGAAACAGAGAAAAACACAGTGGTGGAA

GGCGGAGAGGTGCGCTTCAATGGCAAAGGGAAAAAGATTAGAAAACCAAGGACTATATAT

TCGAGTTTACAA

>dlx1a_Calmac

GGGGAACCGCCAGAAGAGATGACAATGACTACGATTCCAGAAAGTCTTAATAGCCCTGCC

TCAGGAAAAACCGTTTTCATGGAGTTTGGACCCCCGAGTCAACAAATGTCGCCTTCCTCC

ATGTCTCACGGACACTATCCCATGCACTGTTTACATTCTGCAGGTCACACACAGCACGAC

AGCTACAGTCCAGCCTCGTCGTTCCCCAGATCCTTGGGTTATCCATACGTAAACTCCGTC

GGCAGCCATTCCACCAGTCCATATCTCAGCACAGTACAGACTTACCAAAACAGCTCGGGA

CTCACGCAGACACGATTAGAGGATGCAGCGCCAGAAACAGAGAAAAACACAGTGGTGGAA

GGCGGAGAGGTGCGCTTCAACGGCAAAGGGAAAAAGATTAGAAAACCAAGGACTATATAT

TCGAGTTTACAA

>dlx1a_Cyafur

GGGGAACCGCCAGAAGAGATGACAATGACTACGATTCCAGAAAGTCTTAATAGCCCTGCC

TCAGGAAAAACCGTTTTCATGGAGTTTGGACCCCCGAGTCAACAAATGTCGCCTTCCTCC

ATGTCTCACGGACACTATCCCATGCACTGTTTACATTCTGCAGGTCACACACAGCACGAC

AGCTACAGTCCAGCCTCGTCGTTCCCCAGATCCTTGGGTTATCCATACGTAAACTCCGTC

GGCAGCCATTCCACCAGTCCATATCTCAGCACAGTACAGACTTACCAAAACAGCTCGGGA

CTCACGCAGACACGATTAGAGGATGCAGCGCCAGAAACAGAGAAAAACACAGTGGTGGAA

GGCGGAGAGGTGCGCTTCAACGGCAAAGGGAAAAAGATTAGAAAACCAAGGACTATATAT

TCGAGTTTACAA

>dlx1a_Astbur

GGGGAACCGCCAGAAGAGATGACAATGACTACGATTCCAGAAAGTCTTAATAGCCCTGCC

TCAGGAAAAACCGTTTTCATGGAGTTTGGACCCCCGAGTCAACAAATGTCGCCTTCCTCC

ATGTCTCACGGACACTATCCCATGCACTGTTTACATTCTGCAGGTCACACACAGCACGAC

AGCTACAGTCCAGCCTCGTCGTTCCCCAGATCCTTGGGTTATCCATACGTAAACTCCGTC

GGCAGCCATTCCACCAGTCCATATCTCAGCACAGTACAGACTTACCAAAACAGCTCGGGA

CTCACGCAGACACGATTAGAAGATGCAGCGCCAGAAACAGAGAAAAACACAGTGGTGGAA

GGCGGAGAGGTGCGCTTCAACGGCAAAGGGAAAAAGATTAGAAAACCAAGGACTATATAT

TCGAGTTTACAA

>dlx1a_Ctehor

GGGGAACCGCCAGAAGAGATGACAATGACTACGATTCCAGAAAGTCTTAATAGCCCTGCC

TCAGGAAAAACCGTTTTCATGGAGTTTGGACCCCCGAGTCAACAAATGTCGCCTTCCTCC

ATGTCTCACGGACACTATCCCATGCACTGTTTACATTCTGCAGGTCACACACAGCACGAC

AGCTACAGTCCAGCCTCGTCGTTCCCCAGATCCTTGGGTTATCCATACGTAAACTCCGTC

GGCAGCCATTCCACCAGTCCATATCTCAGCACAGTACAGACTTACCAAAACAGCTCGGGA

CTCACGCAGACACGATTAGAGGATGCAGCGCCAGAAACAGAGAAAAACACAGTGGTGGAA

GGCGGAGAGGTGCGCTTCAACGGCAAAGGGAAAAAGATTAGAAAACCAAGGACTATATAT

TCGAGTTTACAA

>dlx1a_Neofur

GGGGAACCGCCAGAAGAGATGACAATGACTACGATTCCAGAAAGTCTTAATAGCCCTGCC

TCAGGAAAAACCGTTTTCATGGAGTTTGGACCCCCGAGTCAACAAATGTCGCCTTCCTCC

ATGTCTCACGGACACTATCCCATGCACTGTTTACATTCTGCAGGTCACACACAGCACGAC

AGCTACAGTCCAGCCTCGTCGTTCCCCAGATCCTTGGGTTATCCATACGTAAACTCCGTC

GGCAGCCATTCCACCAGTCCATATCTCAGCACAGTACAGACTTACCAAAACAGCTCGGGA

CTCACGCAGACACGATTAGAGGATGCAGCGCCAGAAACAGAGAAAAACACAGTGGTGGAA

GGCGGAGAGGTGCGCTTCAACGGCAAAGGGAAAAAGATTAGAAAACCAAGGACTATATAT

TCGAGTTTACAA

Dlx2a input file:

>dlx2a_Limsta

CAGATTACCTCAAGCAATTACCACAGCTTGCACAAATCGCAGGAATCCCCGACTCTGCCG

GTTTCCACGGCGACGGACAGCAGCTATTACAACGGCCAGCAGCCTGCGCACTGCGCCGGG

TCACCGTTTGGACAACTGAGCACTTACCAGTACCACAGCAGCGCCACGAGTTCTGTGCCA

TATAACGCAAAGTCATACGACCTCGGTTTCAACTCATCGTATGGTACATACGGTTCTTAT

GGCTCCAACTCATCGCCAACTCCCGCAGACACAGAGAAAGATGAGAGCGAGCCAGAAATC

CGGATGGTTAATGGAAAACCAAAGAAGGTCAGGAAACCTCGAACCATTTACTCCAGCTTC

CAACTGGCTGCACTTCAACGGCGGTTTCAAAAGACTCAGTATTTGGCTCTACCAGAACGG

GCCGAGCTGGCAGCGTCGCTGGGCCTTACGCAAACACAAGTTAAAATCTGGTTCCAAAAC

CGCCGCTCCAAGTTCAAGAAGCTGTGGAAAAGTGGAGAAATCCCCCCAGAACAACATGTT

GCTTCCAGTGAATCTCCCCCGTGCACGTCTCCACCAACTACCGCCTGGGACTTTCCACAG

ACTCAAAGAATGAACAATGTCAGCTCTAGTTTACCTCAGAGCAGCAGCCCTCCAAACACG

ACTGCGCCTTCGTCGTTTTTGGCAAACTACTCCTGGTACTCAACTACGAACTCTGCCACG

CATCTGCAGCCTCCTCTGGTTCAGCATCACCACAACTCCGCCATA

>dlx2a_Loblab

CAGATTACCTCAAGCAATTACCACAGCTTGCACAAATCGCAGGAGTCCCCGACTCTGCCG

GTTTCCACGGCGACGGACAGCAGCTATTACAACGGCCAGCAGCCTGGGCACTGCGCCGGG

TCACCGTTTGGACAACTGGGCTCTTACCAGTACCACAGCAGCGCCACGAGTTCTGTGCCA

TATAACGCAAAGTCATACGACCTCGGTTTCAACTCATCGTATGGTACATACGGTTCTTAT

GGCTCCAACTCATCGCCAACTCCCGCAGACACAGAGAAAGATGAGAGCGAGCCAGAAATC

CGGATGGTTAATGGAAAACCAAAGAAGGTCAGGAAACCTCGAACCATTTACTCCAGCTTC

CAACTGGCTGCGCTTCAACGGAGGTTTCAAAAGACTCAGTATTTGGCTCTACCAGAACGG

GCCGAGCTGGCAGCGTCGCTGGGCCTTACGCAAACACAGGTTAAAATCTGGTTCCAAAAC

CGCCGCTCCAAGTTCAAGAAGCTGTGGAAAAGTGGAGAAATCCCCCCAGAACAACATGTT

GCTTCCAGTGAATCTCCCCCGTGCACGTCTCCACCAACTACCGCCTGGGACTTTCCACAG

ACTCAAAGAATGAACAATGTCAGCTCTAGTTTACCTCAGAGCAGCAGCCCTCCAAACACG

ACTGCGCCTTCGTCGTTTTTGGCAAACTACTCCTGGTACTCAACTACGAACTCTGCCACG

CATCTGCAGCCTCCTCTGGTTCAGCATCACCACAACTCCGCCATA

>dlx2a_Tromoo

CAGATTACCTCAAGCAATTACCACAGCTTGCACAAATCGCAGGAGTCCCCGACTCTGCCG

GTTTCCACGGCGACGGACAGCAGCTATTACAACGGCCAGCAGCCTGGGCACTGCGCCGGG

TCACCGTTTGGACAACTGGGCTCTTACCAGTACCACAGCAGCGCCACGAGTTCTGTGCCA

TATAACGCAAAGTCATACGACCTCGGTTTCAACTCATCGTATGGTACATACGGTTCTTAT

GGCTCCAACTCATCGCCAACTCCCGCAGACACAGAGAAAGATGAGAGCGAGCCAGAAATC

CGGATGGTTAATGGAAAACCAAAGAAGGTCAGGAAACCTCGAACCATTTACTCCAGCTTC

CAACTGGCTGCGCTTCAACGGAGGTTTCAAAAGACTCAGTATTTGGCTCTACCAGAACGG

GCCGAGCTGGCAGCGTCGCTGGGCCTTACGCAAACACAGGTTAAAATCTGGTTCCAAAAC

CGCCGCTCCAAGTTCAAGAAGCTGTGGAAAAGTGGAGAAATCCCCCCAGAACAACATGTT

GCTTCCAGTGAATCTCCCCCGTGCACGTCTCCACCAACTACCGCCTGGGACTTTCCACAG

ACTCAAAGAATGAACAATGTCAGCTCTAGTTTACCTCAGAGCAGCAGCCCTCCAAACACG

ACTGCGCCTTCGTCGTTTTTGGCAAACTACTCCTGGTACTCAACTACGAACTCTGCCACG

CATCTGCAGCCTCCTCTGGTTCAGCATCACCACAACTCCGCCATA

>dlx2a_Tylpol

CAGATTACCTCAAGCAATTACCACAGCTTGCACAAATCGCAGGAGTCCCCGACTCTGCCG

GTTTCCACGGCGACGGACAGCAGCTATTACAACGGCCAGCAGCCTGGGCACTGCGCCGGG

TCACCGTTTGGACAACTGGGCTCTTACCAGTACCACAGCAGCGCCACGAGTTCTGTGCCA

TATAACGCAAAGTCATACGACCTCGGTTTCAACTCATCGTATGGTACATACGGTTCTTAT

GGCTCCAACTCATCGCCAACTCCCGCAGACACAGAGAAAGATGAGAGCGAGCCAGAAATC

CGGATGGTTAATGGAAAACCAAAGAAGGTCAGGAAACCTCGAACCATTTACTCCAGCTTC

CAACTGGGTGCGCTTCAACGGAGGTTTCAAAAGACTCAGTATTTGGCTCTACCAGAACGG

GCCGAGCTGGCAGCGTCGCTGGGCCTTACGCAAACACAGGTTAAAATCTGGTTCCAAAAC

CGCCGCTCCAAGTTCAAGAAGCTGTGGAAAAGTGGAGAAATCCCCCCAGAACAACATGTT

GCTTCCAGTGAATCTCCCCCGTGCACGTCTCCACCAACTACCGCCTGGGACTTTCCACAG

ACTCAAAGAATGAACAATGTCAGCTCTAGTTTACCTCAGAGCAGCAGCCCTCCAAACACG

ACTGCGCCTTCGTCGTTTTTGGCAAACTACTCCTGGTACTCAACTACGAACTCTGCCACG

CATCTGCAGCCTCCTCTGGTTCAGCATCACCACAACTCCGCCATA

>dlx2a_Altfas

CAGATTACCTCAAGCAATTACCACAGCTTGCACAAATCGCAGGAGTCCCCGACTCTGCCG

GTTTCCACGGCGACGGACAGCAGCTATTACAACGGCCAGCAGCCTGCGCACTGCGCCGGG

TCACCGTTTGGACAACTGGGCACTTACCAGTACCACAGCAGCGCCACGAGTTCTGTGCCA

TATAACGCAAAGTCATACGACCTCGGTTTCAACTCATCGTATGGTACATACGGTTCTTAT

GGCTCCAACTCATCGCCAACTCCCGCAGACACAGAGAAAGATGAGAGCGAGCCAGAAATC

CGGATGGTTAATGGAAAACCAAAGAAGGTCAGGAAACCTCGAACCATTTACTCCAGCTTC

CAACTGGCTGCACTTCAACGGAGGTTTCAAAAGACTCAGTATTTGGCTCTACCAGAACGG

GCCGAGCTGGCAGCGTCGCTGGGCCTTACGCAAACACAGGTTAAAATCTGGTTCCAAAAC

CGCCGCTCCAAGTTCAAGAAGCTGTGGAAAAGTGGAGAAATCCCCCCAGAACAACATGTT

GCTTCCAGTGAATCTCCCCCGTGCACGTCTCCACCAACTACCACCTGGGACTTTCCACAG

ACTCAAAGAATGAACAATGTCAGCTCTAGTTTACCTCAGAGCAGCAGCCCTCCAAACACG

ACTGCGCCTTCGTCGTTTTTGGCAAACTACTCCTGGTACTCAACTACGAACTCTGCCACG

CATCTGCAGCCTCCTCTGGTTCAGCATCACCACAACTCCGCCATA

>dlx2a_Lepelo

CAGATTACCTCAAGCAATTACCACAGCTTGCACAAATCGCAGGAGTCCCCGACTCTGCCG

GTTTCCACGGCGACGGACAGCAGCTATTACAACGGCCAGCAGCCTGCGCACTGCGCCGGG

TCACCGTTTGGACAACTGGGCACTTACCAGTACCACAGCAGCGCCACGAGTTCTGTGCCA

TATAACGCAAAGTCATACGACCTCGGTTTCAACTCATCGTATGGTACATACGGTTCTTAT

GGCTCCAACTCATCGCCAACTCCCGCAG--------------------------------

------------------------------------------------------------

------------------------------------------------------------

---------------------------------ACACAGGTTAAAATCTGGTTCCAAAAC

CGCCGCTCCAAGTTCAAGAAGCTGTGGAAAAGTGGAGAAATCCCCCCAGAACAACATGTT

GCTTCCAGTGAATCTCCCCCGTGCACGTCTCCACCAACTACCACCTGGGACTTTCCACAG

ACTCAAAGAATGAACAATGTCAGCTCTAGTTTACCTCAGAGCAGCAGCCCTCCAAACACG

ACTGCGCCTTCGTCGTTTTTGGCAAACTACTCCTGGTACTCAACTACGAACTCTGCCACG

CATCTGCAGCCTCCTCTGGTTCAGCATCACCACAACTCCGCCATA

>dlx2a_Varmoo

CAGATTACCTCAAGCAATTACCACAGCTTGCACAAATCGCAGGAGTCCCCGACTCTGCCG

GTTTCCACGGCGACGGACAGCAGCTATTACAACGGCCAGCAGCCTGCGCACTGCGCCGGG

TCACCGTTTGGACAACTGGGCACTTACCAGTACCACAGCAGCGCCACGAGTTCTGTGCCA

TATAACGCAAAGTCATACGACCTCGGTTTCAACTCATCGTATGGTACATACGGTTCTTAT

GGCTCCAACTCATCGCCAACTCCCGCAGACACAGAGAAAGATGAGAGCGAGCCAGAAATC

CGGATGGTTAATGGAAAACCAAAGAAGGTCAGGAAACCTCGAACCATTTACTCCAGCTTC

CAACTGGCTGCACTTCAACGGAGGTTTCAAAAGACTCAGTATTTGGCTCTACCAGAACGG

GCCGAGCTGGCAGCGTCGCTGGGCCTTACGCAAACACAGGTTAAAATCTGGTTCCAAAAC

CGCCGCTCCAAGTTCAAGAAGCTGTGGAAAAGTGGAGAAATCCCCCCAGAACAACATGTT

GCTTCCAGTGAATCTCCCCCGTGCACGTCTCCACCAACTACCGCCTGGGACTTTCCACAG

ACTCAAAGAATGAACAATGTCAGCTCTAGTTTACCTCAGAGCAGCAGCCCTCCAAACACG

ACTGCGCCTTCGTCGTTTTTGGCAAACTACTCCTGGTACTCAACTACGAACTCTGCCACG

CATCTGCAGCCTCCTCTGGTTCAGCATCACCACAACTCCGTCCTA

>dlx2a_Psecur

CAGATTACCTCAAGCAATTACCACAGCTTGCACAAATCGCAGGAGTCCCCGACTCTGCCG

GTTTCCACGGCGACGGACAGCAGCTATTACAACGGCCAGCAGCCTGGGCACTGCGCCGGG

TCACCGTTTGGACAACTGGGCTCTTACCAGTACCACAGCAGCGCCACGAGTTCTGTGCCA

TATAACGCAAAGTCATACGACCTCGGTTTCAACTCATCGTATGGTACATACGGTTCTTAT

GGCTCCAACTCATCGCCAACTCCCGCAGACACAGAGAAAGATGAGAGCGAGCCAGAAATC

CGGATGGTTAATGGAAAACCAAAGAAGGTCAGGAAACCTCGAACCATTTACTCCAGCTTC

CAACTGGCTGCGCTTCAACGGAGGTTTCAAAAGACTCAGTATTTGGCTCTACCAGAACGG

GCCGAGCTGGCAGCGTCGCTGGGCCTTACGCAAACACAGGTTAAAATCTGGTTCCAAAAC

CGCCGCTCCAAGTTCAAGAAGCTGTGGAAAAGTGGAGAAATCCCCCCAGAACAACATGTT

GCTTCCAGTGAATCTCCCCCGTGCACGTCTCCACCAACTACCGCCTGGGACTTTCCACAG

ACTCAAAGAATGAACAATGTCAGCTCTAGTTTACCTCAGAGCAGCAGCCCTCCAAACACG

ACTGCGCCTTCGTCGTTTTTGGCAAACTACTCCTGGTACTCAACTACGAACTCTGCCACG

CATCTGCAGCCTCCTCTGGTTCAGCATCACCACAACTCCGCCATA

>dlx2a_Cypfro

CAGATTACCTCAAGCAATTACCACAGCTTGCACAAATCGCAGGAGTCCCCGACTCTGCCG

GTTTCCACGGCGACGGACAGCAGCTATTACAACGGCCAGCAGCCTGGGCACTGCGCCGGG

TCACCGTTTGGACAACTGGGCACTTACCAGTACCACAGCAGCGCCACGAGTTCTGTGCCA

TATAACGCAAAGTCATACGACCTCGGTTTCAACTCATCGTATGGTACATACGGTTCTTAT

GGCTCCAACTCATCGCCAACTCCCGCAGACACAGAGAAAGATGAGAGCGAGCCAGAAATC

CGGATGGTTAATGGAAAACCAAAGAAGGTCAGGAAACCTCGAACCATTTACTCCAGCTTC

CAACTGGCTGCACTTCAACGGAGGTTTCAAAAGACTCAGTATTTGGCTCTACCAGAACGG

GCCGAGCTGGCAGCGTCGCTGGGCCTTACGCAAACACAGGTTAAAATCTGGTTCCAAAAC

CGCCGCTCCAAGTTCAAGAAGCTGTGGAAAAGTGGAGAAATCCCCCCAGAACAACATGTT

GCTTCCAGTGAATCTCCCCCGTGCACGTCTCCACCAACTACCGCCTGGGACTTTCCACAG

ACTCAAAGAATGAACAATGTCAGCTCTAGTTTACCTCAGAGCAGCAGCCCTCCAAACACG

ACTGCGCCTTCGTCGTTTTTGGCAAACTACTCCTGGTACTCAACTACGAACTCTGCCACG

CATCTGCAGCCTCCTCTGGTTCAGCATCACCACAACTCCGCCATA

>dlx2a_Cyplep

CAGATTACCTCAAGCAATTACCACAGCTTGCACAAATCGCAGGAGTCCCCGACTCTGCCG

GTTTCCACGGCGACGGACAGCAGCTATTACAACGGCCAGCAGCCTGGGCACTGCGCCGGG

TCACCGTTTGGACAACTGGGCACTTACCAGTACCACAGCAGCGCCGCGAGTTCTGTGCCA

TATAACGCAAAGTCATACGACCTCGGTTTCAACTCATCGTATGGTACATACGCTTCTTAT

GGCTCCAACTCATCGCCAACTCCCGCAGACACAGAGAAAGATGAGAGCGAGCCAGAAATC

CGGATGGTTAATGGAAAACCAAAGAAGGTCAGGAAACCTCGAACCATTTACTCCAGCTTC

CAACTGGCTGCACTTCAACGGAGGTTTCAAAAGACTCAGTATTTGGCTCTACCAGAACGG

GCCGAGCTGGCAGCGTCGCTGGGCCTTACGCAAACACAGGTTAAAATCTGGTTCCAAAAC

CGCCGCTCCAAGTTCAAGAAGCTGTGGAAAAGTGGAGAAATCCCCCCAGAACAACATGTT

GCTTCCAGTGAATCTCCCCCGTGCACGTCTCCACCAACTACCGCCTGGGACTTTCCACAG

ACTCAAAGAATGAACAATGTCAGCTCTAGTTTACCTCAGAGCAGCAGCCCTCCAAACACG

ACTGCGCCTTCGTCGTTTTTGGCAAACTACTCCTGGTACTCAACTACGAACTCTGCCACG

CATCTGCAGCCTCCTCTGGTTCAGCATCACCACAACTCCGCCATA

>dlx2a_Calmac

CAGATTACCTCAAGCAATTACCACAGCTTGCACAAATCGCAGGAGTCCCCGACTCTGCCG

GTTTCCACGGCGACGGACAGCAGCTATTACAACGGCCAGCAGCCTGGGCACTGCGCCGGG

TCACCGTTTGGACAACTGGGCACTTACCAGTACCACAGCAGCGCCACGAGTTCTGTGCCA

TATAACGCAAAGTCATACGACCTCGGTTTCAACTCAACGTATGGTACATACGGTTCTTAT

GGCTCCAACTCATCGCCAACTCCCGCAGACACAGAGAAAGATGAGAGCGAGCCAGAAATC

CGGATGGTTAATGGAAAACCAAAGAAGGTCAGGAAACCTCGAACCATTTACTCCAGCTTC

CAACTGGCTGCACTTCAACGGAGGTTTCAAAAGACTCAGTATTTGGCTCTACCAGAACGG

GCCGAGCTGGCAGCGTCGCTGGGCCTTACGCAAACACAGGTTAAAATCTGGTTCCAAAAC

CGCCGCTCCAAGTTCAAGAAGCTGTGGAAAAGTGGAGAGATCCCCCCAGAACAACATGTT

GCTTCCAGTGAATCTCCCCCGTGCACGTCTCCACCAACTACCGCCTGGGACTTTCCACAG

ACTCAAAGAATGAACAATGTCAGTTCTAGTTTACCTCAGAGCAACAGCCCTCCAAACACG

ACTGCGCCTTCGTCGTTTTTGGCAAACTACTCCTGGTACTCAACCACGAACTCTGCCACG

CATCTGCAGCCTCCTCTGGTTCAGCATCACCACAACTCCGCCATA

>dlx2a_Cyafur

CAGATTACCTCAAGCAATTACCACAGCTTGCACAAATCGCAGGAGTCCCCGACTCTGCCG

GTTTCCACGGCGACGGACAGCAGCTATTACAACGGCCAGCAGCCTGGGCACTGCGCCGGG

TCACCGTTTGGACAACTGGGCACTTACCAGTACCACAGCAGCGCCACGAGTTCTGTGCCA

TATAACGCAAAGTCATACGACCTCGGTTTCAACTCATCGTATGGTACATACGGTTCTTAT

GGCTCCAACTCATCGCCAACTCCCGCAGACACAGAGAAAGATGAGAGCGAGCCAGAAATC

CGGATGGTTAATGGAAAACCAAAGAAGGTCAGGAAACCTCGAACCATTTACTCCAGCTTC

CAACTGGCTGCACTTCAACGGAGGTTTCAAAAGACTCAGTATTTGGCTCTACCAGAACGG

GCCGAGCTGGCAGCGTCGCTGGGCCTTACGCAAACACAGGTTAAAATCTGGTTCCAAAAC

CGCCGCTCCAAGTTCAAGAAGCTGTGGAAAAGTGGAGAAATCCCCCCAGAACAACATGTT

GCTTCCAGTGAATCTCCCCCGTGCACGTCTCCACCAACTACCGCCTGGGACTTTCCACAG

ACTCAAAGAATGAACAATGTCAGTTCTAGTTTACCTCAGAGCAACAGCCCTCCAAACACG

ACTGCGCCTTCGTCGTTTTTGGCAAACTACTCCTGGTACTCAACTACGAACTCTGCCACG

CATCTGCAGCCTCCTCTGGTTCAGCATCACCACAACTCCGCCATA

>dlx2a_Astbur

CAGATTACCTCAAGCAATTACCACAGCTTGCACAAATCGCAGGAGTCCCCGACTCTGCCG

GTTTCCACGGCGACGGACAGCAGCTATTACAACGGCCAGCAGCCTGGGCACTGCGCCGGG

TCACCGTTTGGACAACTGGGCACTTACCAGTACCACAGCAGCGCCACGAGTTCTGTGCCA

TATAACGCAAAGTCATACGACCTCGGTTTCAACTCATCGTATGGTACATACGGTTCTTAT

GGCTCCAACTCATCGCCAACTCCCGCAGACACAGAGAAAGATGAGAGCGAGCCAGAAATC

CGGATGGTTAATGGAAAACCAAAGAAGGTCAGGAAACCTCGAACCATTTACTCCAGCTTC

CAACTGGCTGCGCTTCAACGGAGGTTTCAAAAGACTCAGTATTTGGCTCTACCAGAACGG

GCCGAGCTGGCAGCGTCGCTGGGCCTTACGCAAACACAGGTTAAAATCTGGTTCCAAAAC

CGCCGCTCCAAGTTCAAGAAGCTGTGGAAAAGTGGAGAAATCCCCCCAGAACAACATGTT

GCTTCCAGTGAATCTCCCCCGTGCACGTCTCCACCAACTACCGCCTGGGACTTTCCACAG

ACTCAAAGAATGAACAATGTCAGCTCTAGTTTACCTCAGAGCAGCAGCCCTCCAAACACG

ACTGCGCCTTCGTCGTTTTTGGCAAACTACTCCTGGTACTCAACTACGAACTCTACCACG

CATCTGCAGCCTCCTCTGGTTCAGCATCACCACAACTCCGCCATA

>dlx2a_Ctehor

CAGATTACCTCAAGCAATTACCACAGCTTGCACAAATCGCAGGAGTCCCCGACTCTGCCG

GTTTCCACGGCGACGGACAGCAGCTATTACAACGGCCAGCAGCCTGGGCACTGCGCCGGG

TCACCGTTTGGACAACTGGGCTCTTACCAGTACCACAGCAGCGCCACGAGTTCTGTGCCA

TATAACGCAAAGTCATACGACCTCGGTTTCAACTCATCGTATGGTACATACGGTTCTTAT

GGCTCCAACTCATCGCCAACTCCCGCAGACACAGAGAAAGATGAGAGCGAGCCAGAAATC

CGGATGGTTAATGGAAAACCAAAGAAGGTCAGGAAACCTCGAACCATTTACTCCAGCTTC

CAACTGGCTGCGCTTCAACGGAGGTTTCAAAAGACTCAGTATTTGGCTCTACCAGAACGG

GCCGAGCTGGCAGCGTCGCTGGGCCTTACGCAAACACAGGTTAAAATCTGGTTCCAAAAC

CGCCGCTCCAAGTTCAAGAAGCTGTGGAAAAGTGGAGAAATCCCCCCAGAACAACATGTT

GCTTCCAGTGAATCTCCCCCGTGCACGTCTCCACCAACTACCGCCTGGGACTTTCCACAG

ACTCAAAGAATGAACAATGTCAGCTCTAGTTTACCTCAGAGCAGCAGCCCTCCAAACACG

ACTGCGCCTTCGTCGTTTTTGGCAAACTACTCCTGGTACTCAACTACGAACTCTGCCACG

CATCTGCAGCCTCCTCTGGTTCAGCATCACCACAACTCCGCCATA

>dlx2a_Batgra

CAGATTACCTCAAGCAATTACCACAGCTTGCACAAATCGCAGGAGTCCCCGACTCTGCCG

GTTTCCACGGCGACGGACAGCAGCTATTACAACGGCCAGCAGCCTGGGCACTGCGCCGGG

TCACCGTTTGGACAACTGGGCACTTACCAGTACCACAGCAGCGCCACGAGTTCTGTGCCA

TATAACGCAAAGTCATACGACCTCGGTTTCAACTCATCGTATGGTACATACGGTTCTTAT

GGCTCCAACTCATCGCCAACTCCCGCAGACACAGAGAAAGATGAGAGCGAGCCAGAAATC

CGGATGGTTAATGGAAAACCAAAGAAGGTCAGGAAACCTCGAACCATTTACTCCAGCTTC

CAACTGGCTGCACTTCAACGGAGGTTTCAAAAGACTCAGTATTTGGCTCTACCAGAACGG

GCCGAGCTGGCAGCGTCGCTGGGCCTTACGCAAACACAGGTTAAAATCTGGTTCCAAAAC

CGCCGCTCCAAGTTCAAGAAGCTGTGGAAAAGTGGAGAAATCCCCCCAGAACAACATGTT

GCTTCCAGTGAATCTCCCCCGTGCACGTCTCCACCAACCACCGCCTGGGACTTTCCACAG

ACTCAAAGAATGAACAATGTCAGCTCTAGTTTACCTCAGAGCAGCAGCCCTCCAAACACG

ACTGCGCCTTCGTCGTTTTTGGCAAACTACTCCTGGTACTCAACTACGAACTCTGC----

---------------------------------------------

>dlx2a_Boumic

CAGATTACCTCAAGCAATTACCACAGCTTGCACAAATCGCAGGAGTCCCCGACTCTGCCG

GTTTCCACGGCGACGGACAGCAGCTATTACAACGGCCAGCAGCCTGGGCACTGCGCCGGG

TCACCGTTTGGACAACTGGGCACTTACCAGTACCACAGCAGCGCCACGAGTTCTGTGCCA

TATAACGCAAAGTCATACGACCTCGGTTTCAACTCATCGTATGGTACATACGGTTCTTAT

GGCTCCAACTCATCGCCAACTCCCGCAGACACAGAGAAAGATGAGAGCGAGCCAGAAATC

CGGATGGTTAATGGAAAACCAAAGAAGGTCAGGAAACCTCGAACCATTTACTCCAGCTTC

CAACTGGCTGCACTTCAACGGAGGTTTCAAAAGACTCAGTATTTGGCTCTACCAGAACGG

GCCGAGCTGGCAGCGTCGCTGGGCCTTACGCAAACACAGGTTAAAATCTGGTTCCAAAAC

CGCCGCTCCAAGTTCAAGAAGCTGTGGAAAAGTGGAGAAATCCCCCCAGAACAACATGTT

GCTTCCAGTGAATCTCCCCCGTGCACGTCTCCACCAACTACCGCCTGGGACTTTCCACAG

ACTCAAAGAATGAACAATGTCAGCTCTAGTTTACCTCAGAGCAGCAGCCCTCCAAACACG

ACTGCGCCTTCGTCGTTTTTGGCAAACTACTCCTGGTACTCAACTACGAACTCTGCCACG

CATCT----------------------------------------

>dlx2a_Petfam

CAGATTACCTCAAGCAATTACCACAGCTTGCACAAATCGCAGGAGTCCCCGACTCTGCCG

GTTTCCACGGCGACGGACAGCAGCTATTACAACGGCCAGCAGCCTGGGCACTGCGCCGGG

TCACCGTTTGGACAACTGGGCTCTTACCAGTACCACAGCAGCGCCACGAGTTCTGTGCCA

TATAACGCAAAGTCATACGACCTCGGTTTCAACTCATCGTATGGTACATACGGTTCTTAT

GGCTCCAACTCATCGCCAACTCCCGCAGACACAGAGAAAGATGAGAGCGAGCCAGAAATC

CGGATGGTTAATGGAAAACCAAAGAAGGTCAGGAAACCTCGAACCATTTACTCCAGCTTC

CAACTGGCTGCGCTTCAACGGAGGTTTCAAAAGACTCAGTATTTGGCTCTACCAGAACGG

GCCGAGCTGGCAGCGTCGCTGGGCCTTACGCAAACACAGGTTAAAATCTGGTTCCAAAAC

CGCCGCTCCAAGTTCAAGAAGCTGTGGAAAAGTGGAGAAATCCCCCCAGAACAACATGTT

GCTTCCAGTGAATCTCCCCCGTGCACGTCTCCACCAACTACCGCCTGGGACTTTCCACAG

ACTCAAAGAATGAACAATGTCAGCTCTAGTTTACCTCAGAGCAGCAGCCCTCCAAACACG

ACTGCGCCTTCGTCGTTTTTGGCAAACTACTCCTGGTACTCAACTACGAACTCTGCCACG

CATCTGCAGCCTCCTCTGGTTCAGCATCACCACAACTCCGCCATA

>dlx2a_Neofur

CAGATTACCTCAAGCAATTACCACAGCTTGCACAAATCGCAGGAGTCCCCGACTCTGCCG

GTTTCCACGGCGACGGACAGCAGCTATTACAACGGCCAGCAGCCTGCGCACTGCGCCGGG

TCACCGTTTGGACAACTGGGCACTTACCAGTACCACAGCAGCGCCACGAGTTCTGTGCCA

TATAACGCAAAGTCATACGACCTCGGTTTCAACTCATCGTATGGTACATACGGTTCTTAT

GGCTCCAACTCATCGCCAACTCCCGCAGACACAGAGAAAGATGAGAGCGAGCCAGAAATC

CGGATGGTTAATGGAAAACCAAAGAAGGTCAGGAAACCTCGAACCATTTACTCCAGCTTC

CAACTGGCTGCACTTCAACGGAGGTTTCAAAAGACTCAGTATTTGGCTCTACCAGAACGG

GCCGAGCTGGCAGCGTCGCTGGGCCTTACGCAAACACAGGTTAAAATCTGGTTCCAAAAC

CGGCGCTCCAAGTTCAAGAAGCTGTGGAAAAGTGGAGAAATCCCCCCAGAACAACATGTT

GCTTCCAGTGAATCTCCCCCGTGCACGTCTCCACCAACTACCGCCTGGGACTTTCCACAG

ACTCAAAGAATGAACAATGTCAGCTCTAGTTTACCTCAGAGCAGCAGCCCTCCAAACACG

ACTGCGCCTTCGTCGTTTTTGGCAAACTACTCCTGGTACTCAACTACGAACTCTGCCACG

CATCTGCAGCCTCCTCTGGTTCAGCATCACCACAACTCCGTCCTA

>dlx2a_Neopul

--GATTACCTCAAGCAATTACCACAGCTTGCACAAATCGCAGGAGTCCCCGACTCTGCCG

GTTTCCACGGCGACGGACAGCAGCTATTACAACGGCCAGCAGCCTGCGCACTGCGCCGGG

TCACCGTTTGGACAACTGGGCACTTACCAGTACCACAGCAGCGCCACGAGTTCTGTGCCA

TATAACGCAAAGTCATACGACCTCGGTTTCAACTCATCGTATGGTACATACGGTTCTTAT

GGCTCCAACTCATCGCCAACTCCCGCAG--------------------------------

------------------------------------------------------------

------------------------------------------------------------

---------------------------------ACACAGGTTAAAATCTGGTTCCAAAAC

CGCCGCTCCAAGTTCAAGAAGCTGTGGAAAAGTGGAGAAATCCCCCCAGAACAACATGTT

GCTTCCAGTGAATCTCCCCCGTGCACGTCTCCACCAACTACCGCCTGGGACTTTCCACAG

ACTCAAAGAATGAACAATGTCAGCTCTAGTTTACCTCAGAGCAGCAGCCCTCCAAACACG

ACTGCGCCTTCGTCGTTTTTGGCAAACTACTCCTGGTACTCAACTACGAACTCTGCCACG

CATCTGCAGCCTCCTCTGGTTCAGCATCACCACAACTCCGTCATA

>dlx2a_Plesta

---------------------------------------------TCCCCGACTCTGCCG

GTTTCCACGGCGACGGACAGCAGCTATTACAACGGCCAGCAGCCTGGGCACTGCGCCGGG

TCACCGTTTGGACAACTGGGCACTTACCAGTACCACAGCAGCGCCACGAGTTCTGTGCCA

TATAACGCGAAGTCATACGACCTCGGTTTCAACTCATCGTATGGTACATACGGTTCTTAT

GGCTCCAACTCATCGCCAACTCCCGCAGACACAGAGAAAGATGAGAGCGAGCCAGAAATC

CGGATGGTTAATGGAAAACCAAAGAAGGTCAGGAAACCTCGAACCATTTACTCCAGCTTC

CAACTGGCTGCACTTCAACGGAGGTTTCAAAAGACTCAGTATTTGGCTCTACCAGAACGG

GCCGAGCTGGCAGCGTCGCTGGGCCTTACGCAAACACAGGTTAAAATCTGGTTCCAAAAC

CGTCGCTCCAAGTTCAAGAAGCTGTGGAAAAGTGGAGAAATCCCCCCAGAACAACATGTT

GCTTCCAGTGAATCTCCCCCGTGCACGTCTCCACCAACTACCGCCTGGGACTTTCCACAG

ACTCAAAGAATGAACAATGTCAGCTCTAGTTTACCTCAGAGCAGCAGCCCTCCAAACACG

ACTGCGCCTTCGTCGTTTTTGGCAAACTACTCCTGGTACTCAACTACGAACTCTGCCACG

CATCTGCAGCCTCCTCTGGTTCAGCATCACCACAACTCCGCCATA

>dlx2a_Oretan

---------------------------------------------TCCCCGACTCTGCCG

GTTTCCACGGCGACGGACAGCAGCTATTACAACGGCCAGCAGCCTGGGCACTGCGCCGGG

TCACCGTTTGGACAGCTGGGCACTTACCAGTACCACAGCAGCGCCACGAGTTCTGTGCCA

TATAACGCAAAGTCATACGACCTCGGTTTCAACTCATCGTATGGTACATACGGTTCTTAT

GGCTCTAACTCATCGCCAACTCCCGCAGACACAGAGAAAGATGAGAGCGAGCCAGAAATC

CGGATGGTTAATGGAAAACCAAAGAAGGTTAGGAAACCTCGAACCATTTACTCCAGCTTC

CAACTGGCTGCACTTCAACGGAGGTTTCAAAAGACTCAGTATTTGGCTCTACCAGAACGG

GCCGAGCTGGCAGCGTCGCTGGGCCTTACGCAAACACAGGTTAAAATCTGGTTCCAAAAC

CGCCGCTCCAAGTTCAAGAAGCTGTGGAAAAGTGGAGAAATCCCCCCAGAACAACATGTT

GCTTCCAGTGAATCTCCCCCGTGCACGTCTCCACCAACTACCGCCTGGGATTTTCCACAG

ACTCAAAGAATGAACAATGTCAGCTCTAGTTTACCTCAGAGCAGCAGCCCTCCAAACACG

ACTGCGCCTTCGTCGTTTTTGGCTAACTACTCCTGGTACTCAACTACGAACTCTGCCACG

CATCTGCAGCCTCCTCTGGTTCAGCATCACCACAACTCCGCCATA

>dlx2a_Gnaper

------------------------------------------------------------

------------------------------------------------------------

------------------------------------------------------------

------------------------------------------------------------

------------------------------------------------------------

------------GGAAAACCAAAGAAGGTCAGGAAACCTCGAACCATTTACTCCAGCTTC

CAACTGGCTGCACTTCAACGGCGGTTTCAAAAGACTCAGTATTTGGCTCTACCAGAACGG

GCCGAGCTGGCAGCGTCGCTGGGCCTTACGCAAACACAAGTTAAAATCTGGTTCCAAAAC

CGCCGCTCCAAGTTCAAGAAGATGTGGAAAAGTGGAGAAATCCCCCCAGAACAACATGTT

GCTTCCAGTGAATCTCCCCCGTGCACGTCTCCACCAACTACCGCCTGGGACTTTCCACAG

ACTCAAAGAATGAACAATGTCAGCTCTAGTTTACCTCAGAGCAGCAGCCCTCCAAACACG

ACTGCGCCTTCGTCGTTTTTGGCAAACTACTCCTGGTACTCAACTACGAACTCTGCCACG

CATCTGCAGCCTCCTCTGGTTCAGCATCACCACAACTCCGCCATA

Dlx3a input file:

>dlx3a_A.burtoni

TGTTCCATCTCGGCTTCCAAGGATTCCCCGACCATGCCGGAGTCCTCCTCCACAGATATG

GGCTTCTATAGCGGCCAGAGCGCGCTCCACGGCTCGCAGGATTTCTACCCGGCACAGCAG

CCGTACTCTGCTCAGCACATGAACCCGTACGCATACCACCACTACAGCCTCAACGGAATG

GGTCCCGGCGGTGCCTACCCCGTTGGGAAGGCAGAGTACCCTTACCCCCATGCAGCATAC

AGGGAGCACGGAGCATTCAACAGAGAGTTTAAGAAGCTCTACAAGAACGGAGAGTTTCCG

CTCGGTGATATTCCTCTTGAACACAGTCCAGACGCCAGCGACTCCATGGCCTGCAACTCT

CCTCCATCCCCAGCTGTGTGGGAAAACAACAACAGTAGCAATGGTAACAATAACAGCAGC

AACCACAGCAGCGGGAGCGGCAACAACCCAAACGTCAGCAACGGTAGCGTCAAAAATAGT

GCAATGCTGGATCCTACCAGTAGGGGGCAGGTTCCCTATCAGCCTCCGGTGGATTCTCCA

CCTGCCTATATGGGGGAGTACACACACCAGAACTGGTACCAACAGCAGGGTGCACACTTA

GGTCTCTCACAGTCAGGCCAAGTGCACCACGCACCACCAACTGCTCCGTCGGCCACACAG

AGTATG

>dlx3a_A.fasciatus

TGTTCCATCTCGGCTTCCAAGGATTCCCCGACCATGCCGGAGTCCTCCTCCACAGATATG

GGCTTCTATAGCGGCCAGAGCGCGCTCCACGGCTCGCAGGATTTCTACTCGGCACAGCAG

CCGTACTCCGCTCAGCACATGAACCCGTACGCATACCACCACTACAGCCTGAACGGAATG

GGTCCCGGCGGTGCCTACCCCGTTGGGAAGGCAGAGTACCCTTACCCCCATGCAGCATAC

AGGGAGCACGGAGCATTCAACAGAGAGTTTAAGAAGCTCTACAAGAACGGAGAGTTTCCG

CTCGGTGATATTCCTCTTGAACACAGTCCAGACGCCAGCGACTCCATGGCCTGCAACTCT

CCTCCATCCCCAGCTGTGTGGGAAAACAACAACAGTAGCAATGGTAACAATAACAGCAGC

AACCACAGCAGCGGGAGCGGCAACAACCCAAACGTCAGCAACGGTAGCGTCAAAAATAGT

GCAATGCTGGATCCTACCAGTAGGGGGCAGGTTCCCTATCAGCCTCCGGTGGATTCTTCA

CCTGCCTATATGGGGGAATACACACACCAGAACTGGTACCAACAGCAGGGTGCACACTTA

GGTCTCTCACAGTCAGGCCAAGTGCACCACGCACCACCAACTGCTCCGTCGGCCACACAG

AGTATG

>dlx3a_B.graueri

TGTTCCATCTCGGCTTCCAAGGATTCCCCGACCATGCCGGAGTCCTCCTCCACAGATATG

GGCTTCTATAGCGGCCAGAGCGCGCTCCACAGCTCGCAGGATTTCTACCCGGCACAGCAG

CCGTACTCCGCTCARCACATGAACCCGTACGCATACCACCACTACAGCCTGAACGGAATG

GGTCCCGGCGGTGCCTACCCCGTTGGGAAGGCAGAGTATCCTTACCCCCATGCAGCATAC

AGGGAGCACGGAGCATTCAACAGAGAGTTTAAGAAGCTCTACAAGAACGGAGAGTTTCCG

CTCGGTGATATTCCTCTTGAACACAGTCCAGACGCCAGCGACTCCATGGCCTGCAACTCT

CCTCCATCCCCAGCTGTGTGGGAAAACAACAACAGTAGCAATGGTAACAATAACAGCAGC

AACCACAGCAGCGGGAGCGGCAACAACCCAAACGTCAGCAACGGTAGTGTCAAAAATAGT

GCAATGCTGGATCCTACCAGTAGGGGGCAGGTTCCCTATCAGCCTCCGGTGGATTCTTCA

CCTGCCTATATGGGGGAGTACACACACCAGAACTGGTACCAACAGCAGGGTGCACACTTA

GGTCTCTCACAGTCAGGCCAAGTGCACCACGCACCACCAACTGCTCCGTCGGCCACACAG

AGTATG

>dlx3a_B.microlepis

TGTTCCATCTCGGCTTCCAAGGATTCCCCGACCATGCCGGAGTCCTCCTCCACAGATATG

GGCTTCTATAGCGGCCAGAGCGCGCTCCACAGCTCGCAGGATTTCTACCCGGCACAGCAG

CCGTACTCCGCTCAGCACATGAACCCGTACGCATACCACCACTACAGCCTGAACGGAATG

GGTCCCGGCGGTGCCTACCCCGTTGGGAAGGCAGAGTACCCTTACCCCCATGCAGCATAC

AGGGAGCACGGAGCATTCAACAGAGAGTTTAAGAAGCTCTACAAGAACGGAGAGTTTCCG

CTCGGTGATATTCCTCTTGAACACAGTCCAGACGCCAGCGACTCCATGGCCTGCAACTCT

CCTCCATCCCCAGCTGTGTGGGAAAACAACAACAGTAGCAATGGTAACAATAACAGCAGC

AACCACAGCAGCGGGAGCGGCAACAACCCAAACGTCAGCAACGGTAGCGTCAAAAATAGT

GCAATGCTGGATCCTACCAGTAGGGGGCAGGTTCCCTATCAGCCTCCGGTGGATTCTTCA

CCTGTCTATATGGGGGAGTACACACACCAGAACTGGTACCAACAGCAGGGTGCACACTTA

GGTCTCTCACAGTCAGGCCAAGTGCACCACGCACCACCAACTGCTCCGTTGGCCACACAG

AGTATG

>dlx3a_C.frontosa

TGTTCCATCTCGGCTTCCAAGGATTCCCCGACCATGCCGGAGTCCTCCTCCACAGATATG

GGCTTCTATAGCGGCCAGAGCGCGCTCCACGGCTCGCAGGATTTCTACTCGGCACAGCAG

CCGTACTCCGCTCAGCACATGAACCCGTACGCATACCACCACTACAGCCTGAACGGAATG

GGTCCCGGCGGTGCCTACCCCGTTGGGAAGGCAGAGTACCCTTACCCCCATGCAGCATAC

AGGGAGCACGGAGCATTCAACAGAGAGTTTAAGAAGCTCTACAAGAACGGAGAGTTTCCG

CTCGGTGATATTCCTCTTGAACACAGTCCAGACGCCAGCGACTCCATGGCCTGCAACTCT

CCTCCATCCCCAGCTGTGTGGGAAAACAACAACAGTAGCAATGGTAACAATAACAGCAGC

AACCACAGCAGCGGGAGCGGCAACAACCCAAACGTCAGCAACGGTAGCGTCAAAAATAGT

GCAATGCTGGATCCTACCAGTAGGGGGCAGGTTCCCTATCAGCCTCCGGTGGATTCTTCA

CCTGCCTATATGGGGGAGTACACACACCAGAACTGGTACCAACAGCAGGGTGCACACTTA

GGTCTCTCACAGTCAGGCCAAGTGCACCACGCACCACCAACTGCTCCGTCGGCCACACAG

AGTATG

>dlx3a_C.furcifer

TGTTCCATCTCGGCTTCCAAGGATTCCCCGACCATGCCGGAGTCCTCCTCCACAGATATG

GGCTTCTATAGCGGCCAGAGCGCGCTCCACGGCTCGCAGGATTTCTACCCGGCACAGCAG

ACGTACTCCGCTCAGCACATGAACCCGTACGCATACCACCACTACAGCCTGAACGGAATG

GGTCCCGGCGGTGCCTACCCCGTTGGGAAGGCAGAGTACCCTTACCCCCATGCAGCATAC

AGGGAGCACGGAGCATTCAACAGAGAGTTTAAGAAGCTCTACAAGAACGGAGAGTTTCCG

CTCGGTGATATTCCTCTTGAACACAGTCCAGACGCCAGCGACTCCATGGCCTGCAACTCT

CCTCCATCCCCAGCTGTGTGGGAAAACAACAACAGCAGCAATGCTAACAATAACAGCAGC

AACCACAGCAGCGGGAGCGGCAACAACCCAAACGTCAGCAACGGTAGCGTCAAAAATAGT

GCAATGCTGGATCCTACCAGTAGGGGGCAGGTTCCCTATCAGCCTCCGGTGGATTCTTCA

CCTGCCTATATGGGGGAGTACACACACCAGAACTGGTACCAACAGCAGGGTGCACACTTA

GGTCTCTCACAGTCAGGCCAAGTGCACCACGCACCACCAACTGCTCCGTCGGCCACACAG

AGTATG

>dlx3a_C.horei

TGTTCCATCTCGGCTTCCAAGGATTCCCCGACCATGCCGGAGTCCTCCTCCACAGATATG

GGCTTCTATAGCGGCCAGAGCGCGCTCCACGGCTCGCAGGATTTCTACCCGGCACAGCAG

CCGTACTCCGCTCAGCACATGAACCCGTACGCATACCACCACTACAGCCTGAACGGAATG

GGTCCCGGCGGTGCCTACCCCGTTGGGAAGGCAGAGTACTCTTACCCCCATGCAGCATAC

AGGGAGCACGGAGCATTCAACAGAGAGTTTAAGAAGCTCTACAAGAACGGAGAGTTTCCG

CTCGGTGATATTCCTCTTGAACACAGTCCAGACGCCAGCGACTCCATGGCCTGCAACTCT

CCTCCATCGCCAGCTGTGTGGGAAAACAACAACAGTAGCAATGGTAACAATAACAGCAGC

AACCACAGCAGCGGGAGCGGCAACAACCCAAACGTCAGCAACGGTAGCGTCAAAAATAGT

GCAATGCTGGATCCTACCAGTAGGGGGCAGGTTCCCTATCAGCCTCCGGTGGATTCTTCA

CCTGCCTATATGGGGGAGTACACACACCAGAACTGGTACCAACAGCAGGGTGCACACTTA

GGTCTCTCACAGTCAGGCCAAGTGCACCACGCACCACCAACTGCTCCGTCGGCCACACAG

AGTATG

>dlx3a_C.leptosoma

TGTTCCATCTCGGCTTCCAAGGATTCCCCGACCATGCCGGAGTCCTCCTCCACAGATATG

GGCTTCTATAGCGGCCAGAGCGCGCTCCACGGCTCGCAGGATTTCTACCCGGCACAGCAG

CCGTACTCCGCTCAGCACATGAACCCGTACGCATACCACCACTACAGCCTGAACGGAATG

GGTCCCGGCGGTGCCTACCCCGTTGGGAAGGCAGAATACCCTTACCCCCATGCAGCATAC

AGGGAGCACGGAGCATTCAACAGAGAGTTTAAGAAGCTCTACAAGAACGGAGAGTTTCCG

CTCGGTGATATTCCTCTTGAACACAGTCCAGACGCCAGCGACTCCATGGCCTGCAACTCT

CCTCCATCCCCAGCTGTGTGGGAAAACAACAACAGTAGCAATGGTAACAATAACAGCAGC

AACCACAGCAGCGGGAGCGGCAACAACCCAAACGTCAGCAACGGTAGCGTCAAAAATAGT

GCAATGCTGGATCCTACCAGTAGGGGGCAGGTTCCCTATCAGCCTCCGGTGGATTCTTCA

CCTGCCTATATGGGGGAGTACACACACCAGAACTGGTACCAACAGCAGGGTACACACTTA

GGTCTCTCACAGTCAGGCCAAGTGCACCACGCACCACCAACTGCTCCATCGGCCACACAG

AGTATG

>dlx3a_C.macrops

TGTTCCATCTCGGCTTCCAAGGATTCCCCGACCATGCCGGAGTCCTCCTCCACAGATATG

GGCTTCTATAGCGGCCAGAGCGCGCTCCACGGCTCGCAGGATTTCTACCCTGCACAGCAG

CCGTACTCCGCTCAGCACATGAACCCGTACGCATACCACCACTACAGCCTGAACGGAATG

GGTCCCGGCGGTGCCTACCCCGTTGGGAAGGCAGAGTACCCTTACCCCCATGCAGCATAC

AGGGAGCACGGAGCATTCAACAGAGAGTTTAAGAAGCTCTACAAGAACGGAGAGTTTCCG

CTCGGTGATATTCCTCTTGAACACAGTCCAGACGCCAGCGACTCCATGGCCTGCAACTCT

CCTCCATCCCCAGCTGTGTGGGAAAACAACAACAGTAGCAATGGTAACAATAACAGCAGC

AACCACAGCAGCGGGAGCGGCAACAACCCAAACGTCAGCAACGGTAGCGTCAAAAGTAGT

GCAATGCTGGATCCTACCAGTAGGGGGCAGGTTCCCTATCAGCCTCCGGTGGATTCTTCA

CCTGCCTATATGGGGGAGTACACACACCAGAACTGGTACCAACAGCAGGGTGCACACTTA

GGTCTCTCACAGTCAGGCCAAGTGCACCACGCACCACCAACTGCTCCGTCGGCCACACAG

AGTATG

>dlx3a_G.permaxillaris

TGTTCCATCTCGGCTTCCAAGGATTCCCCGACCATGCCGGAGTCCTCCTCCACAGATATG

GGCTTCTATAGCGGCCAGAGCGCGCTCCACGGCTCGCAGGATTTCTACTCGGCACAGCAG

CCGTACTCCGCTCAGCACATGAACCCGTACGCATACCACCACTACAGCCTGAACGGAATG

GGTCCCGGCGGTGCCTACCCCGTTGGGAAGGCAGAGTACCCTTACCCCCATGCAGCATAC

AGGGAGCACGGAGCATTCAACAGAGAGTTTAAGAAGCTCTACAAGAACGGAGAGTTTCCG

CTCGGTGATATTCCTCTTGAACACAGTCCAGACGCCAGCGACTCCATGGCCTGCAACTCT

CCTCCATCCCCAGCTGTGTGGGAAAACAACAACAGTAGCAATGGTAACAATAACAGCAGC

AACCACAGCAGCGGGAGCGGCAACAACCCAAACGTCAGCAACGGTAGCGTCAAAAATAGT

GCAATGCTGGATCCTACCAGTAGGGGGCAGGTTCCCTATCAGCCTCCGGTGGATTCTTCA

CCTGCCTGTATGGGGGAGTACACACACCAGAACTGGTACCAACAGCAGGGTGCACACTTA

GGTCTCTCACAGTCAGGCCAAGTGCACCACGCACCACCAACTGCTCCGTCGGCCACACAG

AGTATG

>dlx3a_L.dardenii

--------------------------------------------------CACAGATATG

GGCTTCTATAGCGGCCAGAGCGCGCTCCACGGCTCGCAGGATTTCTACCCGGCACAGCAG

CCGTACTCCGCTCAGCACATGAACCCGTACGCATACCACCACTACAGCCTGAACGGAATG

GGTCCCGGCGGTGCCTACCCCGTTGGGAAGGCAGAGTACTCTTACCCCCATGCAGCATAC

AGGGAGCACGGAGCATTCAACAGAGAGTTTAAGAAGCTCTACAAGAACGGAGAGTTTCCG

CTCGGTGATATTCCTCTTGAACACAGTCCAGACGCCAGCGACTCCATGGCCTGCAACTCT

CCTCCATCCCCAGCTGTGTGGGAAAACAACAACAGTAGCAATGGTAACAATAACAGCAGC

AACCACAGCAGCGGGAGCGGCAACAACCCAAACGTCAGCAACGGTAGCGTCAAAAATAGT

GCAATGCTGGATCCTACCAGTAGGGGGCAGGTTCCCTATCAGCCTCCGGTGGATTCTTCA

CCTGCCTATATGGGGGAGTACACACACCAGAACTGGTACCAACAGCAGGGTGCACACTTA

GGTCTCTCACAGTCAGGCCAAGTGCACCACGCACCACCAACTGCTCCGTCGGCCACACAG

AGTATG

>dlx3a_L.elongatus

TGTTCCATCTCGGCTTCCAAGGATTCCCCGACCATGCCGGAGTCCTCCTCCACAGATATG

GGCTTCTATAGCGGCCAGAGCGCGCTCCACGGCTCGCAGGATTTCTACTCTGCACAGCAG

CCGTACTCCGCTCAGCACATGAACCCGTACGCATACCACCACTACAGCCTGAACGGAATG

GGTCCCGGCGGTGCCTACCCCGTTGGGAAGGCAGAGTACCCTTACCCCCATGCAGCATAC

AGGGAGCACGGAGCATTCAACAGAGAGTTTAAGAAGCTCTACAAGAACGGAGAGTTTCCG

CTCGGTGATATTCCTCTTGAACACAGTCCAGACGCCAGCGACTCCATGGCCTGCAACTCT

CCTCCATCCCCAGCTGTGTGGGAAAACAACAACAGTAGCAATGGTAACAATAACAGCAGC

AACCACAGCAGCGGGAGCGGCAACAACCCAAACGTCAGCAATGGTAGCGTCAAAAATAGT

GCAATGCTGGATCCTACCAGTAGGGGGCAGGTTCCCTATCAGCCTCCGGTGGATTCTTCA

CCTGCCTATATGGGGGAATACACACACCAGAACTGGTACCAACAGCAGGGTGCACACTTA

GGTCTCTCACAGTCAGGCCAAGTGCACCACGCACCACCAACTGCTCCGTCGGCCACACAG

AGTATG

>dlx3a_L.labiatus

TGTTCCATCTCGGCTTCCAAGGATTCCCCGACCATGCCGGAGTCCTCCTCCACAGATATG

GGCTTCTATAGCGGCCAGAGCGCGCTCCACGGCTCGCAGGATTTCTACCCGGCACAGCAG

CCGTACTCCGCTCAGCACATGAACCCGTACGCATACCACCACTACAGCCTGAACGGAATG

GGTCCCGGCGGTGCCTACCCCGTTGGGAAGGCAGAGTACTCTTACCCCCATGCAGCATAC

AGGGAGCACGGAGCATTCAACAGAGAGTTTAAGAAGCTCTACAAGAACGGAGAGTTTCCG

CTCGGTGATATTCCTCTTGAACACAGTCCAGACGCCAGCGACTCCATGGCCTGCAACTCT

CCTCCATCCCCAGCTGTGTGGGAAAACAACAACAGTAGCAATGGTAACAATAACAGCAGC

AACCACAGCAGCGGGAGCGGCAACAACCCAAACGTCAGCAACGGTAGCGTCAAAAATAGT

GCAATGCTGGATCCTACCAGTAGGGGGCAGGTTCCCTATCAGCCTCCGGTGGATTCTTCA

CCTGCCTATATGGGGGAGTACACACACCAGAACTGGTACCAACAGCAGGGTGCACACTTA

GGTCTCTCACAGTCAGGCCAAGTGCACCACGCACCACCAACTGCTCCGTCGGCCGCACAG

AGTATG

>dlx3a_L.staneri

TGTTCCATCTCGGCTTCCAAGGATTCCCCGACCATGCCGGAGTCCTCC---ACAGATATG

GGCTTCTATAGCGGCCAGAGCGCGCTCCACGGCTCGCAGGATTTCTACCCGGCACAGCAG

CCGTACTCCGCTCAGCACATGAACCCGTACGCATACCACCACTACAGCCTGAACGGAATG

GGTCCCGGCGGTGCCTACCCCGTTGGGAAGGCAGAGTACCCTTACCCCCATGCAGCATAC

AGGGAGCACGGAGCATTCAACAGAGAGTTTAAGAAGCTCTACAAGAACGGAGAGTTTCCG

CTCGGTGATATTCCTCTTGAACACAGTCCAGACGCCAGCGACTCCATGGCCTGCAACTCT

CCTCCATCCCCAGCTGTGTGGGAAAACAACAACAGTAGCAATGGTAACAATAACAGCAGC

AACCACAGCAGCGGGAGCGGCAACAACCCAAACGTCAGCAACGGTAGCGTCAAAAATAGT

GCAATGCTGGATCCTACCAGTAGGGGGCAGGTTCCCTATCAGCCTCCGGTGGATTCTTCA

CCTGCCTATATGGGGGAGTACACACACCAGAACTGGTACCAACAGCAGGGTGCACACTTA

GGTCTCTCACAGTCAGGCCAAGTGCACCACGCACCACCAACTGCTCCGTCGGCCACACAG

AGTATG

>dlx3a_N.furcifer

TGTTCCATCTCGGCTTCCAAGGATTCCCCGACAATGCCGGAGTCCTCCTCCACAGATATG

GGCTTCTATAGCGGCCAGAGCGCGCTCCACGGCTCGCAGGATTTCTACTCGGCACAGCAG

CCGTACTCCGCTCAGCACATGAACCCGTACGCATACCACCACTACAGCCTGAACGGAATG

GGTCCCGGCGGTGCCTACCCCGTTGGGAAGGCAGAGTACCCTTACCCCCATGCAGCATAC

AGGGAGCACGGAGCATTCAACAGAGAGTTTAAGAAGCTCTACAAGAACGGAGAGTTTCCG

CTCGGTGATATTCCTCTTGAACACAGTCCAGACGCCAGCGACTCCATGGCCTGCAACTCT

CCTCCATCCCCAGCTGTGTGGGAAAACAACAACAGTAGCAATGGTAACAATAACAGCAGC

AACCACAGCAGCGGGAGCGGCAACAACCCAAACGTCAGCAACAGTAGCATCAAAAATAGT

GCAATGCTGAATCCTACCAGTAGGGGGCAGGTTCCCTATCAGCCTCCGGTGGATTCTTCA

CCTGCCTATATGGGGGAATACACACACCAGAACTGGTACCAACAGCAGGGTGCACACTTA

GGTCTCTCACAGTCAGGCCAAGTGCACCACGCACCACCAACTGCTCCGTCGGCCACACAG

AGTATG

>dlx3a_N.pulcher

TGTTCCATCTCGGCTTCCAAGGATTCCCCGACCATGCCGGAGTCCTCCTCCACAGATATG

GGCTTCTATAGCGGCCAGAGCGCGCTCCACGGCTCGCAGGATTTCTACTCGGCACAGCAG

CCGTACTCCGCTCAGCACATGAACCCGTACGCATACCACCACTACAGCCTGAACGGAATG

GGTCCCGGCGGTGCCTACCCCGTTGGGAAGGCAGAGTACCCTTACCCCCATGCAGCATAC

AGGGAGCACGGAGCATTCAACAGAGAGTTTAAGAAGCTCTACAAGAACGGAGAGTTTCCG

CTCGGTGATATTCCTCTTGAACACAGTCCAGACGCCAGCGACTCCATGGCCTGCAACTCT

CCTCCATCCCCAGCTGTGTGGGAAAACAACAACAGTAGCAATGGTAACAATAACAGCAGC

AACCACAGCAGCGGGAGCGGCAACAACCCAAACGTCAGCAACGGTAGCGTCAAAAATAGT

GCAATGCTGGATCCTACCAGTAGGGGGCAGGTTCCCTATCAGCCTCCGGTGGATTCTTCA

CCTGCCTATATGGGGGAATACACACACCAGAACTGGTACCAACAGCAGGGTGCACACTTA

GGTCTCTCACAGTCAGGCCAAGTGCACCACGCACCACCAACTGCTCCGTCGGCCACACAG

AGTATG

>dlx3a_O.tanganicae

TGTTCCATCTCGGCTTCCAAGGATTCCCCGACCATGCCGGAGTCCTCCTCCACAGATATG

GGCTTCTATAGCGGCCAGAGCGCGCTCCACGGCTCGCAGGATTTCTACCCGGCACAGCAG

CCGTACTCCGCTCAGCACATGAACCCGTACGCATACCACCACTACAGCCTGAACGGGATG

GGTCCCGGCGGTGCCTACCCCGTTGGGAAGGCAGAGTACCCTTACCCCCATGCAGCGTAC

AGGGAGCACGGAGCATTCAACAGAGAGTTTAAGAAGCTCTACAAGAACGGGGAGTTTCCG

CTCGGTGATATTCCTCTTGAACACAGTCCGGACGCCAGCGATTCCATGGCTTGCAACTCT

CCTCCATCCCCAGCTGTGTGGGAAAACAACAACAGCAGCAACGGTAACAATAACAGCAGC

AACCACAGCAGCGGGAGCGGCAACAACCCAAACGTCAGCAACGGTAGCATCAAAAATAGT

GCAATGCTGGATCCTACCAGTAGGGGGCAGGTTCCCTATCAGCCTCAGGTGGATTCTTCA

CCCGCCTATATGGGGGAGTACACACACCAGAACTGGTACCAACAGCAGGGTGCACACTTA

GGTCTCTCACAGTCAGGCCAAGTGCACCACGCGCCACCAACTGCTCCGTCGGCCACACAG

AGTATG

>dlx3a_P.curvifrons

TGTTCCATCTCGGCTTCCAAGGATTCCCCGACCATGCCGGAGTCCTCCTCCACAGATATG

GGCTTCTATAGCGGCCAGAGCGCGCTCCACGGCTCGCAGGATTTCTACCCGGCACAGCAG

CCGTACTCCGCTCAGCACATGAACCCGTACGCATACCACCACTACAGCCTGAACGGAATG

GGTCCCGGCGGTGCCTACCCCGTTGGGAAGGCAGAGTACTCTTACCCCCATGCAGCATAC

AGGGAGCACGGAGCATTCAACAGAGAGTTTAAGAAGCTCTACAAGAACGGAGAGTTTCCG

CTCGGTGATATTCCTCTTGAACACAGTCCAGACGCCAGCGACTCCATGGCCTGCAACTCT

CCTCCATCCCCAGCTGTGTGGGAAAACAACAACAGTAGCAATGGTAACAATAACAGCAGC

AACCACAGCAGCGGGAGCGGCAACAACCCAAACGTCAGCAACGGTAGCGTCAAAAATAGT

GCAATGCTGGATCCTACCAGTAGGGGGCAGGTTCCCTATCAGCCTCCGGTGGATTCTTCA

CCTGCCTATATGGGGGAGTACACACACCAGAACTGGTACCAACAGCAGGGTGCACACTTA

GGTCTCTCACAGTCAGGCCAAGTGCACCACGCACCACCAACTGCTCCGTCGGCCACACAG

AGTATG

>dlx3a_P.famula

TGTTCCATCTCGGCTTCCAAGGATTCCCCGACCATGCCGGAGTCCTCCTCCACAGATATG

GGCTTCTATAGCGGCCAGAGCGCGCTCCACGGCTCGCAGGATTTCTACCCGGCACAGCAG

CCGTACTCCGCTCAGCACATGAACCCGTACGCATACCACCACTACAGCCTGAACGGAATG

GGTCCCGGCGGTGCCTACCCCGTTGGGAAGGCAGAGTACTCTTACCCCCATGCAGCATAC

AGGGAGCACGGAGCATTCAACAGAGAGTTTAAGAAGCTCTACAAGAACGGAGAGTTTCCG

CTCGGTGATATTCCTCTTGAACACAGTCCAGACGCCAGCGACTCCATGGCCTGCAACTCT

CCTCCATCCCCAGCTGTGTGGGAAAACAACAACAGTAGCAATGGTAACAATAACAGCAGC

AACCACAGCAGCGGGAGCGGCAACAACCCAAACGTCAGCAACGGTAGCGTCAAAAATAGT

GCAATGCTGGATCCTACCAGTAGGGGGCAGGTTCCCTATCAGCCTCCGGTGGATTCTTCA

CCTGCCTATATGGGGGAGTACACACACCAGAACTGGTACCAACAGCAGGGTGCACACTTA

GGTCTCTCACAGTCAGGCCAAGTGCACCACGCACCACCAACTGCTCCGTCGGCCACACAG

AGTATG

>dlx3a_P.microlepis

TGTTCCATCTCGGCTTCCAAGGATTCCCCGACCATGCCGGAGTCCTCCTCCACAGATATG

GGCTTCTATAGCGGCCAGAGCGCGCTCCACGGCTCGCAGGATTTCTACCCGGCACAGCAG

CCGTACTCCGCTCAGCACATGAACCCGTACGCATACCACCACTACAGCCTGAACGGAATG

GGTCCCGGCGGTGCCTACCCCGTTGGGAAGGCAGAGTACCCTTACCCCCATGCAGCATAC

AGGGAGCACGGAGCATTCAACAGAGAGTTTAAGAAGCTCTACAAGAACGGAGAGTTTCCG

CTCGGTGATATTCCTCTTGAACACAGTCCAGACGCCAGCGACTCCATGGCCTGCAACTCT

CCTCCATCCCCAGCTGTGTGGGAAAACAACAACAGTAGCAATGGTAACAATAACAGCAGC

AACCACAGCAGCGGGAGCGGCAACAACCCAAACGTCAGCAACGGTAGCGTCAAAAATAGT

GCAATGCTGGATCCTACCAGTAGGGGGCAGGTTCCCTATCAGCCTCCGGTGGATTCTTCA

CCTGCCTATATGGGGGAGTACACACACCAGAACTGGTACCAACAGCAGGGTGCACACTTA

GGTCTCTCACAGTCAGGCCAAGTGCACCACGCACCACCAACTGCTCCGTCGGCCACACAG

AGTATG

>dlx3a_P.straeleni

TGTTCCATCTCGGCTTCCAAGGATTCCCCGACCATGCCGGAGTCCTCCTCCACAGATATG

GGCTTCTATAGCGGCCAGAGCGCGCTCCACGGCTCGCAGGATTTCTACCCGGCACAGCAG

CCGTACTCCGCTCAGCACATGAACCCGTACGCATACCACCACTACAGCCTGAACGGAATG

GGTCCCGGCGGTGCCTACCCCGTTGGGAAGGCAGAGTACCCTTACCCCCATGCAGCATAC

AGGGAGCACGGAGCATTCAACAGAGAGTTTAAGAAGCTCTACAAGAACGGAGAGTTTCCG

CTCGGTGATATTCCTCTTGAACACAGTCCAGACGCCAGCGACTCCATGGCCTGCAACTCT

CCTCCATCCCCAGCTGTGTGGGAAAACAACAACAGTAGCAATGGTAACAATAACAGCAGC

AACCACAGCAGCGGGAGCGGCAACAACCCAAACGTCAGCAACGGTAGCGTCAAAAATAGT

GCAATGCTGGATCCTACCAGTAGGGGGCAGGTTCCCTATCAGCCTCCGGTGGATTCTTCA

CCTGCCTATATGGGGGAGTACACACACCAGAACTGGTACCAACAGCAGGGTGCACACTTA

GGTCTCTCACAGTCAGGCCAAGTGCACCACGCACCACCAACTGCTCCGTCGGCCACACAG

AGTATG

>dlx3a_T.moorii

TGTTCCATCTCGGCTTCCAAGGATTCCCCGACCATGCCGGAGTCCTCCTCCACAGATATG

GGCTTCTATAGCGGCCAGAGCGCGCTCCACGGCTCGCAGGATTTCTACCCGGCACAGCAG

CCGTACTCCGCTCAGCACATGAACCCGTACGCATACCACCACTACAGCCTGAACGGAATG

GGTCCCGGCGGTGCCTACCCCGTTGGGAAGGCAGAGTACCCTTACCCCCATGCAGCATAC

AGGGAGCACGGAGCATTCAACAGAGAGTTTAAGAAGCTCTACAAGAACGGAGAGTTTCCG

CTCGGTGATATTCCTCTTGAACACAGTCCAGACGCCAGCGACTCCATGGCCTGCAACTCT

CCTCCATCCCCAGCTGTGTGGGAAAACAACAACAGTAGCAATGGTAACAATAACAGCAGC

AACCACAGCAGCGGGAGCGGCAACAACCCAAACGTCAGCAACGGTAGCGTCAAAAATAGT

GCAATGCTGGATCCTACCAGTAGGGGGCAGGTTCCCTATCAGCCTCCGGTGGATTCTTCA

CCTGCCTATATGGGGGAGTACACACACCAGAACTGGTACCAACAGCAGGGTGCACACTTA

GGTCTCTCACAGTCAGGCCAAGTGCACCACGCACCACCAACTGCTCCGTCGGCCACACAG

AGTATG

>dlx3a_V.moorii

TGTTCCATCTCGGCTTCCAAGGATTCCCCGACAATGCCGGAGTCCTCCTCCACAGATATG

GGCTTCTATAGCGGCCAGAGCGCGCTCCACGGCTCGCAGGATTTCTACTCGGCACAGCAG

CCGTACTCCGCTCAGCACATGAACCCGTACGCATACCACCACTACAGCCTGAACGGAATG

GGTCCCGGCGGTGCCTACCCCGTTGGGAAGGCAGAGTACCCTTACCCTCATGCAGCATAC

AGGGAGCACGGAGCATTCAACAGAGAGTTTAAGAAGCTCTACAAGAACGGAGAGTTTCCG

CTCGGTGATATTCCTCTTGAACACAGTCCAGACGCCAGCGACTCCATGGCCTGCAACTCT

CCTCCATCCCCAGCTGTGTGGGAAAACAACAACAGTAGCAATGGTAACAATAACAGCAGC

AACCACAGCAGCGGGAGCGGCAACAACCCAAACGTCAGCAACGGTAGCGTCAAAAATAGT

GCAATGCTGGATCCTACCAGTAGGGGGCAGGTTCCCTATCAGCCTCCGGTGGATTCTTCA

CCTGCCTATATGGGGGAATACACACACCAGAACTGGTACCAACAGCAGGGTGCACACTTA

GGTCTCTCACAGTCAGGCCAAGTGCACCACGCACCACCAACTGCTCCGTCGGCCACACAG

AGTATG

Dlx3b input file:

>dlx3b_Altfas

GACTCCCCTACTCTGCCCGAGTCGTCCGTGACGGACATGGGCTACTACAGTGGACAGACG

GCCCACGGCCATCATGAATATTATCAGAGTCAGCCGTACGGGCAGCCCATGAACTCTTAC

CATCACCAGTTTAATCTGAACGGAATGGGAGCTGCTGGAGCGTACGCCACCAAATCTGAA

TACCCATACACGGTGAAAGAAGAGCCAGAGCCGGAGGTTCGTATGGTAAATGGAAAGCCG

AAAAAGATTCGCAAGCCGAGGACGATCTACTCTTCCCCGCCATCGCCCGCAGTCTGGGAC

AACAGCAGCACCCCTCAGAACACCCCGATCAGCAGACCTCAGGTGCCGCAGCCGACGCAC

AGTTCATCGCCGCCGTACCTGGAGGATTATAATAACCACTGGTACCAGCAGGGATCACAC

CTACAACACCCGGGAGCCGTGCACCACCCAGTCCCGCAGCAAAGCGTGGGAGCTGTTTAT

>dlx3b_Astbur

GACTCCCCTACTCTGCCCGAGTCGTCCGTGACGGACATGGGCTACTACAGTGGACAGACG

GCCCACGGCCATCATGAATATTATCAGAGTCAGCCGTACGGGCAGCCCATGAACTCTTAC

CATCACCAGTTTAATCTGAACGGAATGGGAGCTGCTGGAGCGTATGCCACCAAATCTGAA

TACCCATACACGGTGAAAGAGGAGCCAGAGCCGGAGGTTCGTATGGTAAATGGAAAGCCG

AAAAAGATTCGCAAGCCGAGGACGATCTACTCTTCCCCGCCATCGCCCGCTGTCTGGGAC

AACAGCAGCACCCCTCAGAACACCCCGATCAGCAGACCTCAGGTGCCGCAGCCGACGCAC

AGTTCATCGCCGCCGTACCTGGAGGATTATAATAACCACTGGTACCAGCAGGGATCACAC

CTACAACACCCGGGAGCCGTGCACCACCCAGTCCCGCAGCAAAGCGTGGGAGCTGTTTAT

>dlx3b_Boumic

GACTCCCCTACTCTGCCCGAGTCGTCCGTGACGGACATGGGCTACTACAGTGGACAGACG

GCCCACGGCCATCATGAATATTATCAGAGTCAGCCGTACGGGCAGCCCATGAACTCTTAC

CATCACCAGTTTAATCTGAACGGAATGGGAGCTGCTGGAGCGTACGCCACCAAATCTGAA

TACCCATACACGGTGAAAGAAGAGCCAGAGCCGGAGGTTCGTATGGTAAATGGAAAGCCG

AAAAAGATTCGCAAGCCGAGGACGATCTACTCTTCCCCGCCATCACCCGCAGTCTGGGAC

AACAGCAGCACCCCTCAGAACACCCCGATCAGCAGACCTCAGGTGCCGCAGCCGACGCAC

AGTTCATCGCCGCCGTACCTGGAGGATTATAATAACCACTGGTACCAGCAGGGATCACAC

CTACAACACCCGGGAGCCGTGCACCACCCAGTCCCGCAGCAAAGCGTGGGAGCTGTTTAT

>dlx3b_Calmac

GACTCCCCTACTCTGCCCGAGTCGTCCGTGACGGACATGGGCTACTACAGTGGACAGACG

GCCCACGGCCATCATGAATATTATCAGAGTCAGCCGTACGGGCAGCCCATGAACTCTTAC

CATCACCAGTTTAATCTGAACGGAATGGGAGCTGCTGGAGCGTACGCCACCAAATCTGAA

TACCCATACACGGTGAAAGAAGAGCCAGAGCCGGAGGTTCGTATGGTAAATGGAAAGCCG

AAAAAGATTCGCAAGCCGAGGACGATCTACTCTTCCCCGCCATCACCCGCAGTCTGGGAC

AACAGCAGCACCCCTCAGAACACCCCGATCAGCAGACCTCAGGTGCCGCAGCCGACGCAC

AGTTCATCGCCGCCGTACCTGGAGGATTATAATAACCACTGGTACCAGCAGGGATCACAC

CTACAACACCCGGGAGCCGTGCACCACCCAGTCCCGCAGCAAAGCGTGGGAGCTGTTTAT

>dlx3b_Ctehor

GACTCCCCTACTCTGCCCGAGTCGTCCGTGACGGACATGGGCTACTACAGTGGACAGACG

GCCCACGGCCATCATGAATATTATCAGAGTCAGCCGTACGGGCAGCCCATGAACTCTTAC

CATCACCAGTTTAATCTGAACGGAATGGGAGCTGCTGGAGCGTACGCCACCAAATCTGAA

TACCCATACACGGTGAAAGAAGAGCCAGAGCCGGAGGTTCGTATGGTAAATGGAAAGCCG

AAAAAGATTCGCAAGCCGAGGACGATCTACTCTTCCCCGCCATCGCCCGCTGTCTGGGAC

AACAGCAGCACCCCTCAGAACACCCCGATCAGCAGACCTCAGGTGCCGCAGCCGACGCAC

AGTTCATCGCCGCCGTACCTGGAGGATTATAATAACCACTGGTACCAGCAGGGATCACAC

CTACAACACCCGGGAGCCGTGCACCACCCAGTCCCGCAGCAAAGCGTGGGAGCTGTTTAT

>dlx3b_Cyafur

GACTCCCCTACTCTGCCCGAGTCGTCCGTGACGGACATGGGCTACTACAGTGGACAGACG

GCCCACGGCCATCATGAATATTATCAGAGTCAGCCGTACGGGCAGCCCATGAACTCTTAT

CATCACCAGTTTAATCTGAACGGAATGGGAGCTGCTGGAGCGTACGCCACCAAATCTGAA

TACCCATACACGGTGAAAGAAGAGCCAGAGCCGGAGGTTCGTATGGTAAATGGAAAGCCG

AAAAAGATTCGCAAGCCGAGGACGATCTACTCTTCCCCGCCATCACCCGCAGTCTGGGAC

AACAGCAGCACCCCTCAGAACACCCCGATCAGCAGACCTCAGGTGCCGCAGCCGACGCAC

AGTTCATCGCCGCCGTACCTGGAGGATTATAATAACCACTGGTACCAGCAGGGATCACAC

CTACAACACCCGGGAGCCGTGCACCACCCAGTCCCGCAGCAAAGCGTGGGAGCTGTTTAT

>dlx3b_Cypfro

GACTCCCCTACTCTGCCCGAGTCGTCCGTGACGGACATGGGCTACTACAGTGGACAGACG

GCCCACGGCCATCATGAATATTATCAGAGTCAGCCGTACGGGCAGCCCATGAACTCTTAC

CATCACCAGTTTAATCTGAACGGAATGGGAGCTGCTGGAGCGTACGCCACCAAATCTGAA

TACCCATACACGGTGAAAGAAGAGCCAGAGCCGGAGGTTCGTATGGTAAATGGAAAGCCG

AAAAAGATTCGCAAGCCGAGGACGATCTACTCTTCCCCGCCATCGCCCGCTGTCTGGGAC

AACAGCAGCACCCCTCAGAACACCCCGATCAGCAGACCTCAGGTGCCGCAGCCGACGCAC

AGTTCATCGCCGCCGTACCTGGAGGATTATAATAACCACTGGTACCAGCAGGGATCACAC

CTACAACACCCGGGAGCCGTGCACCACCCAGTCCCGCAGCAAAGCGTGGGAGCTGTTTAT

>dlx3b_Cyplep

GACTCCCCTACTCTGCCCGAGTCGTCCGTGACGGACATGGGCTACTACAGTGGACAGACG

GCCCACGGCCATCATGAATATTATCAGAGTCAGCCGTACGGGCAGCCCATGAACTCTTAC

CATCACCAGTTTAATCTGAACGGAATGGGGGCTGCTGGAGCGTACGCCACCAAATCTGAA

TACCCATACACGGTGAAAGAAGAGCCAGAGCCGGAGGGTCGTATGGTAAATGGAAAGCCG

AAAAAGATTCGCAAGCCGAGGACAATCTACTCTTCCCCGCCATCGCCAGCTGTCTGGGAC

AACAGCAGCACCCCTCAGAACACCCCGATCAGCAGACCTCAGGTGCCGCAGCCGACGCAC

AGTTCATCGCCGCCGTACCTGGAGGATTATAATAACCACTGGTACCAGCAGGGATCACAC

CTACAACACCCGGGAGCCGTGCACCACCCAGTCCCGCAGCAAAGCGTGGGAGCTGTTTAT

>dlx3b_Lepelo

GACTCCCCTACTCTGCCCGAGTCGTCCGTGACGGACATGGGCTACTACAGTGGACAGACG

GCCCACGGCCATCATGAATATTATCAGAGTCAGCCGTACGGGCAGCCCATGAACTCTTAC

CATCACCAGTTTAATCTGAACGGAATGGGAGCTGCTGGAGCGTACGCCACCAAATCTGAA

TACCCATACACGGTGAAAGAAGAGCCAGAGCCGGAGGTTCGTATGGTAAATGGAAAGCCG

AAAAAGATTCGCAAGCCGAGGACGATCTACTCTTCCCCGCCATCGCCCGCAGTCTGGGAC

AACAGCAGCACCCCTCAGAACACCCCGATCAGCAGACCTCAGGTGCCGCAGCCGACGCAC

AGTTCATCGCCGCCGTACCTGGAGGATTATAATAACCACTGGTACCAGCAGGGATCACAC

CTACAACACCCGGGAGCCGTGCACCACCCAGTCCCGCAGCAAAGCGTGGGAGCTGTTTAT

>dlx3b_Limsta

GACTCCCCTACTCTGCCCGAGTCATCCGTGACGGACATGGGCTACTACAGTGGACAGACG

GCCCACGGCCATCATGAATATTATCAGAGTCAGCCGTACGGGCAGCCCATGAACTCTTAC

CATCACCAGTTTAATCTGAACGGAATGGGAGCTGCTGGAGCGTACGCCACCAAATCTGAA

TACCCATACACGGTGAAAGAAGAGCCAGAGCCGGAGGTTCGTATGGTAAATGGAAAGCCG

AAAAAGATTCGCAAGCCGAGGACGATCTACTCTTCCCCGCCATCACCCGCAGTCTGGGAC

AACAGCAGCACCCCTCAGAACACCCCGATCAGCAGACCTCAGGTGCCGCAGCCGACGCAC

AGTTCATCGCCGCCGTACCTGGAGGATTATAATAACCACTGGTACCAGCAGGGATCACAC

CTACAACACCCGGGAGCCGTGCACCACCCAGTCCCGCAGCAAAGCGTGGGAGCTGTTTAT

>dlx3b_Loblab

GACTCCCCTACTCTGCCCGAATCGTCCGTGACGGACATGGGCTACTACAGTGGACAGACG

GCCCACGGCCATCATGAATATTATCAGAGTCAGCCGTACGGGCAGCCCATGAACTCTTAC

CATCACCAGTTTAATCTGAACGGAATGGGAGCTGCTGGAGCGTACGCCACCAAATCTGAA

TACCCATACACGGTGAAAGAAGAGCCAGAGC-----------------------------

---------------------------------TCCCCGCCATCGCCCGCTGTCTGGGAC

AACAGCAGCACCCCTCAGAACACCCCGATCAGCAGACCTCAGGTGCCGCAGCCGACGCAC

AGTTCATCGCCGCCGTACCTGGAGGATTATAATAACCACTGGTACCAGCAGGGATCACAC

CTACAACACCCGGGAGCCGTGCACCACCCAGTCCCGCAGCAAAGCGTGGGAGCTGTTTAT

>dlx3b_Neopul

GACTCCCCTACTCTGCCCGAGTCGTCCGTGACGGACATGGGCTACTACAGTGGACAGACG

GCCCACGGCCATCATGAATATTATCAGAGTCAGCCGTACGGGCAGCCCATGAACTCTTAC

CATCACCAGTTTAATCTGAACGGAATGGGAGCTGCTGGAGCGTACGCCACCAAATCTGAA

TACCCATACACGGTGAAAGA----------------------------------------

---------------------------------TCCCCGCCATCGCCCGCAGTCTGGGAC

AACAACAGCACCCCTCAGAACACCCCGATCAGCAGACCTCAGGTGCCGCAGCCGACGCAC

AGTTCATCGCCGCCGTACCTGGAGGATTATAATAACCACTGGTACCAGCAGGGATCACAC

CTACAACACCCGGGAGCCGTGCACCACCCAGTCCCGCAGCAAAGCGTGGGAGCTGTTTAT

>dlx3b_Petfam

GACTCCCCTACTCTGCCCGAGTCGTCCGTGACGGACATGGGCTACTACAGTGGACAGACG

GCCCACGGCCATCATGAATATTATCAGAGTCAGCCGTACGGGCAGCCCATGAACTCTTAC

CATCACCAGTTTAATCTGAACGGAATGGGAGCTGCTGGAGCGTACGCCACCAAATCTGAA

TACCCATACACGGTGAAAGAAGAGCCAGAGCCGGAGGTTCGTATGGTAAATGGAAAGCCG

AAAAAGATTCGCAAGCCGAGGACGATC------TCCCCGCCATCGCCCGCTGTCTGGGAC

AACAGCAGCACCCCTCAGAACACCCCGATCAGCAGACCTCAGGTGCCGCAGCCGACGCAC

AGTTCATCGCCGCCGTACCTGGAGGATTATAATAACCACTGGTACCAGCAGGGATCACAC

CTACAACACCCGGGAGCCGTGCACCACCCAGTCCCGCAGCAAAGCGTGGGAGCTGTTTAT

>dlx3b_Plesta

GACTCCCCTACTCTGCCCGAGTCGTCCGTGACGGACATGGGCTACTACAGTGGACAGACG

GCCCACGGCCATCATGAATATTATCAGAGTCAGCCGTACGGGCAGCCCATGAACTCTTAC

CATCACCAGTTTAATCTGAACGGAATGGGAGCTGCTGGAGCGTACGCCACCAAATCTGAA

TACCCATACACGGTGAAAGAAGAGCCAGAGCCGGAGGTTCGTATGGTAAATGGAAAACCG

AAAAAGATTCGCAAGCCGAGGACGATCTA----TCCCCGCCATCGCCCGCTGTCTGGGAC

AACAGCAGCACCCCTCAGAACACCCCGATCAGCAGACCTCAGGTGCCGCAGCCGACGCAC

AGTTCATCGCCGCCGTACCTGGAGGATTATAATAACCACTGGTACCAGCAGGGATCACAC

CTACAACACCCGGGAGCCGTGCACCACCCAGTCCCGCAGCAAAGCGTGGGAGCTGTTTAT

>dlx3b_Psecur

GACTCCCCTACTCTGCCCGAGTCGTCCGTGACGGACATGGGCTACTACAGTGGACAGACG

GCCCACAGCCATCATGAATATTATCAGAGTCAGCCGTACGGGCAGCCCATGAACTCTTAC

CATCACCAGTTTAATCTGAACGGAATGGGAGCTGCTGGAGCGTACGCCACCAAATCTGAA

TACCCATACACGGTGAAAGAAGAGCCAGAGCCGGAGGTTCGTATGGTAAATGGAAAGCCG

AAAAAGATTCGCAAGCCGAGGACGATCTACTCTTCCCCGCCATCGCCCGCTGTCTGGGAC

AACAGCAGCACCCCTCAGAACACCCCGATCAGCAGACCTCAGGTGCCGCAGCCGACGCAC

AGTTCATCGCCGCCGTACCTGGAGGATTATAATAACCACTGGTACCAGCAGGGATCACAC

CTACAACACCCGGGAGCCGTGCACCACCCAGTCCCGCAGCAAAGCGTGGGAGCTGTTTAT

>dlx3b_Tromoo

GACTCCCCTACTCTGCCCGAGTCGTCCGTGACGGACATGGGCTACTACAGTGGACAGACG

GCCCACGGCCATCATGAATATTATCAGAGTCAGCCGTACGGGCAGCCCATGAACTCTTAC

CATCACCAGTTTAATCTGAACGGAATGGGAGCTGCTGGAGCGTACGCCACCAAATCTGAA

TACCCATACACGGTGAAAGAAGAGCCAGAGCCGGAGGTTCGTATGGTAAATGGAAAGCCG

AAAAAGATTCGCAAGCCGAGGACGATCTACTCTTCCCCGCCATCGCCCGCTGTCTGGGAC

AACAGCAGCACCCCTCAGAACACCCCGATCAGCAGACCTCAGGTGCCGCAGCCGACGCAC

AGTTCATCGCCGCCGTACCTGGAGGATTATAATAACCACTGGTACCAGCAGGGATCACAC

CTACAACACCCGGGAGCCGTGCACCACCCAGTCCCGCAGCAAAGCGTGGGAGCTGTTTAT

>dlx3b_Tylpol

GACTCCCCTACTCTGCCCGAGTCGTCCGTGACGGACATGGGCTACTACAGTGGACAGACG

GCCCACGGCCATCATGAATATTATCAGAGTCAGCCGTACGGGCAGCCCATGAACTCTTAC

CATCACCAGTTTAATCTGAACGGAATGGGAGCTGCTGGAGCGTACGCCACCAAATCTGAA

TACCCATACACGGTGAAAGAAGAGCCAGAGCCGGAGGTTCGTATGGTAAATGGAAAGCCG

AAAAAGATTCGCAAGCCGAGGACGATCTACTCTTCCCCGCCATCGCCCGCTGTCTGGGAC

AACAGCAGCACCCCTCAGAACACCCCGATCAGCAGACCTCAGGTGCCGCAGCCGACGCAC

AGTTCATCGCCGCCGTACCTGGAGGATTATAATAACCACTGGTACCAGCAGGGATCACAC

CTACAACACCCGGGAGCCGTGCACCACCCAGTCCCGCAGCAAAGCGTGGGAGCTGTTTAT

>dlx3b_Neofur

------------------------------------------------------------

---------CATCATGAATATTATCAGAGTCAGCCGTACGGGCAGCCCATGAACTCTTAC

CATCACCAGTTTAATCTGAACGGAATGGGAGCTGCTGGAGCGTACGCCACCAAATCTGAA

TACCCATACACGGTGAAAGAAGAG------------------------------------

---------------------------------TCCCCGCCATCGCCCGCAGTCTGGGAC

AACAGCAGCACCCCTCAGAACACCCCGATCAGCAGACCTCAGGTGCCGCAGCCGACGCAC

AGTTCATCGCCGCCGTACCTGGAGGATTATAATAACCACTGGTACCAGCAGGGATCACAC

CTACAACACCCGGGAGCCGTGCACCACCCAGTCCCGCAGCAAAGCGTGGGAGCTGTTTAT

>dlx3b_Batgra

------------------------------------------------------------

------------------------------------------------------------

------------------------------------------------------------

------------------------------------------------------------

---------------------------------TCCCCGCCATCGCCCGCGGTCTGGGAC

AACAGCAGCACCCCTCAGAACACCCCGATCAGCAGACCTCAGGTGCCGCAGCCGACGCAC

AGTTCATCGCCGCCGTACCTGGAGGATTATAATAACCACTGGTACCAGCAGGGATCACAC

CTACAACACCCGGGAGCCGTGCACCACCCAGTCCCGCAGCAAAGCGTGGGAGCTGTTTAT

>dlx3b_Gnaper

------------------------------------------------------------

------------------------------------------------------------

------------------------------------------------------------

------------------------------------------------------------

---------------------------------TCCCCGCCATCACCCGCAGTCTGGGAC

AACAGCAGCACCCCTCAGAACACCCCGATCAGCAGACCTCAGGTGCCGCAGCCGACGCAC

AGTTCATCGCCGCCGTACCTGGAGGATTATAATAACCACTGGTACCAGCAGGGATCACAC

CTACAACACCCGGGAGCCGTGCACCACCCAGTCCCGCAGCAAAGCGTGGGAGCTGTTTAT

>dlx3b_Oretan

------------------------------------------------------------

------------------------------------------------------------

------------------------------------------------------------

------------------------------------------------------------

---------------------------------TCCCCGCCGTCGCCCGCAGTCTGGGAC

AACAGCAGCACCCCTCAGAACACCCCGATCAGCAGACCTCAGGTGCCGCAGCCGACGCAC

AGTTCATCGCCGCCGTACCTGGAGGATTATAATAACCACTGGTACCAGCAGGGATCACAC

CTACAGCACCCGGGAACCGTTCACCACCCAGTCCCGCAGCAAAGCGTGGGAGCTGTTTAT

>dlx3b_Permic

------------------------------------------------------------

------------------------------------------------------------

------------------------------------------------------------

------------------------------------------------------------

---------------------------------TCCCCGCCATCGCCCGCTGTCTGGGAC

AACAGCAGCACCCCTCAGAACACCCCGATCAGCAGACCTCAGGTGCCGCAGCCGACGCAC

AGTTCATCGCCGCCGTACCTGGAGGATTATAATAACCACTGGTACCAGCAGGGATCACAC

CTACAACACCCGGGAGCCGTGCACCACCCAGTCCCGCAGCAAAGCGTGGGAGCTGTTTAT

>dlx3b_Varmoo

------------------------------------------------------------

------------------------------------------------------------

------------------------------------------------------------

------------------------------------------------------------

---------------------------------TCCCCGCCATCGCCCGCAGTCTGGGAC

AACAGCAGCACCCCTCAGAACACCCCGATCAGCAGACCTCAGGTGCCGCAGCCGACGCAC

AGTTCATCGCCGCCGTACCTGGAGGATTATAATAACCACTGGTACCAGCAGGGATCACAC

CTACAACACCCGGGAGCCGTGCACCACCCAGTCCCGCAGCAAAGCGTGGGAGCTGTTTAT

Dlx4a input file:

>dlx4a_Altfas

ATGACTATGAGCTCCATATCAGACACTTTAGTAACGTCCGATCCGTCCAAATCTGCGTTT

TTGGAGTTCGGTGGACACAGCTACCCTGGACACCCTGGACATCAACAACCTTCGCCAGGC

TTATCCCACAACCATTATCCGGTCCACGGACTGCACGCTGTCGGACCCTCGCAGCACGAT

GGGCCCTTCTCCTCCGGGGCTTCCTCTTACGGTCGCCCGCTGGTGAACAATTTTGGACAC

TGGTATCCCGGACACCAGCAGGAACCCATGCCAAGGACTCAGATGATG

>dlx4a_Batgra

ATGACTATGAGCTCCATATCAGACACTTTAGTAACGTCCGATCCGTCCAAATCTGCGTTT

TTGGAGTTCGGTGGACACAGCTACCCTGGACACCCTGGACATCAACAACCTTCGCCAGGC

TTATCCCACAACCATTATCCGGTCCACGGATTGCACGCTGTCGGACCCTCGCAGCACGAT

GGGCCCTTCTCCTCCGGGGCTTCCTCTTACGGTCGCCCGCTGGTGAACAATTTTGGACAC

TGGTATCCCGGACACCAGCAGGAGCCCATGCCAAGAACTCAGATGATG

>dlx4a_Boumic

ATGACTATGAGCTCCATATCAGACACTTTAGTAACGTCCGATCCGTCCAAATCTGCGTTT

TTGGAGTTCGGTGGACACAGCTACCCTGGACACCCTGGACATCAACAACCTTCGCCAGGC

TTATCCCACAACCATTATCCGGTCCACGGACTGCACGCTGTCGGACCCTCGCAGCACGAT

GGGCCCTTCTCCTCCGGGGCTTCCTCTTACGGTCGCCCGCTGGTGAACAATTTTGGACAC

TGGTATCCTGGACACCAGCAGGAACCCATGCCAAGAACTCAGATGATG

>dlx4a_Ctehor

ATGACTATGAGCTCCATATCAGACACTTTAGTAACGTCCGATCCGTCCAAATCTGCGTTT

TTGGAGTTCGGTGGACACAGCTACCCTGGACACCCTGGACATCAACAACCTTCGCCAGGC

TTATCCCACAACCATTATCCGGTCCACGGACTGCACGCTGTCGGACCCTCGCAGCACGAT

GGGCCCTTCTCCTCCGGGGCTTCCTCTTACGGTCGCCCGCTGGTGAACAATTTTGGACAC

TGGTATCCCGGACACCAGCAGGAACCCATGCCAAGAACTCAGATGATG

>dlx4a_Cyafur

ATGACTATGAGCTCCATATCAGACACTTTAGTAACGTCCGATCCGTCCAAATCTGCGTTT

TTGGAGTTCGGTGGACACAGCTACCCTGGACACCCTGGACATCAACAACCTTCGCCAGGC

TTATCCCACAACCATTATCCGGTCCACGGACTGCACGCTGTCGGACCCTCGCAGCACGAT

GGGCCCTTCTCCTCCGGGGCTTCCTCTTACGGTCGCCCGCTGGTGAACAATTTTGGACAC

TGGTATCCCGGACACCAGCAGGAACCCATGCCAAGAACTCAGATGATG

>dlx4a_Cypfro

ATGACTATGAGCTCCATATCAGACACTTTAGTAACGTCCGATCCGTCCAAATCTGCGTTT

TTGGAGTTCGGTGGACACAGCTACCCTGGACACCCTGGACATCAACAACCTTCGCCAGGC

TTATCCCACAACCATTATCCGGTCCACGGACTGCACGCTGTCGGACCCTCGCAGCACGAT

GGGCCCTTCTCCTCCGGGGCTTCCTCTTACGGTCGCCCGCTGGTGAACAATTTTGGACAC

TGGTATCCCGGACACCAGCAGGAACCCATGCCAAGAACTCAGATGATG

>dlx4a_Cyplep

ATGACTATGAGCTCCATATCAGACACTTTAGTAACGTCCGATCCGTCCAAATCTGCGTTT

TTGGAGTTCGGTGGACACAGCTACCCTGGACACCCTGGACATCAACAACTTTCGCCAGGC

TTATCCCACAACCATTATCCGGTCCACGGACTGCACGCTGTCGGACCCTCGCAGCACGAT

GGGCCCTTCTCTTCCGGGGCTTCCTCTTACGGTCGCCCGCTGGTGAACAATTTTGGACAC

TGGTATCCCGGACACCAGCAGGAACCCATGCCAAGAACTCAGATGATG

>dlx4a_Gnaper

ATGACTATGAGCTCCATATCAGACACTTTAGTAACGTCCGATCCGTCCAAATCTGCGTTT

TTGGAGTTCGGTGGACACAGCTACCCTGGACACCCTGGACATCAACAACCTTCGCCAGGC

TTATCCCACAACCATTATCCGGTCCACGGACTGCACGCTGTCGGACCCTCGCAGCACGAT

GGGCCCTTCTCCTCCGGGGCTTCCTCTTACGGTCGCCCGCTGGTGAACAATTTTGGACAC

TGGTATCCCGGACACCAGCAGGAACCCATGCCAAGAACTCAGATGATG

>dlx4a_Lepelo

ATGACTATGAGCTCCATATCAGACACTTTAGTAACGTCCGATCCGTCCAAATCTGCGTTT

TTGGAGTTCGGTGGACACAGCTACCCTGGACACCCTGGACATCAACAACCTTCGCCAGGC

TTATCCCACAACCATTATCCGGTCCACGGACTGCACGCTGTCGGACCCTCGCAGCACGAT

GGGCCCTTCTCCTCCGGGGCTTCCTCTTACGGTCGCCCGCTGGTGAACAATTTTGGACAC

TGGTATCCCGGACACCAGCAGGAACCCATGCCAAGGACTCAGATGATG

>dlx4a_Limsta

ATGACTATGAGCTCCATATCAGACACTTTAGTAACGTCCGATCCGTCCAAATCTGCGTTT

TTGGAGTTCGGTGGACACAGCTACCCTGGACACCCTGGACATCAACAACCTTCGCCAGGC

TTATCCCACAACCATTATCCGGTCCACGGACTGCACGCTGTCGGACCCTCGCAGCACGAT

GGGCCCTTCTCCTCCGGGGCTTCCTCTTACGGTCGCCCGCTGGTGAACAATTTTGGACAC

TGGTATCCCGGACACCAGCAGGAACCCATGCCAAGAACTCAGATGATG

>dlx4a_Loblab

ATGACTATGAGCTCCATATCAGACACTTTAGTAACGTCCGATCCGTCCAAATCTGCGTTT

TTGGAGTTCGGTGGACACAGCTACCCTGGACACCCTGGACATCAACAACCTTCGCCAGGC

TTATCCCACAACCATTATCCGGTCCACGGACTGCACGCTGTCGGACCCTCGCAGCACGAT

GGGCCCTTCTCCTCCGGGGCTTCCTCTTACGGTCGCCCGCTGGTGAACAATTTTGGACAC

TGGTATCCCGGACACCAGCAGGAACCCATGCCAAGAACTCAGATGATG

>dlx4a_Neofur

ATGACTATGAGCTCCATATCAGACACTTTAGTAACGTCCGATCCGTCCAAATCTGCGTTT

TTGGAGTTCGGTGGACACAGCTACCCTGGACACCCTGGACATCAACAACCTTCGCCAGGC

TTATCCCACAACCATTATCCGGTCCACGGACTGCACGCTGTCGGACCCTCGCAGCACGAT

GGGCCCTTCTCCTCCGGGGCTTCCTCTTACGGTCGCCCGCTGGTGAACAATTTTGGACAC

TGGTATCCCGGACACCAGCAGGAACCCATGCCAAGAACTCAGATGATG

>dlx4a_Neopul

ATGACTATGAGCTCCATATCAGACACTTTAGTAACGTCCGATCCGTCCAAATCTGCGTTT

TTGGAGTTCGGTGGACACAGCTACCCTGGACACCCTGGACATCAACAACCTTCGCCAGGC

TTATCCCACAACCATTATCCGGTCCACGGACTGCACGCTGTCGGACCCTCGCAGCACGAT

GGGCCCTTCTCCTCCGGGGCTTCCTCTTACGGTCGCCCGCTGGTGAACAATTTTGGACAC

TGGTATCCCGGACACCAGCAGGAACCCATGCCAAGGACTCAGATGATG

>dlx4a_Oretan

ATGACTATGAGCTCCATATCAGACACTTTAGTAACGTCCGATCCGTCCAAATCTGCGTTT

TTGGAGTTCGGTGGACACAGCTACCCTGGACACCCTGGACATCAACAACCTTCGCCAGGC

TTATCCCACAACCATTATCCGGTCCACGGACTGCACGCTGTCGGGCCCTCGCAGCACGAT

GGGCCCTTCTCCTCCGGTGCTTCCTCTTACGGTCGCCCGCTGGTGAACAATTTTGGACAC

TGGTATCCCGGACACCAGCAGGAACCCATGCCAAGAACTCAGATGATG

>dlx4a_Permic

ATGACTATGAGCTCCATATCAGACACTTTAGTAACGTCCGATCCGTCCAAATCTGCGTTT

TTGGAGTTCGGTGGACACAGCTACCCTGGACACCCTGGACATCAACAACCTTCGCCAGGC

TTATCCCACAACCATTATCCGGTCCACGGACTGCACGCTGTCGGACCCTCGCAGCACGAT

GGGCCCTTCTCCTCCGGGGCTTCCTCTTACGGTCGCCCGCTGGTGAACAATTTTGGACAC

TGGTATCCCGGACACCAGCAGGAACCCATGCCAAGAACTCAGATGATG

>dlx4a_Petfam

ATGACTATGAGCTCCATATCAGACACTTTAGTAACGTCCGATCCGTCCAAATCTGCGTTT

TTGGAGTTCGGTGGACACAGCTACCCTGGACACCCTGGACATCAACAACCTTCGCCAGGC

TTATCCCACAACCATTATCCGGTCCACGGACTGCACGCTGTCGGACCCTCGCAGCACGAT

GGGCCCTTCTCCTCCGGGGCTTCCTCTTACGGTCGCCCGCTGGTGAACAATTTTGGACAC

TGGTATCCCGGACACCAGCAGGAACCCATGCCAAGAACTCAGATGATG

>dlx4a_Plestr

ATGACTATGAGCTCCATATCAGACACTTTAGTAACGTCCGATCCGTCCAAATCTGCGTTT

TTGGAGTTCGGTGGACACAGCTACCCTGGACACCCTGGACATCAACAACCTTCGCCAGGC

TTATCCCACAACCATTATCCGGTCCACGGACTGCACGCTGTCGGACCCTCGCAGCACGAT

GGGCCCTTCTCCTCCGGGGCTTCCTCTTACGGTCGCCCGCTGGTGAACAATTTTGGACAC

TGGTATCCCGGACACCAGCAGGAACCCATGCCAAGAACTCAGATGATG

>dlx4a_Psecur

ATGACTATGAGCTCCATATCAGACACTTTAGTAACGTCCGATCCGTCCAAATCTGCGTTT

TTGGAGTTTGGTGGACACAGCTACCCTGGACACCCTGGACATCAACAACCTTCGCCAGGC

TTATCCCACAACCATTATCCGGTCCACGGACTGCACGCTGTCGGACCCTCGCAGCACGAT

GGGCCCTTCTCCTCCGGGGCTTCCTCTTACGGTCGCCCGCTGGTGAACAATTTTGGACAC

TGGTATCCCGGACACCAGCAGGAACCCATGCCAAGAACTCAGATGATG

>dlx4a_Tromoo

ATGACTATGAGCTCCATATCAGACACTTTAGTAACGTCCGATCCGTCCAAATCTGCGTTT

TTGGAGTTCGGTGGACACAGCTACCCTGGACACCCTGGACATCAACAACCTTCGCCAGGC

TTATCCCACAACCATTATCCGGTCCACGGACTGCACGCTGTCGGACCCTCGCAGCACGAT

GGGCCCTTCTCCTCCGGGGCTTCCTCTTACGGTCGCCCGCTGGTGAACAATTTTGGACAC

TGGTATCCCGGACACCAGCAGGAACCCATGCCAAGAACTCAGATGATG

>dlx4a_Tylpol

ATGACTATGAGCTCCATATCAGACACTTTAGTAACGTCCGATCCGTCCAAATCTGCGTTT

TTGGAGTTCGGTGGACACAGCTACCCTGGACACCCTGGACATCAACAACCTTCGCCAGGC

TTATCCCACAACCATTATCCGGTCCACGGACTGCACGCTGTCGGACCCTCGCAGCACGAT

GGGCCCTTCTCCTCCGGGGCTTCCTCTTACGGTCGCCCGCTGGTGAACAATTTTGGACAC

TGGTATCCCGGACACCAGCAGGAACCCATGCCAAGAACTCAGATGATG

>dlx4a_Varmoo

ATGACTATGAGCTCCATATCAGACACTTTAGTAACGTCCGATCCGTCCAAATCTGCGTTT

TTGGAGTTCGGTGGACACAGCTACCCTGGACACCCTGGACATCAACAACCTTCGCCAGGC

TTATCCCACAACCATTATCCGGTCCACGGACTGCACGCTGTCGGACCCTCGCAGCACGAT

GGGCCCTTCTCCTCCGGGGCTTCCTCTTACGGTCGCCCGCTGGTGAACAATTTTGGACAC

TGGTATCCCGGACACCAGCAGGAACCCATGCCAAGGACTCAGATGATG

>dlx4a_Astbur

------------------------------------------------------------

------------------------------------------------------------

------------------------------------------------------------

-------------------------------------------TGAACAATTTTGGACAC

TGGTATCCCGGACACCAGCAGGAACCCATGCCAAGAACTCAGATGATG

Dlx4b input file:

>dlx4b_Astbur

ATGATGTCTGTGGGTTTTATCCCTGACAGTCTGAATGGCTCAGATCCCTCCAAGTCGGCC

TTTCTAGAATTCGGACACGGACACTCGACGCACCAGCAGCACTCCTCCGGACTCTCTCAC

GTTTATCCGGTTCACGGCTTGCAAGCTGCTGGCCATTCCCAGCACGAAAGCCCTTTTCCT

GGTACCGCGTCCTATGGCCGCTCTTTGGGCTACGCCTACCCCGGCACGGTGAACACTCAT

CCCCCGTCCGCTTACATGCCCTACCAACA-CGCTCCAAGTACAAAAAGATCATGAAGCAT

GGCAGCGGATCAGAGGGAGAACATCTCCACGGCACCAGCTCCATCTCTCCCTGCTCGCCT

GCATTGCCCCAGCTATGGGAGGTCTCTATGGCGAACAAAGGAGCCCCGGTCCATCCGAGC

AGTTACATGAACACTTTCGGTCATTGGTATCCGAATCATCACCCTCATCATCAGGACGCG

ATGCACAGGCCTCAGATGATG

>dlx4b_Altfas

------------------------------------------------------------

------------------------------------------------------------

------------------------------------------------------------

------------------------------------------------------------

------------------------------CGCTCCAAGTACAAAAAGATCATGAAGCAT

GGCAGCGGATCAGAGGGAGAACATCTCCACGGCACCAGCTCCATCTCTCCCTGCTCGCCC

GCATTGCCACAGCTATGGGAGGTCTCCATGGCGAACAAAGGAGGCCCGGTCCATCCGAGC

AGTTACATGAACAGTTTCGGTCACTGGTATCCGAATCATCACCCTCATCATCAGGACGCG

ATGCACAGGCCTCAGATGATG

>dlx4b_Batgra

ATGATGTCTGTGGGTTTTATCCCTGACAGTTTGAATGGATCAGATCCCTCCAAGTCGGCC

TTTCTAGAATTCGGACACGGACACTCGACGCACCAGCAGCACTCCTCCGGACTCTCTCAC

GTTTATCCGGTTCACGGCTTGCACGCTGCTGGCCATTCCCAGCACGAAAGCCCTTTTCCT

GGTACCGCGTCCTATGGCCGCTCTTTGGGCTACGCCTACCCCGGCACGGTGAACACTCAT

CCCCCGTCCGCTTACATGCCCTACCAACAC------------------------------

------------------------------------------------------------

------------------------------------------------------------

------------------------------------------------------------

---------------------

>dlx4b_Boumic

ATGATGTCTGTGGGTTTTATCCCTGACAGTCTGAATGGCTCAGATCCCTCCAAGTCGGCC

TTTCTAGAATTCGGACACGGACACTCGACGCACCAGCAGCACTCCTCCGGACTCTCTCAC

GTTTATCCGGTTCACGGCTTGCACGCTGCTGGCCATTCCCAGCACGAAAGCCCTTTTCCT

GGTACCGCGTCCTATGGCCGCTCTTTGGGCTACGCCTACCCCGGCACGGTGAACACTCAT

CCCCCGTCCGCTTACATGCCCTACCAACACCGCTCCAAGTACAAAAAGATCATGAAGCAT

GGCAGCGGATCAGAGGGAGAACATCTCCACGGCACCAGCTCCATCTCTCCCTGCTCGCCC

GCATTGCCCCAGCTATGGGAGGTCTCCATGGCGAACAAAGGAGCCCCGGTCCATCCGAGC

AGTTACATGAACAGTTTCGGTCACTGGTATCCGAATCATCACCCTCATCATCAGGACGCG

ATGCACAGGCCTCAGATGATG

>dlx4b_Cyafur

ATGATGTCTGTGGGTTTTATCCCTGACAGTCTGAATGGCTCAGATCCCTCCAAGTCGGCC

TTTCTAGAATTCGGACACGGACACTCGACGCACCAGCAGCACTCCTCCGGACTCTCTCAC

GTTTATCCGGTTCACGGCTTGCAAGCTGCTGGCCATTCCCAGCACGAAAGCCCTTTTCCT

GGTACCGCGTCCTATGGCCGCTCTTTGGGCTACGCCTACCCCGGCACGGTGAACACTCAT

CCCCAGTCCGCTTACATGCCCTACCAACA-CGCTCCAAGTACAAAAAGATCATGAAGCAT

GGCAGCGGATCAGAGGGAGAACATCTCCACGGCACCAGCTCCATCTCTCCCTGCTCGCCC

GCATTGCCCCAGCTATGGGAGGTCTCCATGGCGAACAAAGGAGCCCCGGTCCATCCGAGC

AGTTATATGAACAGTTTCGGTCACTGGTATCCGAATCATCACCCTCATCATCAGGACGCG

ATGCACAGGCCTCAGATGATG

>dlx4b_Cypfro

ATGATGTCTGTGGGTTTTATCCCTGACAGTCTGAATGGCTCAGATCCCTCCAAGTCGGCC

TTTCTAGAATTCGGACACGGACACTCGACGCACCAGCAGCACTCCTCCGGACTCTCTCAC

GTTTATCCGGTTCACGGCTTGCAAGCTGCTGGCCATTCCCAGCACGAAAGCCCTTTTCCT

GGTACCGCGTCCTATGGCCGCTCTTTGGGCTACGCCTACCCCGGCACGGTGAACACTCAT

CCCCCGTCCGCTTACATGCCCTACCAACACCGCTCCAAGTACAAAAAGATCATGAAGCAT

GGCAGCGGATCAGAGGGAGAACATCTCCACGGCACCAGCTCCATCTCTCCCTGCTCGCCC

GCATTGCCCCAGCTATGGGAGGTCTCCATGGCGAACAAAGGAGCCCCGGTCCATCCGAGC

AGTTACATGAACACTTTCGGTCACTGGTATCCGAATCATCACCCTCATCATCAGGACGCG

ATGCACAGGCCTCAGATGATG

>dlx4b_Cyplep

ATGATGTCTGTGGGTTTTATCCCTGACAGTCTGAATGGCTCAGATCCCTCCAAGTCGGCC

TTTCTAGAATTCGGATACGGACACTCGACGCACCAGCAGCACTCCTCCGGACTCTCTCAC

GTTTATCCGGTTCACGGCTTGCAAGCTGCTGGCCATTCCCAGCACGAAAGCCCTTTTCCT

GGTACCGCGTCCTATGGCCGCTCTTTGGGCTACGCCTACCCCGGCACGGTGAACACTCAT

CCCCCGTCCGCTTACATGCCCTACCAACACCGCTCCAAGTACAAAAAGATCATGAAGCAT

GGCAGCGGATCAGAGGGAGAACATCTCCACGGCACCAGCTCCATCTCTCCCTGCTCGCCC

GCATTGCCCCAGCTATGGGAGGTCTCCATGGCGAACAAAGGAGCCCCGGTCCATCCGAGC

AGTTACATGAACACTTTCGGTCACTGGTATCCGAATCATCACCCTCATCATCAGGACGCG

ATGCACAGGCCTCAGATGATG

>dlx4b_Limsta

ATGATGTCTGTGGGTTTTATCCCTGACAGTCTGAATGGCTCAGATCCCTCCAAGTCGGCC

TTTCTAGAATTCGGACACGGACACTCGACGCACCAGCAGCACTCCTCCGGACTCTCTCAC

GTTTATCCGGTTCACGGCTTGCAAGCTGCTGGCCATTCCCAGCACGAAAGCCCTTTTCCT

GGTACCGCGTCCTATGGCCGCTCTTTGGGCTACGCCTACCCCGGCACGGTGAACACTCAT

CCCCCGTCCGCTTACATGCCCTACCAACACCGCTCCAAGTACAAAAAGATCATGAAGCAT

GGCAGCGGATCAGAGGGAGAACATCTCCACGGCACCAGCTCCATCTCTCCCTGCTCGCCC

GCATTGCCCCAGCTATGGGAGGTCTCCATGGCGAACAAAGGAGCCCCGGTCCATCCGAGC

AGTTACATGAACACTTTCGGTCACTGGTATCCGAATCATCACCCTCATCATCAGGACGCG

ATGCACAGGCCTCAGATGATG

>dlx4b_Permic

ATGATGTCTGTGGGTTTTATCCCTGACAGTCTGAATGGCTCAGATCCCTCCAAGTCGGCC

TTTCTAGAATTCGGACACGGACACTCGACGCACCAGCAGCACTCCTCCGGACTCTCTCAC

GTTTATCCGGTTCACGGCTTGCAAGCTGCTGGCCATTCCCAGCACGAAAGCCCTTTTCCT

GGTACCGCGTCCTATGGCCGCTCTTTGGGCTACGCCTACCCCGGCACGGTGAACACTCAT

CCCCCGTCCGCTTACATGCCCTACCAACACCGCTCCAAGTACAAAAAGATCATGAAGCAT

GGCAGCGGATCAGAGGGAGAACATCTCCACGGCACCAGCTCCATCTCTCCCTGCTCGCCC

GCATTGCCCCAGCTATGGGAGGTCTCCATGGCGAACAAAGGAGCCCCGGTCCATCCGAGC

AGTTACATGAACACTTTCGGTCATTGGTATCCGAATCATCACCCTCATCATCAGGACGCG

ATGCACAGGCCTCAGATGATG

>dlx4b_Plestr

ATGATGTCTGTGGGTTTTATCCCTGACAGTCTGAATGGCTCAGATCCCTCCAAGTCGGCC

TTTCTAGAATTCGGACACGGACACTCGACGCACCAGCAGCACTCCTCCGGACTCTCTCAC

GTTTATCCGGTTCACGGCTTGCAAGCTGCTGGCCATTCCCAGCACGAAAGCCCTTTTCCT

GGTACCGCGTCCTATGGCCGCTCTTTGGGCTACGCCTACCCCGGCACGGTGAACACTCAT

CCCCCGTCCGCTTACATGCCCTACCAACACCGCTCCAAGTACAAAAAGATCATGAAGCAT

GGCAGCGGATCAGAGGGAGAACATCTCCACGGCACCAGCTCCATCTCTCCCTGCTCGCCC

GCATTGCCCCAGCTATGGGAGGTCTCCATGGCGAACAAAGGAGCCCCGGTCCATCCGAGC

AGTTACATGAACACTTTCGGTCATTGGTATCCGAATCATCACCCTCATCATCAGGACGCG

ATGCACAGGCCTCAGATGATG

>dlx4b_Lepelo

ATGATGTCTGTGGGTTTTATCCCTGACAGTCTGAATGGCTCAGATCCCTCCAAGTCGGCC

TTTCTAGAATTCGGACACGGACACTCGACGCACCAGCAACACTCCTCCGGACTCTCTCAC

GTTTATCCGGTTCACGGCTTGCAAGCTGCTGGCCATTCCCAGCACGAAAGCCCTTTTCCT

GGTACCGCGTCCTATGGCCGCTCTTTGGGCTACGCCTACCCCGGCACGGTGAACACTCAT

CCCCCGTCCGCTTACATGCCCTACCAACACCGCTCCAAGTACAAAAAGATCATGAAGCAT

GGCAGCGGATCAGAGGGAGAACATCTCCACGGCACCAGCTCCATCTCTCCCTGCTCGCCC

GCATTGCCACAGCTATGGGAGGTCTCCATGGCGAACAAAGGAGGCCCGGTCCATCCGAGC

AGTTACATGAACAGTTTCGGTCACTGGTATCCGAATCATCACCCTCATCATCAGGACGCG

ATGCACAGGCCTCAGATGATG

>dlx4b_Neofur

ATGATGTCTGTGGGTTTTATCCCTGACAGTCTGAATGGCTCAGATCCCTCCAAGTCGGCC

TTTCTAGAATTCGGACACGGACACTCGACGCACCAGCAACACTCCTCCGGACTCTCTCAC

GTTTATCCGGTTCACGGCTTGCAAGCTGCTGGCCATTCCCAGCACGAAAGCCCTTTTCCT

GGTACCGCGTCCTATGGCCGCTCTTTGGGCTACGCCTACCCCGGCACGGTGAACACTCAT

CCCCCGTCCGCTTACATGCCCTACCAACACCGCTCCAAGTACAAAAAGATCATGAAGCAT

GGCAGCGGATCAGAGGGAGAACATCTCCACGGCACCAGCTCCATCTCTCCCTGCTCGCCC

GCATTGCCACAGCTATGGGAGGTCTCCATGGCGAACAAAGGAGGCCCGGTCCATCCGAGC

AGTTACATGAACAGTTTCGGTCACTGGTATCCGAATCATCACCCTCATCATCAGGACGCG

ATGCACAGGCCTCAGATGATG

>dlx4b_Neopul

ATGATGTCTGTGGGTTTTATCCCTGACAGTCTGAATGGCTCAGATCCCTCCAAGTCGGCC

TTTCTAGAATTCGGACACGGACACTCGACGCACCAGCAGCACTCCTCCGGACTCTCTCAC

GTTTATCCGGTTCACGGCTTGCAAGCTGCTGGCCATTCCCAGCACGAAAGCCCTTTTCCT

GGTACCGCGTCCTATGGCCGCTCTTTGGGCTACGCCTACCCCGGCACGGTGAACACTCAT

CCCCCGTCCGCTTACATGCCCTACCAACACCGCTCCAAGTACAAAAAGATCATGAAGCAT

GGCAGCGGATCAGAGGGAGAACATCTCCACGGCACCAGCTCCATCTCTCCCTGCTCGCCC

GCATTGCCACAGCTATGGGAGGTCTCCATGGCGAACAAAGGAGGCCCGGTCCATCCGAGC

AGTTACATGAACAGTTTCGGTCACTGGTATCCGAATCATCACCCTCATCATCAGGACGCG

ATGCACAGGCCTCAGATGATG

>dlx4b_Varmoo

ATGATGTCTGTGGGTTTTATCCCTGACAGTCTGAATGGCTCAGATCCCTCCAAGTCGGCC

TTTCTAGAATTCGGACACGGACACTCGACGCACCAGCAGCACTCCTCCGGACTCTCTCAC

GTTTATCCGGTTCACGGCTTGCAAGCTGCTGGCCATTCCCAGCATGAAAGCCCTTTTCCT

GGTACCGCGTCCTATGGCCGCTCTTTGGGCTACGCCTACCCCGGCACGGTGAACGCTC--

------------------------------------------------------------

------------------------------------------------------------

------------------------------------------------------------

------------------------------------------------------------

---------------------

>dlx4b_Calmac

ATGATGTCTGTGGGTTTTATCCCTGACAGTCTGAATGGCTCAGATCCCTCCAAGTCGGCC

TTTCTAGAATTCGGACACGGACACTCGACGCACCAGCAGCACTCCTCCGGACTCTCTCAC

GTTTATCCGGTTCACGGCTTGCAAGCTGCTGGCCATTCCCAGCACGAAAGCCCTTTTCCT

GGTACCGCGTCCTATGGCCGCTCTTTGGGCTACGCCTACCCCGGCACGGTGAACACTC--

------------------------------------------------------------

------------------------------------------------------------

------------------------------------------------------------

------------------------------------------------------------

---------------------

>dlx4b_Oretan

ATGATGTCTGTGGGTTTTATGCCTGACAGTCTTAATGGCTCAGATCCCTCCAAGTCGGCC

TTTCTAGAATTCGGCCACGGACACTCGACGCACCAGCAGCACTCCTCCGGACTCTCTCAC

GTTTATCCGGTTCACGGCTTGCACGCTGCTGGCCATTCCCAGCACGAAAGCCCTTTTCCT

GGTACCGCGTCCTATGGCCGCTCTCTGGGCTACGCCTACCCAGGCACGGTGAACACTCAT

CCCCCGTCCGCTTACATGCCCTACCAACACCGCTCCAAGTACAAAAAGATCATGAAGCAT

GGCAGCGGATCAGAGGGAGAACATCTCCACGGCACCAGCTCCATCTCTCCCTGCTCGCCC

GCATTGCCCCAGCTATGGGAGGTCTCCATGGCGAACAAAGGAGCCCCGGTCCATCCGAGC

AGTTACATGAACAGTTTCAGTCACTGGTATCCGAATCACCACCCTCATCATCAGGACGCG

ATGCACAGGCCTCAGATGATG

>dlx4b_Tylpol

ATGATGTCTGTGGGTTTTATCCCTGACAGTCTGAATGGCTCAGATCCCTCCAAGTCGGCC

TTTCTAGAATTCGGACACGGACACTCGACGCACCAGCAGCACTCCTCCGGACTCTCTCAC

GTTTATCCGGTTCACGGCTTGCAAGCTGCTGGCCATTCCCAGCACGAAAGCCCTTTTCCT

GGTACCGCGTCCTATGGCCGCTCTTTGGGCTACGCCTACCCCGGCACGGTGAACACTCAT

CCCCCGTCCGCTTACATGCCCTACCAACACCGCTCCAAGTACAAAAAGATCATGAAGCAT

GGCAGCGGATCAGAGGGAGAACATCTCCACGGCACCAGCTCCATCTCTCCCTGCTCGCCC

GCATTGCCCCAGCTATGGGAGGTCTCCATGGCGAACAAAGGAGCCCCGGTCCATCCGAGC

AGTTACATGAACACTTTCGGTCATTGGTATCCGAATCATCACCCTCATCATCAGGACGCG

ATGCACAGGCCTCAGATGATG

>dlx4b_Ctehor

ATGATGTCTGTGGGTTTTATCCCTGACAGTCTGAATGGCTCAGATCCCTCCAAGTCGGCC

TTTCTAGAATTCGGACACGGACACTCGACGCACCAGCAGCACTCCTCCGGACTCTCTCAC

GTTTATCCGGTTCACGGCTTGCAAGCTGCTGGCCATTCCCAGCACGAAAGCCCTTTTCCT

GGTACCGCGTCCTATGGCCGCTCTTTGGGCTACGCCTACCCCGGCACGGTGAACACTCAT

CCCCCGTCCGCTTACATGCCCTACCAACACCGCTCCAAGTACAAAAAGATCATGAAGCAT

GGCAGCGGATCAGAGGGAGAACATCTCCACGGCACCAGCTCCATCTCTCCCTGCTCGCCC

GCATTGCCCCAGCTATGGGAGGTCTCCATGGCGAACAAAGGAGCCCCGGTCCATCCGAGC

AGTTACATGAACACTTTCGGTCATTGGTATCCGAATCATCACCCTCATCATCAGGACGCG

ATGCACAGGCCTCAGATGATG

>dlx4b_Loblab

ATGATGTCTGTGGGTTTTATCCCTGACAGTCTGAATGGCTCAGATCCCTCCAAGTCGGCC

TTTCTAGAATTCGGACACGGACACTCGACGCACCAGCAGCACTCCTCCGGACTCTCTCAC

GTTTATCCGGTTCACGGCTTGCAAGCTGCTGGCCATTCCCAGCACGAAAGCCCTTTTCCT

GGTACCGCGTCCTATAGCCGCTCTTTGGGCTACGCCTACCCCGGCACGGTGAACACTCAT

CCCCCGTCCGCTTACATGCCCTACCAACACCGCTCCAAGTACAAAAAGATCATGAAGCAT

GGCAGCGGATCAGAGGGAGAACATCTCCACGGCACCAGCTCCATCTCTCCCTGCTCGCCC

GCATTGCCCCAGCTATGGGAGGTCTCTATGGCGAACAAAGGAGCCCCGGTCCATCCGAGC

AGTTACATGAACACTTTCGGTCATTGGTATCCGAATCATCACCCTCATCATCAGGACGCG

ATGCACAGGCCTCAGATGATG

>dlx4b_Petfam

ATGATGTCTGTGGGTTTTATCCCTGACAGTCTGAATGGCTCAGATCCCTCCAAGTCGGCC

TTTCTAGAATTCGGACACGGACACTCGACGCACCAGCAGCACTCCTCCGGACTCTCTCAC

GTTTATCCGGTTCACGGCTTGCAAGCTGCTGGCCATTCCCAGCACGAAAGCCCTTTTCCT

GGTACCGCGTCCTATGGCCGCTCTTTGGGCTACGCCTACCCCGGCACGGTGAACACTC--

------------------------------------------------------------

------------------------------------------------------------

------------------------------------------------------------

------------------------------------------------------------

---------------------

>dlx4b_Psecur

ATGATGTCTGTGGGTTTTATCCCTGACAGTCTGAATGGCTCAGATCCCTCCAAGTCGGCC

TTTCTAGAATTCGGACACGGACACTCGACGCACCAGCAGCACTCCTCCGGACTCTCTCAC

GTTTATCCGGTTCACGGCTTGCAAGCTGCTGGCCATTCCCAGCACGAAAGCCCTTTTCCT

GGTACCGCGTCCTATGGCCGCTCTTTGGGCTACGCCTACCCCGGCACGGTGAACACTCAT

CCCCCGTCCGCTTACATGCCCTACCAACA-------------------------------

------------------------------------------------------------

------------------------------------------------------------

------------------------------------------------------------

---------------------

>dlx4b_Tromoo

ATGATGTCTGTGGGTTTTATCCCTGACAGTTTGAATGGCTCAGATCCCTCCAAGTCGGCC

TTTCTAGAATTCGGACACGGACACTCGACGCACCAGCAGCACTCCTCCGGACTCTCTCAC

GTTTATCCGGTTCACGGCTTGCAAGCTGCTGGCCATTCCCAGCACGAAAGCCCTTTTCCT

GGTACCGCGTCCTATGGCCGCTCTTTGGGCTACGCCTACCCCGGCACGGTGAACACTC--

------------------------------------------------------------

------------------------------------------------------------

------------------------------------------------------------

------------------------------------------------------------

---------------------

>dlx4b_Gnaper

ATGATGTCTGTGGGTTTTATCCCTGACAGTCTGAATGGCTCAGATCCCTCCAAGTCGGCC

TTTCTAGAATTCGGACACGGACACTCGACGCACCAGCAGCACTCCTCCGGACTCTCTCAC

GTTTATCCGGTTCACGGCTTGCAAGCTGCTGGCCATTCCCAGCACGAAAGCCCTTTTCCT

GGTACCGCGTCCTATGGCCGCTCTTTGGGCTACGCCTACCCCGGCACGGTGAACACTCAT

CCCCCGTCCGCTTACATGCCCTACCAACACCGCTCCAAGTACAAAAAGATCATGAAGCAT

GGCAGCGGATCAGAGGGAGAACATCTCCACGGCACCAGCTCCATCTCTCCCTGCTCGCCC

GCATTGCCCCAGCTATGGGAGGTCTCCATGGCGAACAAAGGAGCCCCGGTCCATCCGAGC

AATTACATGAACACTTTCGGTCACTGGTATCCGAATCATCACCCTCATCATCAGGACGCG

ATGCACAGGCCTCAGATGATG

Dlx5a input file:

>dlx5a_Cyafur

GCAGACTTTGAGAGCTCCTTTCAACTCTCCAGCATGCACCATCCGTCTCAGGAATCTCCT

ACTTTACCCGAATCCTCCGCCACGGATTCTGGCTACTACAGTCCCGCTGGAGGAGTCGCT

CACGGCTACTGCTCGCCCAGCTCCACCTCTTACGGGAAGCCTCTGAATGCCTATCAGTAC

CAGTACCCGGGTGTAAACGGATCTGCTGGAAATTACTCGACAAAATCTTACACAGATTAC

AGCTCCTACACGACCCCCTCCTACCACCAGTATGCTGGGACTTACAGCAGAGTGCAAGCC

CAGCCGAGTCCACAAGAAAAAGAGATAAGCGAGCCTGAGGTGAGGATGGTGAATGGCAAG

CCGAAGAAAGTGAGAAAACCCCGGACAATCTACTCCAGCTTCCAGCTGGCCGCCTTGCAG

AGGCGGTTTCAGAACACACAGTACCTGGCACTGCCGGAGAGAGCCGAACTGGCCGCCTCG

CTGGGGCTAACGCAAACACAGGTGAAAATTTGGTTCCAAAACAGAAGATCAAAGATGAAG

AAGATCATGAAAAACGGCGAGCTTCCCCCGGAACACAGCCCCAGCTCCAGCGACCCCATG

GCCTGCAACTCTCCACAGTCCCCGGCCGTCTGGGACACACAGGGTCCCTCTAGGCCGCAC

AGTCTACAGCCACAAAGCATCAACACGACGGCTTCTAACTTTTTGGAAAACACGGGG-CA

TGGTACACCTCCGCCGGCAGCTCCATGACTTCCCACCTGCAGACCCCTAACTCAATTCAG

CATTCGTTGGCTCTTGGAGCGGGGACGTTATAT

>dlx5a_Neopul

GCAGACTTTGAGAGCTCCTTTCAACTCTCCAGCATGCACCATCCGTCTCAGGAATCTCCT

ACTTTACCCGAATCCTCCGCCACGGATTCTGGCTACTACAGTCCCGCTGGAGGAGTCGCT

CACGGCTACTGCTCGCCCAGCTCCACCTCTTACGGGAAGCCTCTGAATGCCTATCAGTAC

CAGTACCCGGGTGTAAACGGATCTGCTGGAAATTACTCGACAAAATCCTACACAGATTAC

AGCTCCTACACGACCCCCTCCTACCACCAGTATGCTGGGACTTACAGCAGAGTGCAAGCC

CAGCCGAGTCCACAAGAAAAAGAGATAAGCGAGCCCGAGGTGAGGATGGTGAATGGCAAG

CCGAAGAAAGTGAGAAAACCCCGGACAATCTACTCCAGCTTCCAGCTGGCCGCCTTGCAG

AGGCGGTTTCAGAACACACAGTACCTGGCGCTGCCGGAGAGAGCCGAACTGGCCGCCTCG

CTGGGGCTAACGCAAACACAGGTGAAAATTTGGTTCCAAAACAGAAGATCAAAGATGAAG

AAGATCATGAAAAACGGCGAGCTTCCCCCGGAACACAGCCCCAGCTCCAGCGACCCCATG

GCCTGCAACTCTCCACAGTCCCCGGCCGTCTGGGACACACAGGGTCCCTCCAGGCCGCAC

AGTCTACAGCCACAAAGCATCAACACGACGGCTTCTAACTTTTTGGAAAACACGGGGTCA

TGGTACACCTCAGCCGGCAGCTCCATGACATCCCACCTGCAGACCCCTAACTCAATACAG

CATTCGTTGGCTCTTGGAGCGGGGACGTTATAT

>dlx5a_Limsta

GCAGACTTTGAGAGCTCCTTTCAACTCTCCAGCATGCACCATCCGTCTCAGGAATCTCCT

ACTTTACCCGAATCCTCCGCCACGGATTCTGGCTACTACAGTCCCGCTGGAGGAGTCGCT

CACGGCTACTGCTCGCCCAGCTCCACCTCTTACGGGAAGCCTCTGAATGCCTATCAGTAC

CAGTACCCGGGTGTAAACGGATCTGCTGGAAATTACTCGACAAAATCCTACACAGATTAC

AGCTCCTACACGACCCCCTCCTACCACCAGTATGCTGGGACTTACAGCAGAGTGCAAGCC

CAGCCGAGTCCACAAGAAAAAGAGATAAGCGAGCCTGAGGTGAGGATGGTGAATGGCAAG

CCGAAGAAAGTGAGAAAACCCCGGACAATCTACTCCAGCTTCCAGCTGGCCGCCTTGCAG

AGGCGGTTTCAGAACACACAGTACCTGGCGCTGCCGGAGAGAGCCGAACTGGCCGCCTCG

CTGGGGCTAACGCAAACACAGGTGAAAATTTGGTTCCAAAACAGAAGATCAAAGATGAAG

AAGATCATGAAAAACGGCGAGCTTCCCCCGGAACACAGCCCCAGCTCCAGCGACCCCATG

GCCTGCAACTCTCCACAGTCCCCGGCCGTCTGGGACACACAGGGTCCCTCCAGGCCGCAC

AGTCTACAGCCACAAAGCATCAACACGACGGCTTCTAACTTTTTGGAAAACACGGGGTCA

TGGTACACCTCAGCCGGCAGCTCCATGACTTCCCACCTGCAGACCCCTAACTCAATACAG

CATTCGTTGGCTCTTGGAGCGGGGACGTTATAT

>dlx5a_Permic

GCAGACTTTGAGAGCTCCTTTCAACTCTCCAGCATGCACCATCCGTCTCAGGAATCTCCT

ACTTTACCCGAATCCTCCGCCACGGATTCTGGCTACTACAGTCCCGCTGGAGGAGTCGCT

CACGGCTACTGCTCGCCCAGCTCCACCTCTTACGGGAAGCCTCTGAATGCCTATCAGTAC

CAGTACCCGGGTGTAAACGGATCTGCTGGAAATTACTCGACAAAATCCTACACAGATTAC

AGCTCCTACACGACCCCCTCCTACCACCAGTATGCTGGGACTTACAGCAGAGTGCAAGCC

CAGCCGAGTCCACAAGAAAAAGAGATAAGCGAGCCTGAGGTGAGGATGGTGAATGGCAAG

CCGAAGAAAGTGAGAAAACCCCGGACAATCTACTCCAGCTTCCAGCTGGCCGCCTTGCAG

AGGCGGTTTCAGAACACACAGTACCTGGCGCTGCCGGAGAGAGCCGAACTGGCCGCCTCG

CTGGGGCTAACGCAAACACAGGTGAAAATTTGGTTCCAAAACAGAAGATCAAAGATGAAG

AAGATCATGAAAAACGGCGAGCTTCCCCCGGAACATAGCCCCAGCTCCAGCGACCCCATG

GCCTGCAACTCTCCACAGTCCCCGGCCGTCTGGGACACACAGGGTCCCTCCAGGCCGCAC

AGTCTACAGCCACAAAGCATCAACACGACGGCTTCTAACTTTTTGGAAAACACGGGGTCA

TGGTACACCTCAGCCGGCAGCTCCATGACTTCCCACCTGCAGACCCCTAACTCAATACAG

CATTCGTTGGCTCTTGGAGCGGGGACGTTATAT

>dlx5a_Plesta

GCAGACTTTGAGAGCTCCTTTCAACTCTCCAGCATGCACCATCCGTCTCAGGAATCTCCT

ACTTTACCCGAATCCTCCGCCACGGATTCTGGCTACTACAGTCCCGCTGGAGGAGTCGCT

CACGGCTACTGCTCGCCCAGCTCCACCTCTTACGGGAAGCCTCTGAATGCCTATCAGTAC

CAGTACCCGGGTGTAAACGGATCTGCTGGAAATTACTCGACAAAATCCTACACAGATTAC

AGCTCCTACACGACCCCCTCCTACCACCAGTATGCTGGGACTTACAGCAGAGTGCAAGCC

CAGCCGAGTCCACAAGAAAAAGAGATAAGCGAGCCTGAGGTGAGGATGGTGAATGGCAAG

CCGAAGAAAGTGAGAAAACCCCGGACAATCTACTCCAGCTTCCAGCTGGCCGCCTTGCAG

AGGCGGTTTCAGAACACACAGTACCTGGCGCTGCCGGAGAGAGCCGAACTGGCCGCCTCG

CTGGGGCTAACGCAAACACAGGTGAAAATTTGGTTCCAAAACAGAAGATCAAAGATGAAG

AAGATCATGAAAAACGGCGAGCTTCCCCCGGAACATAGCCCCAGCTCCAGCGACCCCATG

GCCTGCAACTCTCCACAGTCCCCGGCCGTCTGGGACACACAGGGTCCCTCCAGGCCGCAC

AGTCTACAGCCACAAAGCATCAACACGACGGCTTCTAACTTTTTGGAAAACACGGGGTCA

TGGTACACCTCAGCCGGCAGCTCCATGACTTCCCACCTGCAGACCCCTAACTCAATACAG

CATTCGTTGGCTCTTGGAGCGGGGACGTTATAT

>dlx5a_Tromoo

GCAGACTTTGAGAGCTCCTTTCAACTCTCCAGCATGCACCATCCGTCTCAGGAATCTCCT

ACTTTACCCGAATCCTCCGCCACGGATTCTGGCTACTACAGTCCCGCTGGAGGAGTCGCT

CACGGCTACTGCTCGCCCAGCTCCACCTCTTACGGGAAGCCTCTGAATGCCTATCAGTAC

CAGTACCCGGGTGTAAACGGATCTGCTGGAAATTACTCGACAAAATCCTACACAGATTAC

AGCTCCTACACGACCCCCTCCTACCACCAGTATGCTGGGACTTACAGCAGAGTGCAAGCC

CAGCCGAGTCCACAAGAAAAAGAGATAAGCGAGCCTGAGGTGAGGATGGTGAATGGCAAG

CCGAAGAAAGTGAGAAAACCCCGGACAATCTACTCCAGCTTCCAGCTGGCCGCCTTGCAG

AGGCGGTTTCAGAACACACAGTACCTGGCGCTGCCGGAGAGAGCCGAACTGGCCGCCTCG

CTGGGGCTAACGCAAACACAGGTGAAAATTTGGTTCCAAAACAGAAGATCAAAGATGAAG

AAGATCATGAAAAACGGCGAGCTTCCCCCGGAACACAGCCCCAGCTCCAGCGACCCCATG

GCCTGCAACTCTCCACAGTCCCCGGCCGTCTGGGACACACAGGGTCCCTCCAGGCCGCAC

AGTCTACAGCCACAAAGCATCAACACGACGGCTTCTAACTTTTTGGAAAACACGGGGTCA

TGGTACACCTCAGCCGGCAGCTCCATGACTTCCCACCTGCAGACCCCTAACTCAATACAG

CATTCGTTGGCTCTTGGAGCGGGGACGTTATAT

>dlx5a_Tylpol

GCAGACTTTGAGAGCTCCTTTCAACTCTCCAGCATGCACCATCCGTCTCAGGAATCTCCT

ACTTTACCCGAATCCTCCGCCACGGATTCTGGCTACTACAGTCCCGCTGGAGGAGTCGCT

CACGGCTACTGCTCGCCCAGCTCCACCTCTTACGGGAAGCCTCTGAATGCCTATCAGTAC

CAGTACCCGGGTGTAAACGGATCTGCTGGAAATTACTCGACAAAATCCTACACAGATTAC

AGCTCCTACACGACCCCCTCCTACCACCAGTATGCTGGGACTTACAGCAGAGTGCAAGCC

CAGCCGAGTCCACAAGAAAAAGAGATAAGCGAGCCTGAGGTGAGGATGGTGAATGGCAAG

CCGAAGAAAGTGAGAAAACCCCGGACAATCTACTCCAGCTTCCAGCTGGCCGCCTTGCAG

AGGCGGTTTCAGAACACACAGTACCTGGCGCTGCCGGAGAGAGCCGAACTGGCCGCCTCG

CTGGGGCTAACACAAACACAGGTGAAAATTTGGTTCCAAAACAGAAGATCAAAGATGAAG

AAGATCATGAAAAACGGCGAGCTTCCCCCGGAACACAGCCCCAGCTCCAGCGACCCCATG

GCCTGCAACTCTCCACAGTCCCCGGCCGTCTGGGACACACAGGGTCCCTCCAGGCCGCAC

AGTCTACAGCCACAAAGCATCAACACGACGGCTTCTAACTTTTTGGAAAACACGGGGTCA

TGGTACACCTCAGCCGGCAGCTCCATGACTTCCCACCTGCAGACCCCTAACTCAATACAG

CATTCGTTGGCTCTTGGAGCGGGGACGTTATAT

>dlx5a_Altfas

GCAGACTTTGAGAGCTCCTTTCAACTCTCCAGCATGCACCATCCGTCTCAGGAATCTCCT

ACTTTACCCGAATCCTCCGCCACGGATTCTGGCTACTACAGTCCCGCTGGAGGAGTCGCT

CACGGCTACTGCTCGCCCAGCTCCACCTCTTACGGGAAGCCTCTGAATGCCTATCAGTAC

CAGTACCCGGGTGTAAACGGATCTGCTGGAAATTACTCGACAAAATCCTACACAGATTAC

AGCTCCTACACGACCCCCTCCTACCACCAGTATGCTGGGACTTACAGCAGAGTGCAAGCC

CAGCCGAGTCCACAAGAAAAAGAGATAAGCGAGCCTGAGGTGAGGATGGTGAATGGCAAG

CCGAAGAAAGTGAGAAAACCCCGGACAATCTACTCCAGCTTCCAGCTGGCCGCCTTGCAG

AGGCGGTTTCAGAACACACAGTACCTGGCGCTGCCGGAGAGAGCCGAACTGGCCGCCTCG

CTGGGGCTAACGCAAACACAGGTGAAAATTTGGTTCCAAAACAGAAGATCAAAGATGAAG

AAGATCATGAAAAACGGCGAGCTTCCCCCGGAACACAGCCCCAGCTCCAGCGACCCCATG

GCCTGCAACTCTCCACAGTCCCCGGCCGTCTGGGACACACAGGGTCCCTCCAGGCCGCAC

AGTCTACAGCCACAAAGCATCAACACGACGGCTTCTAACTTTTTGGAAAACACGGGGTCA

TGGTACACCTCAGCCGGCAGCTCCATGACTTCCCACCTGCAGACCCCTAACTCAATACAG

CATTCGTTGGCTCTTGGAGCGGGGACGTTATAT

>dlx5a_Lepelo

GCAGACTTTGAGAGCTCCTTTCAACTCTCCAGCATGCACCATCCGTCTCAGGAATCTCCT

ACTTTACCCGAATCCTCCGCCACGGATTCTGGCTACTACAGTCCCGCTGGAGGAGTCGCT

CACGGCTACTGCTCGCCCAGCTCCACCTCTTACGGGAAGCCTCTGAATGCCTATCAGTAC

CAGTACCCGGGTGTAAACGGATCTGCTGGAAATTACTCGACAAAATCCTACACAGATTAC

AGCTCCTACACGACCCCCTCCTACCACCAGTATGCTGGGACTTACAGCAGAGTGCAAGCC

CAGCCGAGTCCACAAGAAAAAGAGATAAGCGAGCCTGAGGTGAGGATGGTGAATGGCAAG

CCGAAGAAAGTGAGAAAACCCCGGACAATCTACTCCAGCTTCCAGCTGGCCGCCTTGCAG

AGGCGGTTTCAGAACACACAGTACCTGGCGCTGCCGGAGAGAGCCGAACTGGCCGCCTCG

CTGGGGCTAACGCAAACACAGGTGAAAATTTGGTTCCAAAACAGAAGATCAAAGATGAAG

AAGATCATGAAAAACGGCGAGCTTCCCCCGGAACACAGCCCCAGCTCCAGCGACCCCATG

GCCTGCAACTCTCCACAGTCCCCGGCCGTCTGGGACACACAGGGTCCCTCCAGGCCGCAC

AGTCTACAGCCACAAAGCATCAACACGACGGCTTCTAACTTTTTGGAAAACACGGGGTCA

TGGTACACCTCAGCCGGCAGCTCCATGACTTCCCACCTGCAGACCCCTAACTCAATACAG

CATTCGTTGGCTCTTGGAGCGGGGACGTTATAT

>dlx5a_Varmoo

GCAGACTTTGAGAGCTCCTTTCAACTCTCCAGCATGCACCATCCGTCTCAGGAATCTCCT

ACTTTACCCGAATCCTCCGCCACGGATTCTGGCTACTACAGTCCCGCTGGAGGAGTCGCT

CACGGCTACTGCTCGCCCAGCTCCACCTCTTACGGGAAGCCTCTGAATGCCTATCAGTAC

CAGTACCCGGGTGTAAACGGATCTGCTGGAAATTACTCGACAAAATCCTACACAGATTAC

AGCTCCTACACGACCCCCTCCTACCACCAGTATGCTGGGACTTACAGCAGAGTGCAAGCC

CAGCCGAGTCCACAAGAAAAAGAGATAAGCGAGCCTGAGGTGAGGATGGTGAATGGCAAG

CCGAAGAAAGTGAGAAAACCCCGGACAATCTACTCCAGCTTCCAGCTGGCCGCCTTGCAG

AGGCGGTTTCAGAACACACAGTACCTGGCGCTGCCGGAGAGAGCCGAACTGGCTGCCTCG

CTGGGGCTAACGCAAACACAGGTGAAAATTTGGTTCCAAAACAGAAGATCAAAGATGAAG

AAGATCATGAAAAACGGCGAGCTTCCCCCGGAACACAGCCCCAGCTCCAGCGACCCCATG

GCCTGCAACTCTCCACAGTCCCCGGCCGTCTGGGACACACAGGGTCCCTCCAGGCCGCAC

AGTCTACAGCCACAAAGCATCAACACGACGGCTTCTAACTTTTTGGAAAACACGGGGTCA

TGGTACACCTCAGCCGGCAGCTCCATGACATCCCACCTGCAGACCCCTAACTCAATACAG

CATTCGTTGGCTCTTGGAGCGGGGACGTTATAT

>dlx5a_Batgra

GCAGACTTTGAGAGCTCCTTTCAACTCTCCAGCATGCACCATCCGTCTCAGGAATCTCCT

ACTTTACCCGAATCCTCCGCCACGGATTCTGGCTACTACAGTCCCGCTGGAGGAGTCGCT

CACGGCTACTGCTCGCCCAGCTCCACCTCTTACGGGAAGCCTCTGAATGCCTATCAGTAC

CAGTACCCGGGTGTAAACGGATCTGCTGGAAATTACTCGACAAAATCCTACACAGATTAC

AGCTCCTACACGACCCCCTCCTACCACCAGTATGCTGGGACTTACAGCAGAGTGCAAGCC

CAGCCGAGTCCACAAGAAAAAGAGATAAGCGAGCCTGAGGTGAGGATGGTGAATGGCAAG

CCGAAGAAAGTGAGAAAACCCCGGACAATCTACTCCAGCTTCCAGCTGGCCGCCTTGCAG

AGGCGGTTTCAGAACACACAGTACCTGGCGCTGCCGGAGAGAGCCGAACTGGCCGCCTCG

CTGGGGCTAACGCAAACACAGGTGAAAATTTGGTTCCAAAACAGAAGATCAAAGATGAAG

AAGATCATGAAAAACGGCGAGCTTCCCCCGGAACACAGCCCCAGCTCCAGCGACCCCATG

GCCTGCAACTCTCCACAGTCCCCGGCCGTCTGGGACACACAGGGTCCCTCCAGGCCGCAC

AGTCTACAACCACAAAGCATCAACACGACGGCTTCTAACTTTTTGGAAAACACGGGGTCA

TGGTACACCTCAGCCGGCAGCTCCATGACTTCCCACCTGCAGACCCCTAACTCAATACAG

CATTCGTTGGCTCTTGGAGCGGGGACGTTATAT

>dlx5a_Gnaper

GCAGACTTTGAGAGCTCCTTTCAACTCTCCAGCATGCACCATCCGTCTCAGGAATCTCCT

ACTTTACCCGAATCCTCCGCCACGGATTCTGGCTACTACAGTCCCGCTGGAGGAGTCGCT

CACGGCTACTGCTCGCCCAGCTCCACCTCTTACGGGAAGCCTCTGAATGCCTATCAGTAC

CAGTACCCGGGTGTAAACGGATCTGCTGGAAATTACTCGACAAAATCCTACACAGATTAC

AGCTCCTACACGACCCCCTCCTACCACCAGTATGCTGGGACTTACAGCAGAGTGCAAGCC

CAGCCGAGTCCACAAGAAAAAGAGATAAGCGAGCCTGAGGTGAGGATGGTGAATGGCAAG

CCGAAGAAAGTGAGAAAACCCCGGACAATCTACTCCAGCTTCCAGCTGGCCGCCTTGCAG

AGGCGGTTTCAGAACACACAGTACCTGGCGCTGCCGGAGAGAGCCGAACTGGCCGCCTCG

CTGGGGCTAACGCAAACACAGGTGAAAATTTGGTTCCAAAACAGAAGATCAAAGATGAAG

AAGATCATGAAAAACGGCGAGCTTCCCCCGGAACACAGCCCCAGCTCCAGCGACCCCATG

GCCTGCAACTCTCCACAGTCCCCGGCCGTCTGGGACACACAGGGTCCCTCCAGGCCGCAC

AGTCTACAGCCACAAAGCATCAACACGACGGCTTCTAACTTTTTGGAAAACACGGGGTCA

TGGTACACCTCAGCCGGCAGCTCCATGACTTCCCACCTGCAGACCCCTAACTCAATACAG

CATTCGTTGGCTCTTGGAGCGGGGACGTTATAT

>dlx5a_Oretan

GCAGACTTTGAGAGCTCCTTTCAACTCTCCAGCATGCACCATCCGTCTCAGGAATCTCCT

ACTTTACCCGAATCCTCCGCCACGGATTCTGGCTACTACAGTCCCGCTGGAGGAGTCGCT

CACGGCTATTGCTCGCCCAGCTCCACCTCTTACGGGAAGCCTCTGAATGCCTATCAGTAC

CAGTACCCGGGTGTAAACGGATCTGCTGGAAATTACTCGACAAAATCCTACACAGATTAC

AGCTCCTACACGACCCCCTCCTACCACCAGTATGCTGGGACTTACAGCAGAGTGCAAGCC

CAGCCGAGTCCACAAGAAAAAGAGATAAGCGAGCCTGAGGTGAGGATGGTGAATGGCAAG

CCGAAGAAAGTGAGAAAACCCCGGACAATCTACTCCAGCTTCCAGCTGGCCGCCCTGCAG

AGGCGGTTTCAGAACACACAGTACCTGGCGCTGCCGGAGAGAGCCGAGCTGGCCGCCTCG

CTGGGGCTAACGCAAACACAGGTGAAAATTTGGTTCCAAAACAGAAGATCAAAGATGAAG

AAGATCATGAAAAACGGCGAGCTTCCCCCGGAACACAGCCCCAGCTCCAGCGACCCCATG

GCCTGCAACTCTCCACAGTCCCCAGCCGTATGGGACACACAGGGTCCCTCCAGGCCGCAC

AGTCTACAGCCACAAAGCATCAACACGACGGCTTCTAACTTTTTGGAAAACACGGGGTCA

TGGTACACCTCAGCCGGCAGCTCCATGACTTCCCACCTGCAGACCCCTAACTCAATACAG

CATTCGTTGGCTCTTGGAGCGGGGACGTTATAT

>dlx5a_Petfam

GCAGACTTTGAGAGCTCCTTTCAACTCTCCAGCATGCACCATCCGTCTCAGGAATCTCCT

ACTTTACCCGAATCCTCCGCCACGGATTCTGGCTACTACAGTCCCGCTGGAGGAGTCGCT

CACGGCTACTGCTCGCCCAGCTCCACCTCTTACGGGAAGCCTCTGAATGCCTATCAGTAC

CAGTACCCGGGTGTAAACGGATCTGCTGGAAATTACTCGACAAAATCCTACACAGATTAC

AGCTCCTACACGACCCCCTCCTACCACCAGTATGCTGGGACTTACAGCAGAGTGCAAGCC

CAGCCGAGTCCACAAGAAAAAGAGATAAGCGAGCCTGAGGTGAGGATGGTGAATGGCAAG

CCGAAGAAAGTGAGAAAACCCCGGACAATCTACTCCAGCTTCCAGCTGGCCGCCTTGCAG

AGGCGGTTTCAGAACACACAGTACCTGGCGCTGCCGGAGAGAGCCGAACTGGCCGCCTCG

CTGGGGCTAACACAAACACAGGTGAAAATTTGGTTCCAAAACAGAAGATCAAAGATGAAG

AAGATCATGAAAAACGGCGAGCTTCCCCCGGAACACAGCCCCAGCTCCAGCGACCCCATG

GCCTGCAACTCTCCACAGTCCCCGGCCGTCTGGGACACACAGGGTCCCTCCAGGCCGCAC

AGTCTACAGCCACAAAGCATCAACACGACGGCTTCTAACTTTTTGGAAAACACGGGGTCA

TGGTACACCTCAGCCGGCAGCTCCATGACTTCCCACCTGCAGACCCCTAACTCAATACAG

CATTCGTTGGCTCTTGGAGCGGGGACGTTATAT

>dlx5a_Psecur

GCAGACTTTGAGAGCTCCTTTCAACTCTCCAGCATGCACCATCCGTCTCAGGAATCTCCT

ACTTTACCCGAATCCTCCGCCACGGATTCTGGCTACTACAGTCCCGCTGGAGGAGTCGCT

CACGGCTACTGCTCGCCCAGCTCCACCTCTTACGGGAAGCCTCTGAATGCCTATCAGTAC

CAGTACCCGGGTGTAAACGGATCTGCTGGAAATTACTCGACAAAATCCTACACAGATTAC

AGCTCCTACACGACCCCCTCCTACCACCAGTATGCTGGGACTTACAGCAGAGTGCAAGCC

CAGCCGAGTCCACAAGAAAAAGAGATAAGCGAGCCTGAGGTGAGGATGGTGAATGGCAAG

CCGAAGAAAGTGAGAAAACCCCGGACAATCTACTCCAGCTTCCAGCTGGCCGCCTTGCAG

AGGCGGTTTCAGAACACACAGTACCTGGCGCTGCCGGAGAGAGCCGAACTGGCCGCCTCG

CTGGGGCTAACACAAACACAGGTGAAAATTTGGTTCCAAAACAGAAGATCAAAGATGAAG

AAGATCATGAAAAACGGCGAGCTTCCCCCGGAACACAGCCCCAGCTCCAGCGACCCCATG

GCCTGCAACTCTCCACAGTCCCCGGCCGTCTGGGACACACAGGGTCCCTCCAGGCCGCAC

AGTCTACAGCCACAAAGCATCAACACGACGGCTTCTAACTTTTTGGAAAACACGGGGTCA

TGGTACACCTCAGCCGGCAGCTCCATGACTTCCCACCTGCAGACCCCTAACTCAATACAG

CATTCGTTGGCTCTTGGAGCGGGGACGTTATAT

>dlx5a_Boumic

GCAGACTTTGAGAGCTCCTTTCAACTCTCCAGCATGCACCATCCGTCTCAGGAATCTCCT

ACTTTACCCGAATCCTCCGCCACGGATTCTGGCTACTACAGTCCCGCTGGAGGAGTCGCT

CACGGCTACTGCTCGCCCAGCTCCACCTCTTACGGGAAGCCTCTGAATGCCTATCAGTAC

CAGTACCCGGGTGTAAACGGATCTGCTGGAAATTACTCGACAAAATCCTACACAGATTAC

AGCTCCTACACGACCCCCTCCTACCACCAGTATGCTGGGACTTACAGCAGAGTGCAAGCC

CAGCCGAGTCCACAAGAAAAAGAGATAAGCGAGCCTGAGGTGAGGATGGTGAATGGCAAG

CCGAAGAAAGTGAGAAAACCCCGGACAATCTACTCCAGCTTCCAGCTGGCCGCCTTGCAG

AGGCGGTTTCAGAACACACAGTACCTGGCGCTGCCGGAGAGAGCCGAACTGGCCGCCTCG

CTGGGGCTAACGCAAACACAGGTGAAAATTTGGTTCCAAAACAGAAGATCAAAGATGAAG

AAGATCATGAAAAACGGCGAGCTTCCCCCGGAACACAGCCCCAGCTCCAGCGACCCCATG

GCCTGCAATTCTCCACAGTCCCCGGCCGTCTGGGACACACAGGGTCCCTCCAGGCCGCAC

AGTCTACAGCCACAAAGCATCAACACGACGGCTTCTAACTTTTTGGAAAACACGGGGTCA

TGGTACACCTCAGCCGGCAGCTCCATGACTTCCCACCTGCAGACCCCTAACTCAATACAG

CATTCGTTGGCTCTTGGAGCGGGGACGTTATAT

>dlx5a_Cypfro

GCAGACTTTGAGAGCTCCTTTCAACTCTCCAGCATGCACCATCCGTCTCAGGAATCTCCT

ACTTTACCCGAATCCTCCGCCACGGATTCTGGCTACTACAGTCCCGCTGGAGGAGTCGCT

CACGGCTACTGCTCGCCCAGCTCCACCTCTTACGGGAAGCCTCTGAATGCCTATCAGTAC

CAGTACCCGGGTGTAAACGGATCTGCTGGAAATTACTCGACAAAATCCTACACAGATTAC

AGCTCCTACACGACCCCCTCCTACCACCAGTATGCTGGGACTTACAGCAGAGTGCAAGCC

CAGCCGAGTCCACAAGAAAAAGAGATAAGCGAGCCTGAGGTGAGGATGGTGAATGGCAAG

CCGAAGAAAGTGAGAAAACCCCGGACAATCTACTCCAGCTTCCAGCTGGCCGCCTTGCAG

AGGCGGTTTCAGAACACACAGTACCTGGCGCTGCCGGAGAGAGCCGAACTGGCCGCCTCG

CTGGGGCTAACGCAAACACAGGTGAAAATTTGGTTCCAAAACAGAAGATCAAAGATGAAG

AAGATCATGAAAAACGGCGAGCTTCCCCCGGAACACAGCCCCAGCTCCAGCGACCCCATG

GCCTGCAACTCTCCACAGTCCCCGGCCGTCTGGGACACACAGGGTCCCTCCAGGCCGCAC

AGTCTACAGCCACAAAGCATCAACACGACGGCTTCTAACTTTTTGGAAAACACGGGGTCA

TGGTACACCTCAGCCGGCAGCTCCATGACTTCCCACCTGCAGACCCCTAACTCAATACAG

CATTCGTTGGCTCTTGGAGCGGGGACGTTATAT

>dlx5a_Cyplep

GCAGACTTTGAGAGCTCCTTTCAACTCTCCAGCATGCACCATCCGTCTCAGGAATCTCCT

ACTTTACCCGAATCCTCCGCCACGGATTCTGGCTACTACAGTCCCGCTGGAGGAGTCGCT

CACGGCTACTGCTCGCCCAGCTCCACCTCTTACGGGAAGCCTCTGAATGCCTATCAGTAC

CAGTACCCGGGTGTAAACGGATCTGCTGGAAATTACTCGACAAAATCCTACACAGATTAC

AGCTCCTACACGACCCCCTCCTACCACCAGTATGCTGGGACTTACAGCAGAGTGCAAGCC

CAGCCGAGTCCACAAGAAAAAGAGATAAGCGAGCCTGAGGTGAGGATGGTGAATGGCAAG

CCGAAGAAAGTGAGAAAGCCCCGGACAATCTACTCCAGCTTCCAGCTGGCCGCCTTGCAG

AGGCGGTTTCAGAACACACAGTACCTGGCGCTGCCGGAGAGAGCCGAACTGGCCGCCTCG

CTGGGGCTAACGCAAACACAGGTGAAAATTTGGTTCCAAAACAGAAGATCAAAGATGAAG

AAGATCATGAAAAACGGCGAGCTTCCCCCGGAACATAGCCCCAGCTCCAGCGACCCCATG

GCCTGCAACTCTCCACAGTCCCCGGCCGTCTGGGACACACAGGGTCCCTCCAGGCCGCAC

AGTCTACAGCCACAAAGCATCAACACCACGGCTTCTAACTTTTTGGAAAACACGGGGTCA

TGGTACACCTCAGCCGGAAGCTCCATGACTTCCCACCTGCAGACCCCTAACTCAATACAG

CATTCGTTGGCTCTTGGAGCGGGGACGTTATAT

>dlx5a_Calmac

GCAGACTTTGAGAGCTCCTTTCAACTCTCCAGCATGCACCATCCGTCTCAGGAATCTCCT

ACTTTACCCGAATCCTCCGCCACGGATTCTGGCTACTACAGTCCCGCTGGAGGAGTCGCT

CACGGCTACTGCTCGCCCAGCTCCACCTCTTACGGGAAGCCTCTGAATGCCTATCAGTAC

CAGTACCCGGGTGTAAACGGATCTGCTGGAAATTACTCGACAAAATCTTACACAGATTAC

AGCTCCTACACGACCCCCTCCTACCACCAGTATGCTGGGACTTACAGCAGAGTGCAAGCC

CAGCCGAGTCCACAAGAAAAAGAGATAAGCGAGCCTGAGGTGAGGATGGTGAATGGCAAG

CCGAAGAAAGTGAGAAAACCCCGGACAATCTACTCCAGCTTCCAGCTGGCCGCCTTGCAG

AGGCGGTTTCAGAACACACAGTACCTGGCACTGCCGGAGAGAGCCGAACTGGCCGCCTCG

CTGGGGCTAACGCAAACACAGGTGAAAATTTGGTTCCAAAACAGAAGATCAAAGATGAAG

AAGATCATGAAAAACGGCGAGCTCCCCCCGGAACACAGCCCCAGCTCCAGCGACCCCATG

GCCTGCAACTCTCCACAGTCCCCGGCCGTCTGGGACACACAGGGTCCCTCTAGGCCGCAC

AGTCTACAGCCACAAAGCATCAACACGACGGCTTCTAACTTTTTGGAAAACACGGGGTCA

TGGTACACCTCAGCCGGCAGCTCCATGACTTCCCACCTGCAGACCCCTAACTCAATACAG

CATTCGTTGGCTCTTGGAGCGGGGACGTTATAT

>dlx5a_Astbur

GCAGACTTTGAGAGCTCCTTTCAACTCTCCAGCATGCACCATCCGTCTCAGGAATCTCCT

ACTTTACCCGAATCCTCCGCCACGGATTCTGGCTACTACAGTCCCGCTGGAGGAGTCGCT

CACGGCTACTGCTCGCCCAGCTCCACCTCTTACGGGAAGCCTCTGAATGCCTATCAGTAC

CAGTACCCGGGTGTAAATGGATCTGCTGGAAATTACTCGACAAAATCCTACACAGATTAC

AGCTCCTACACGACCCCCTCCTACCACCAGTATGCTGGGACTTACAGCAGAGTGCAAGCC

CAGCCGAGTCCACAAGAAAAAGAGATAAGCGAGCCTGAGGTGAGGATGGTGAATGGCAAG

CCGAAGAAAGTGAGAAAACCCCGGACAATCTACTCCAGCTTCCAGCTGGCCGCCTTGCAG

AGGCGGTTTCAGAACACACAGTACCTGGCGCTGCCGGAGAGAGCCGAACTGGCCGCCTCG

CTGGGGCTAACGCAAACACAGGTGAAAATTTGGTTCCAAAACAGAAGATCAAAGATGAAG

AAGATCATGAAAAACGGCGAGCTTCCCCCGGAACACAGCCCCAGCTCCAGCGACCCCATG

GCCTGCAACTCTCCACAGTCCCCGGCCGTCTGGGACACACAGGGTCCCTCCAGGCCGCAC

AGTCTACAGCCACAAAGCATCAACACGACGGCTTCTAACTTTTTGGAAAACACGGGGTCA

TGGTACACCTCAGCCGGCAGCTCCATGACTTCCCACCTGCAGACCCCTAACTCAATACAG

CATTCGTTGGCTCTTGGAGCGGGGACGTTATAT

>dlx5a_Ctehor

GCAGACTTTGAGAGCTCCTTTCAACTCTCCAGCATGCACCATCCGTCTCAGGAATCTCCT

ACTTTACCCGAATCCTCCGCCACGGATTCTGGCTACTACAGTCCCGCTGGAGGAGTCGCT

CACGGCTACTGCTCGCCCAGCTCCACCTCTTACGGGAAGCCTCTGAATGCCTATCAGTAC

CAGTACCCGGGTGTAAACGGATCTGCTGGAAATTACTCGACAAAATCCTACACAGATTAC

AGCTCCTACACGACCCCCTCCTACCACCAGTATGCTGGGACTTACAGCAGAGTGCAAGCC

CAGCCGAGTCCACAAGAAAAAGAGATAAGCGAGCCTGAGGTGAGGATGGTGAATGGCAAG

CCGAAGAAAGTGAGAAAACCCCGGACAATCTACTCCAGCTTCCAGCTGGCCGCCTTGCAG

AGGCGGTTTCAGAACACACAGTACCTGGCGCTGCCGGAGAGAGCCGAACTGGCCGCCTCG

CTGGGGCTAACACAAACACAGGTGAAAATTTGGTTCCAAAACAGAAGATCAAAGATGAAG

AAGATCATGAAAAACGGCGAGCTTCCCCCGGAACACAGCCCCAGCTCCAGCGACCCCATG

GCCTGCAACTCTCCACAGTCCCCGGCCGTCTGGGACACACAGGGTCCCTCCAGGCCGCAC

AGTCTACAGCCACAAAGCATCAACACGACGGCTTCTAACTTTTTGGAAAACACGGGGTCA

TGGTACACCTCAGCCGGCAGCTCCATGACTTCCCACCTGCAGACCCCTAACTCAATACAG

CATTCGTTGGCTCTTGGAGCGGGGACGTTATAT

>dlx5a_Neofur

GCAGACTTTGAGAGCTCCTTTCAACTCTCCAGCATGCACCATCCGTCTCAGGAATCTCCT

ACTTTACCCGAATCCTCCGCCACGGATTCTGGCTACTACAGCCCCGCTGGAGGAGTCGCT

CACGGCTACTGCTCGCCCAGCTCCACCTCTTACGGGAAGCCTCTGAATGCCTATCAGTAC

CAGTACCCGGGTGTAAACGGATCTGCTGGAAATTACTCGACAAAATCCTACACAGATTAC

AGCTCCTACACGACCCCCTCCTACCACCAGTATGCTGGGACTTACAGCAGAGTGCAAGCC

CAGCCGAGTCCGCAAGAAAAAGAGATAAGCGAGCCTGAGGTGAGGATGGTGAATGGCAAG

CCGAAGAAAGTGAGAAAACCCCGGACAATCTACTCCAGCTTCCAGCTGGCCGCCTTGCAG

AGGCGGTTTCAGAACACACAGTACCTGGCGCTGCCGGAGAGAGCCGAACTGGCCGCCTCG

CTGGGGCTAACGCAAACACAGGTGAAAATTTGGTTCCAAAACAGAAGATCAAAGATGAAG

AAGATCATGAAAAACGGCGAGCTTCCCCCGGAACACAGCCCCAGCTCCAGCGACCCCATG

GCCTGCAACTCTCCACAGTCCCCGGCCGTCTGGGACACACAGGGTCCCTCCAGGCCGCAC

AGTCTACAGCCACAAAGCATCAACACGACGGCTTCTAACTTTTTGGAAAACACGGGGTCA

TGGTACACCTCAGCCGGCAGCTCCATGACATCCCACCTGCAGACCCCTAACTCAATACAG

CATTCGTTGGCTCTTGGAGCGGGGACGTTATAT

>dlx5a_Loblab

GCAGACTTTGAGAGCTCCTTTCAACTCTCCAGCATGCACCATCCGTCTCAGGAATCTCCT

ACTTTACCCGAATCCTCCGCCACGGATTCTGGCTACTACAGTCCCGCTGGAGGAGTCGCT

CACGGCTACTGCTCGCCCAGCTCCACCTCTTACGGGAAGCCTCTGAATGCCTATCAGTAC

CAGTACCCGGGTGTAAACGGATCTGCTGGAAATTACTCGACAAAATCCTACACAGATTAC

AGCTCCTACACGACCCCCTCCTACCACCAATATGCTGGGACTTACAGCAGAGTGCAAGCC

CAGCCGAGTCCACAAGAAAAAGAGATAAGCGAGCCTGAGGTGAGGATGGTGAATGGCAAG

CCGAAGAAAGTGAGAAAACCCCGGACAATCTACTCCAGCTTCCAGCTGGCCGCCTTGCAG

AGGCGGTTTCAGAACACACAGTACCTGGC-------------------------------

------------------------------------------------------------

------------------------------------------------------------

------------------------------------------------------------

------------------------------------------------------------

------------------------------------------------------------

---------------------------------

Dlx6a input file:

>dlx6a_Batgra

CTGGAGGCTCAGGACTCGTCCAAGTCTGCTTTCATGGAGTTTGGACAGCAGTCGCACTCA

CAGCAGAGCTCCCCATCGATGGGCAGCGGCCACTACCCGCTGCACTGTCTCCACTCCGGC

TCACACTCTCACCACCAGCACGACAACACCCCGTACCCCGGGAGCAACACCTACAACAGG

TCGTTACCTTACCCTTACGTGAGCCATCCACACCACAGCCCGTACCTGCCGTCCTATCAC

AACAACATGGGAGGACAGACAAGGTTAGACGGCGCAGAGCAGCAGAAGACGACAGTGATC

GAAAACGGGGAGATTCGTTTTAACGGCAAAGGCAAGAAGATTCGCAAACCTCGGACAATT

TATTCCAGTTTGCAGCTTCAAGCACTGAACCACCGTTTCCAGCAAACACAGTACCTCGCT

TTACCGGAGCGCGCCGAGCTGGCCGCCTCTCTAGGACTGACGCAAACACAGgTAAAAATT

TGGTTTCAGAATAAAAGGTCAAAGTTCAAGAAGCTGCTGAAGCAAGGCAGTAACCCGCAC

GAGAGCGAGCCCATCCCGGGCTCCATGTCCCTGTCCCCACGCTCGCCGAGCATCCCCCCA

ATCTGGGATGTGTCGGCCTCATCCAAAGGAGTAAACATGCCGGCCAACAGCTACATGCCC

GGCTACTCTCACTGGTATTCCTCCCCACATCAAGACTCAATGCAGAGA

>dlx6a_Boumic

CTGGAGGCTCAGGACTCGTCCAAGTCTGCTTTCATGGAGTTTGGACAGCAGTCGCACTCA

CAGCAGAGCTCCCCATCGATGGGCAGCGGCCACTACCCGCTGCACTGTCTCCACTCCGGC

TCACACTCTCACCACCAGCACGACAACACCCCGTACCCCGGGAGCAACACCTACAACAGG

TCGTTACCTTACCCTTACGTGAGCCATCCACACCACAGCCCGTACCTGCCGTCCTATCAC

AACAACATGGGAGGACAGACAAGGTTAGACGGCACAGAGCAGCAGAAGACGACAGTGATC

GAAAACGGGGAGATTCGTTTTAACGGCAAAGGCAAGAAGATTCGCAAACCTCGGACAATT

TATTCCAGTTTGCAGCTTCAAGCACTGAACCACCGTTTCCAGCAAACACAGTACCTCGCT

TTACCGGAGCGCGCCGAGCTGGCCGCCTCTCTAGGACTGACGCAAACACAGgTAAAAATT

TGGTTTCAGAATAAAAGGTCAAAGTTCAAGAAGCTGCTGAAGCAAGGCGGTAACCCGCAC

GAGAGCGAGCCCATCCCGGGCTCCATGTCCCTGTCCCCGCGCTCGCCGAGCATCCCCCCA

ATCTGGGACGTGTCGGCCTCATCCAAAGGAGTAAACATGCCGGCCAACAGCTACATGCCC

GGCTACTCTCACTGGTATTCCTCCCCACATCAAGACTCAATGCAGAGA

>dlx6a_Cypfro

CTGGAGGCTCAGGACTCGTCCAAGTCTGCTTTCATGGAGTTTGGACAGCAGTCGCACTCA

CAGCAGAGCTCCCCATCGATGGGCAGCGGCCACTACCCGCTGCACTGTCTCCACTCCGGC

TCACACTCTCACCACCAGCACGACAACACCCCGTACCCCGGGAGCAACACCTACAACAGG

TCGTTACCTTACCCTTACGTGAGCCATCCACACCACAGCCCGTACCTGCCGTCCTATCAC

AACAACATGGGAGGACAGACAAGGTTAGACGGCACAGAGCAGCAGAAGACGACAGTGATC

GAAAACGGGGAGATTCGTTTTAACGGCAAAGGCAAGAAGATTCGCAAACCTCGGACAATT

TATTCCAGTTTGCAGCTTCAAGCACTGAACCACCGTTTCCAGCAAACACAGTACCTCGCT

TTACCGGAGCGCGCCGAGCTGGCCGCCTCTCTAGGACTGACGCAAACACAGgTAAAAATT

TGGTTTCAGAATAAAAGGTCAAAGTTCAAGAAGCTGCTGAAGCAAGGCGGTAACCCGCAC

GAGAGCGAGCCCATCCCGGGCTCCATGTCCCTGTCCCCGCGCTCTCCGAGCATCCCCCCA

ATCTGGGACGTGTCGGCCTCATCCAAAGGAGTAAACATGCCGGCCAACAGCTACATGCCC

GGCTACTCTCACTGGTATTCCTCCCCACATCAAGACTCAATGCAGAGA

>dlx6a_Cyplep

------------------------------------------------------------

------------------------------------------------------------

------------------------------------------------------------

------------------------------------------------------------

-------------------------------------AGCAGCAGAAGACGACAGTGATC

GAAAACGGGGAGATTCGTTTTAACGGCAAAGGCAAGAAGATTCGCAAACCTCGGACAATT

TATTCCAGTTTGCAGCTTCAAGCACTGAACCACCGTTTCCAGCAAACACAGTACCTCGCT

TTACCGGAGCGCGCCGAGCTGGCCGCCTCTCTAGGACTGACGCAAACACAGgTAAAAATT

TGGTTTCAGAATAAAAGGTCAAAGTTCAAGAAGCTGCTGAAGCAAGGCGGTAACCCGCAC

GAGAGCGAACCCATCCCGGGCTCCATGTCGCTGTCCCCGCGCTCTCCGAGCATCCCCCCA

ATCTGGGACGTGTCGGCCTCATCCAAAGGAGTAAACATGCCGGCCAACAGCTACATGCCC

GGCTACTCTCACTGGTATTCCTCCCCACATCAAGACTCAATGCAGAGA

>dlx6a_Cyafur

CTGGAGGCTCAGGACTCGTCCAAGTCTGCTTTCATGGAGTTTGGACAGCAGTCGCACTCA

CAGCAGAGCTCCCCATCGATGGGCAGCGGCCACTACCCGCTGCACTGTCTCCACTCCGGC

TCACACTCTCACCACCAGCACGACAACACCCCGTACCCCGGGAGCAACACCTACAATAGG

TCGTTACCTTACCCTTACGTGAGCCATCCACACCACAGCCCGTACCTGCCGTCCTATCAC

AACAACATGGGAGGACAGACAAGGTTAGACGGCACAGAGCAGCAGAAGACGACAGTGATC

GAAAACGGGGAGATTCGTTTTAACGGCAAAGGCAAGAAGATTCGCAAACCTCGGACAATT

TATTCCAGTTTGCAGCTTCAAGCACTGAACCACCGTTTCCAGCAAACACAGTACCTCGCT

TTACCGGAGCGCGCCGAGCTGGCCGCCTCTCTAGGACTGACGCAAACACAGgTAAAAATT

TGGTTTCAGAATAAAAGGTCAAAGTTCAAGAAGCTGCTGAAGCAAGGCGGTAACCCGCAC

GAGAGCGAGCCCATCCCGGGCTCCATGTCCCTGTCCCCGCGCTCGCCGAGCATCCCCCCA

ATCTGGGACGTGTCGGCCTCATCCAAAGGAGTAAACATGCCGGCCAACAGCTACATGCCC

GGCTACTCTCACTGGTATTCCTCCCCACATCAAGACTCAATGCAGAGA

>dlx6a_Astbur

CTGGAGGCTCAGGACTCGTCCAAGTCTGCTTTCATGGAGTTTGGACAGCAGTCGCACTCA

CAGCAGAGCTCCCCATCGATGGGCAGCGGCCACTACCCGCTGCACTGTCTCCACTCCGGC

TCACACTCTCACCACCAGCACGACAACACCCCGTACCCCGGGAGCAACACCTACAACAGG

TCGTTACCTTACCCTTACGTGAGCCATCCACACCACAGCCCGTACCTGCCGTCCTATCAC

AACAACATGGGAGGACAGACAAGGTTAGACGGCACAGAGCAGCAGAAGACGACAGTGATC

GAAAACGGGGAGATTCGTTTTAACGGCAAAGGCAAGAAGATTCGCAAACCTCGGACAATT

TATTCCAGTTTGCAGCTTCAAGCACTGAACCACCGTTTCCAGCAAACACAGTACCTCGCT

TTACCGGAGCGCGCCGAGCTGGCCGCCTCTCTAGGACTGACGCAAACACAGgTAAAAATT

TGGTTTCAGAATAAAAGGTCAAAGTTCAAGAAGCTGCTGAAGCAAGGCGGTAACCCGCAC

GAGAGCGAGCCCATCCCGGGCTCCATGTCCCTGTCCCCGCGCTCTCCGAGCATCCCCCCA

ATCTGGGACGTGTCGGCCTCATCCAAAGGAGTAAACATGCCGGCCAACAGCTACATGCCC

GGCTACTCTCACTGGTATTCCTCCCCACATCAAGACTCAATGCAGAGA

>dlx6a_Ctehor

CTGGAGGCTCAGGACTCGTCCAAGTCTGCTTTCATGGAGTTTGGACAGCAGTCGCACTCA

CAGCAGAGCTCCCCATCGATGGGCAGCGGCCACTACCCGCTGCACTGTCTCCACTCCGGC

TCACACTCTCACCACCAGCACGACAACACTCCGTACCCCGGGAGCAACACCTACAACAGG

TCGTTACCTTACCCTTACGTGAGCCATCCACACCACAGCCCGTACCTGCCGTCCTATCAC

AACAACATGGGAGGACAGACAAGGTTAGACGGCACAGAGCAGCAGAAGACGACAGTGATC

GAAAACGGGGAGATTCGTTTTAACGGCAAAGGCAAGAAGATTCGCAAACCTCGGACAATT

TATTCCAGTTTGCAGCTTCAAGCACTGAACCACCGTTTCCAGCAAACACAGTACCTCGCT

TTACCGGAGCGCGCCGAGCTGGCCGCCTCTCTAGGACTGACGCAAACACAGgTAAAAATT

TGGTTTCAGAATAAAAGGTCAAAGTTCAAGAAGCTGCTGAAGCAAGGCGGTAACCCGCAC

GAGAGCGAGCCTATCCCGGGCTCCATGTCCCTGTCCCCGCGCTCGCCGAGCATCCCCCCA

ATCTGGGACGTGTCGGCCTCATCCAAAGGAGTAAACATGCCGGCCAACAGCTACATGCCT

GGCTACTCTCACTGGTATTCCTCCCCACATCAAGACTCAATGCAGAGA

>dlx6a_Neofur

CTGGAGGCTCAGGACTCGTCCAAGTCTGCTTTCATGGAGTTTGGACAGCAGTCGCACTCA

CAGCAGAGCTCCCCATCGATGGGCAGCGGCCACTACCCGCTGCACTGTCTCCACTCCGGC

TCACACTCTCACCACCAGCACGACAACACCCCGTACCCCGGGAGCAACACCTACAACAGG

TCGTTACCTTACCCTTACGTGAGCCATCCACACCACAGCCCGTACCTGCCGTCCTATCAC

AACAACATGGGAGGACAGACAAGGTTAGACGGCACAGAGCAGCAGAAGACGACAGTGATC

GAAAACGGGGAGATTCGTTTTAACGGCAAAGGCAAGAAGATTCGCAAACCTCGGACAATT

TATTCCAGTTTGCAGCTTCAAGCACTGAACCACCGTTTCCAGCAAACACAGTACCTCGCT

TTACCGGAGCGCGCCGAGCTGGCCGCCTCTCTAGGACTGACGCAAACACAGgTAAAAATT

TGGTTTCAGAATAAAAGGTCAAAGTTCAAGAAGCTGCTGAAGCAAGGCGGTAACCCGCAC

GAGAGCGAGCCCATCCCGGGCTCCATGTCCCTGTCCCCGCGCTCGCCGAGCATCCCCCCA

ATCTGGGACGTGTCGGCCTCATCCAAAGGAGTAAACATGCCGGCCAACAGCTACATGCCC

GGCTACTCTCACTGGTATTCCTCCCCACATCAAGACTCAATGCAGAGA

>dlx6a_Neopul

CTGGAGGCTCAGGACTCGTCCAAGTCTGCTTTCATGGAGTTTGGACAGCAGTCGCACTCA

CAGCAGAGCTCCCCATCGATGGGCAGCGGCCACTACCCGCTGCACTGTCTCCACTCCGGC

TCACACTCTCACCACCAGCACGACAACACCCCGTACCCCGGGAGCAACACCTACAACAGG

TCGTTACCTTACCCTTACGTGAGCCATCCACACCACAGCCCGTACCTGCCGTCCTATCAC

AACAACATGGGAGGACAGACAAGGTTAGACGGCACAGAGCAGCAGAAGACGACAGTGATC

GAAAACGGGGAGATTCGTTTTAACGGCAAAGGCAAGAAGATTCGCAAACCTCGGACAATT

TATTCCAGTTTGCAGCTTCAAGCACTGAACCACCGTTTCCAGCAAACACAGTACCTCGCT

TTACCGGAGCGCGCCGAGCTGGCCGCCTCTCTAGGACTGACGCAAACACAGgTAAAAATT

TGGTTTCAGAATAAAAGGTCAAAGTTCAAGAAGCTGCTGAAGCAAGGCGGTAACCCGCAC

GAGAGCGAGCCCATCCCGGGCTCCATGTCCCTGTCCCCGCGCTCGCCGAGCATCCCCCCA

ATCTGGGACGTGTCGGCCTCATCCAAAGGAGTAAACATGCCGGCCAACAGCTACATGCCC

GGCTACTCTCACTGGTATTCCTCCCCACATCAAGACTCAATGCAGAGA

>dlx6a_Limsta

CTGGAGGCTCAGGACTCGTCCAAGTCTGCTTTCATGGAGTTTGGACAGCAGTCGCACTCA

CAGCAGAGCTCCCCATCGATGGGCAGCGGCCACTACCCGCTGCACTGTCTCCACTCCGGC

TCACACTCTCACCACCAGCACGACAACACCCCGTACCCCGGGAGCAACACCTACAACAGG

TCGTTACCTTACCCTTACGTGAGCCATCCACACCACAGCCCGTACCTGCCGTCCTATCAC

AACAACATGGGAGGACAGACAAGGTTAGACGGCACAGAGCAGCAGAAGACGACAGTGATC

GAAAACGGGGAGATTCGTTTTAACGGCAAAGGCAAGAAGATTCGCAAACCTCGGACAATT

TATTCCAGTTTGCAGCTTCAAGCACTGAACCACCGTTTCCAGCAAACACAGTACCTCGCT

TTACCGGAGCGCGCCGAGCTGGCCGCCTCTCTAGGACTGACGCAAACACAGgTAAAAATT

TGGTTTCAGAATAAAAGGTCAAAGTTCAAGAAGCTGCTGAAGCAAGGCGGTAACCCGCAC

GAGAGCGAGCCCATCCCGGGCTCCATGTCCCTGTCCCCGCGCTCGCCGAGCATCCCCCCA

ATCTGGGACGTGTCGGCCTCATCCAAAGGAGTAAACATGCCGGCCAACAGCTACATGCCC

GGCTACTCTCACTGGTATTCCTCCCCACATCAAGACTCAATGCAGAGA

>dlx6a_Permic

CTGGAGGCTCAGGACTCGTCCAAGTCTGCTTTCATGGAGTTTGGACAGCAGTCGCACTCA

CAGCAGAGCTCCCCATCGATGGGCAGCGGCCACTACCCGCTGCACTGTCTCCACTCCGGC

TCACACTCTCACCACCAGCACGACAACACCCCGTACCCCGGGAGCAACACCTACAACAGG

TCGTTACCTTACCCTTACGTGAGCCATCCACACCACAGCCCGTACCTGCCGTCCTATCAC

AACAACATGGGAGGACAGACAAGGTTAGACGGCACAGAGCAGCAGAAGACGACAGTGATC

GAAAACGGGGAGATTCGTTTTAACGGCAAAGGCAAGAAGATTCGCAAACCTCGGACAATT

TATTCCAGTTTGCAGCTTCAAGCACTGAACCACCGTTTCCAGCAAACACAGTACCTCGCT

TTACCGGAGCGCGCCGAGCTGGCCGCCTCTCTAGGACTGACGCAAACACAGgTAAAAATT

TGGTTTCAGAATAAAAGGTCAAAGTTCAAGAAGCTGCTGAAGCAAGGCGGTAACCCGCAC

GAGAGCGAGCCCATCCCGGGCTCCATGTCCCTGTCCCCGCGCTCTCCGAGCATCCCCCCA

ATCTGGGACGTGTCGGCCTCATCCAAAGGAGTAAACATGCCGGCCAACAGCTACATGCCC

GGCTACTCTCACTGGTATTCCTCCCCACATCAAGACTCAATGCAGAGA

>dlx6a_Loblab

CTGGAGGCTCAGGACTCGTCCAAGTCTGCTTTCATGGAGTTTGGACAGCAGTCGCACTCA

CAGCAGAGCTCCCCATCGATGGGCAGCGGCCACTACCCGCTGCACTGTCTCCACTCCGGC

TCACACTCTCACCACCAGCACGACAACACCCCGTACCCCGGGAGCAACACCTACAACAGG

TCGTTACCTTACCCTTACGTGAGCCATCCACACCACAGCCCGTACCTGCCGTCCTATCAC

AACAACATGGGAGGACAGACAAGGTTAGACGGCACAGAGCAGCAGAAGACGACAGTGATC

GAAAACGGGGAGATTCGTTTTAACGGCAAAGGCAAGAAGATTCGCAAACCTCGGACAATT

TATTCCAGTTTGCAGCTTCAAGCACTGAACCACCGTTTCCAGCAAACACAGTACCTCGCT

TTACCGGAGCGCGCCGAGCTGGCCGCCTCTCTAGGACTGACGCAAACACAGgTAAAAATT

TGGTTTCAGAATAAAAGGTCAAAGTTCAAGAAGCTGCTGAAGCAAGGCGGTAACCCGCAC

GAGAGCGAGCCCATCCCGGGCTCCATGTCCCTGTCCCCGCGCTCGCCGAGCATCCCCCCA

ATCTGGGACGTGTCGGCCTCATCCAAAGGAGTAAACATGCCGGCCAACAGCTACATGCCT

GGCTACTCTCACTGGTATTCCTCCCCACATCAAGACTCAATGCAGAGA

>dlx6a_Tromoo

CTGGAGGCTCAGGACTCGTCCAAGTCTGCTTTCATGGAGTTTGGACAGCAGTCGCACTCA

CAGCAGAGCTCCCCATCGATGGGCAGCGGCCACTACCCGCTGCACTGTCTCCACTCCGGC

TCACACTCTCACCACCAGCACGACAACACCCCGTACCCCGGGAGCAACACCTACAACAGG

TCGTTACCTTACCCTTACGTGAGCCATCCACACCACAGCCCGTACCTGCCGTCCTATCAC

AACAACATGGGAGGACAGACAAGGTTAGACGGCACAGAGCAGCAGAAGACGACAGTGATC

GAAAACGGGGAGATTCGTTTTAACGGCAAAGGCAAGAAGATTCGCAAACCTCGGACAATT

TATTCCAGTTTGCAGCTTCAAGCACTGAACCACCGTTTCCAGCAAACACAGTACCTCGCT

TTACCGGAGCGCGCCGAGCTGGCCGCCTCTCTAGGACTGACGCAAACCCAGgTAAAAATT

TGGTTTCAGAATAAAAGGTCAAAGTTCAAGAAGCTGCTGAAGCAAGGCGGTAACCCGCAC

GAGAGCGAGCCCATCCCGGGCTCCATGTCCCTGTCCCCGCGCTCGCCGAGCATCCCCCCA

ATCTGGGACGTGTCGGCCTCATCCAAAGGAGTAAACATGCCGGCCAACAGCTACATGCCT

GGCTACTCTCACTGGTATTCCTCCCCACATCAAGACTCAATGCAGAGA

>dlx6a_Tylpol

CTGGAGGCTCAGGACTCGTCCAAGTCTGCTTTCATGGAGTTTGGACAGCAGTCGCACTCA

CAGCAGAGCTCCCCATCGATGGGCAGCGGCCACTACCCGCTGCACTGTCTCCACTCCGGC

TCACACTCTCACCACCAGCACGACAACACTCCGTACCCCGGGAGCAACACCTACAACAGG

TCGTTACCTTACCCTTACGTGAGCCATCCACACCACAGCCCGTACCTGCCGTCCTATCAC

AACAACATGGGAGGACAGACAAGGTTAGACGGCACAGAGCAGCAGAAGACGACAGTGATC

GAAAACGGGGAGATTCGTTTTAACGGCAAAGGCAAGAAGATTCGCAAACCTCGGACAATT

TATTCCAGTTTGCAGCTTCAAGCACTGAACCACCGTTTCCAGCAAACACAGTACCTCGCT

TTACCGGAGCGCGCCGAGCTGGCCGCCTCTCTAGGACTGACGCAAACACAGgTAAAAATT

TGGTTTCAGAATAAAAGGTCAAAGTTCAAGAAGCTGCTGAAGCAAGGCGGTAACCCGCAC

GAGAGCGAGCCCATCCCGGGCTCCATGTCCCTGTCCCCGCGCTCGCCGAGCATCCCCCCA

ATCTGGGACGTGTCGGCCTCATCCAAAGGAGTAAACATGCCGGCCAACAGCTACATGCCT

GGCTACTCTCACTGGTATTCCTCCCCACATCAAGACTCAATGCAGAGA

>dlx6a_Altfas

CTGGAGGCTCAGGACTCGTCCAAGTCTGCTTTCATGGAGTTTGGACAGCAGTCGCACTCA

CAGCAGAGCTCCCCATCGATGGGCAGCGGCCACTACCCGCTGCACTGTCTCCACTCCGGC

TCACACTCTCACCACCAGCACGACAACACCCCGTACCCCGGGAGCAACACCTACAACAGG

TCGTTACCTTACCCTTACGTGAGCCATCCACACCACAGCCCGTACCTGCCGTCCTATCAC

AACAACATGGGAGGACAGACAAGGTTAGACGGCACAGAGCAGCAGAAGACGACAGTGATC

GAAAACGGGGAGATTCGTTTTAACGGCAAAGGCAAGAAGATTCGCAAACCTCGGACAATT

TATTCCAGTTTGCAGCTTCAAGCACTGAACCACCGTTTCCAGCAAACACAGTACCTCGCT

TTACCGGAGCGCGCCGAGCTGGCCGCCTCTCTAGGACTGACGCAAACACAGgTAAAAATT

TGGTTTCAGAATAAAAGGTCAAAGTTCAAGAAGCTGCTGAAGCAAGGCGGTAACCCGCAC

GAGAGCGAGCCCATCCCGGGCTCCATGTCCCTGTCCCCGCGCTCGCCGAGCATCCCCCCA

ATCTGGGACGTGTCGGCCTCATCCAAAGGAGTAAACATGCCGGCCAACAGCTACATGCCC

GGCTACTCTCACTGGTATTCCTCCCCACATCAAGACTCAATGCAGAGA

>dlx6a_Lepelo

CTGGAGGCTCAGGACTCGTCCAAGTCTGCTTTCATGGAGTTTGGACAGCAGTCGCACTCA

CAGCAGAGCTCCCCATCGATGGGCAGCGGCCACTACCCGCTGCACTGTCTTCACTCCGGC

TCACACTCTCACCACCAGCACGACAACACCCCGTACCCCGGGAGCAACACCTACAACAGG

TCGTTACCTTACCCTTACGTGAGCCATCCACACCACAGCCCGTACCTGCCGTCCTATCAC

AACAACATGGGAGGACAGACAAGGTTAGACGGCACAGAGCAGCAGAAGACGACAGTGATC

GAAAACGGGGAGATTCGTTTTAACGGCAAAGGCAAGAAGATTCGCAAACCTCGGACAATT

TATTCCAGTTTGCAGCTTCAAGCACTGAACCACCGTTTCCAGCAAACACAGTACCTCGCT

TTACCGGAGCGCGCCGAGCTGGCCGCCTCTCTAGGACTGACGCAAACACAGgTAAAAATT

TGGTTTCAGAATAAAAGGTCAAAGTTCAAGAAGCTGCTGAAGCAAGGCGGTAACCCGCAC

GAGAGCGAGCCCATCCCGGGCTCCATGTCCCTGTCCCCGCGCTCGCCGAGCATCCCCCCA

ATCTGGGACGTGTCGGCCTCATCCAAAGGAGTAAACATGCCGGCCAACAGCTACATGCCC

GGCTACTCTCACTGGTATTCCTCCCCACATCAAGACTCAATGCAGAGA

>dlx6a_Varmoo

CTGGAGGCTCAGGACTCGTCCAAGTCTGCTTTCATGGAGTTTGGACAGCAGTCGCACTCA

CAGCAGAGCTCCCCATCGATGGGCAGCGGCCACTACCCGCTGCACTGTCTTCACTCCGGC

TCACACTCTCACCACCAGCACGACAACACCCCGTACCCCGGGAGCAACACCTACAACAGG

TCGTTACCTTACCCTTACGTGAGCCATCCACACCACAGCCCGTACCTGCCGTCCTATCAC

AACAACATGGGAGGACAGACAAGGTTAGACGGCACAGAGCAGCAGAAGACGACAGTGATC

GAAAACGGGGAGATTCGTTTTAACGGCAAAGGCAAGAAGATTCGCAAACCTCGGACAATT

TATTCCAGTTTGCAGCTTCAAGCACTGAACCACCGTTTCCAGCAAACACAGTACCTCGCT

TTACCGGAGCGCGCCGAGCTGGCCGCCTCTCTAGGACTGACGCAAACACAGgTAAAAATT

TGGTTTCAGAATAAAAGGTCAAAGTTCAAGAAGCTGCTGAAGCAAGGCGGTAACCCGCAC

GAGAGCGAGCCCATCCCGGGCTCCATGTCCCTGTCCCCGCGCTCGCCGAGCATCCCCCCA

ATCTGGGACGTGTCGGCCTCATCCAAAGGAGTAAACATGCCGGCCAACAGCTACATGCCC

GGCTACTCTCACTGGTATTCCTCCCCACATCAAGACTCAATGCAGAGA

>dlx6a_Gnaper

CTGGAGGCTCAGGACTCGTCCAAGTCTGCTTTCATGGAGTTTGGACAGCAGTCGCACTCA

CAGCAGAGCTCCCCATCGATGGGCAGCGGCCACTACCCGCTGCACTGTCTCCACTCCGGC

TCACACTCTCACCACCAGCACGACAACACCCCGTACCCCGGGAGCAACACCTACAACAGG

TCGTTACCTTACCCTTACGTGAGCCATCCACACCACAGCCCGTACCTGCCGTCCTATCAC

AACAACATGGGAGGACAGACAAGGTTAGACGGCACAGAGCAGCAGAAGACGACAGTGATC

GAAAACGGGGAGATTCGTTTTAACGGCAAAGGCAAGAAGATTCGCAAACCTCGGACAATT

TATTCCAGTTTGCAGCTTCAAGCACTGAACCACCGTTTCCAGCAAACACAGTACCTCGCT

TTACCGGAGCGCGCCGAGCTGGCCGCCTCTCTAGGACTGACGCAAACACAGgTAAAAATT

TGGTTTCAGAATAAAAGGTCAAAGTTCAAGAAGCTGCTGAAGCAAGGCGGTAACCCGCAC

GAGAGCGAGCCCATCCCGGGCTCCATGTCCCTGTCCCCGCGCTCGCCGAGCATCCCCCCA

ATCTGGGACGTGTCGGCCTCATCCAAAGGAGTAAACATGCCGGCCAACAGCTACATGCCC

GGCTACTCTCACTGGTATTCCTCCCCACATCAA---------------

>dlx6a_Oretan

CTGGAGGCTCAGGACTCGTCCAAGTCTGCTTTCATGGAGTTTGGACAGCAGTCGCACTCA

CAGCAGAGCTCCCCATCGATGGGCAGCGGCCACTACCCGCTGCACTGTCTCCACTCCGGC

TCACACTCTCACCACCAGCACGACAACACCCCGTACCCCGGGAGCAACACCTACAACAGG

TCGTTACCTTACCCTTACGTGAGCCATCCACACCACAGCCCGTACCTGCCGTCCTATCAC

AACAACATGGGAGGACAGACAAGGTTAGACGGCACAGAGCAGCAGAAGACGACAGTGATC

GAAAACGGGGAGATTCGTTTTAACGGCAAAGGCAAGAAGATTCGCAAACCTCGGACAATT

TATTCCAGTTTGCAGCTTCAAGCACTGAATCACCGTTTCCAGCAAACACAGTACCTCGCT

TTACCGGAGCGCGCCGAGCTGGCCGCCTCTCTAGGACTGACGCAAACACAGgTAAAAATT

TGGTTTCAGAATAAAAGGTCAAAGTTCAAGAAGCTGCTGAAGCAAGGCAGTAACCCGCAT

GAGAGCGAGCCCATCCCGGGCTCCATGTCCCTGTCCCCACGCTCGCCGAGCATCCCCCCA

ATCTGGGACGTGTCGGCCTCATCCAAAGGAGTAAACATGCCGGCCAACAGCTACATGCCC

GGCTACTCTCACTGGTATTCCTCCCCACATCAAGACTCAATGCAGAGA

>dlx6a_Petfam

CTGGAGGCTCAGGACTCGTCCAAGTCTGCTTTCATGGAGTTTGGACAGCAGTCGCACTCA

CAGCAGAGCTCCCCATCGATGGGCAGCGGCCACTACCCGCTGCACTGTCTCCACTCCGGC

TCACACTCTCACCACCAGCACGACAACACTCCGTACCCCGGGAGCAACACCTACAACAGG

TCGTTACCTTACCCTTACGTGAGCCATCCACACCACAGCCCGTACCTGCCGTCCTATCAC

AACAACATGGGAGGACAGACAAGGTTAGACGGCACAG---------------CAGTGATC

GAAAACGGGGAGATTCGTTTTAACGGCAAAGGCAAGAAGATTCGCAAACCTCGGACAATT

TATTCCAGTTTGCAGCTTCAAGCACTGAACCACCGTTTCCAGCAAACACAGTACCTCGCT

TTACCGGAGCGCGCCGAGCTGGCCGCCTCTCTAGGACTGACGCAAACACAGgTAAAAATT

TGGTTTCAGAATAAAAGGTCAAAGTTCAAGAAGCTGCTGAAGCAAGGCGGTAACCCGCAC

GAGAGCGAGCCTATCCCGGGCTCCATGTCCCTGTCCCCGCGCTCGCCGAGCATCCCCCCA

ATCTGGGACGTGTCGGCCTCATCCAAAGGAGTAAACATGCCGGCCAACAGCTACATGCCT

GGCTACTCTCACTGGTATTCC---------------------------

>dlx6a_Psecur

CTGGAGGCTCAGGACTCGTCCAAGTCTGCTTTCATGGAGTTTGGACAGCAGTCGCACTCA

CAGCAGAGCTCCCCATCGATGGGCAGCGGCCACTACCCGCTGCACTGTCTCCACTCCGGC

TCACACTCTCACCACCAGCACGACAACACTCCGTACCCCGGGAGCAACACCTACAACAGG

TCGTTACCTTACCCTTACGTGAGCCATCCACACCACAGCCCGTACCTGCCGTCCTATCAC

AACAACATGGGAGGACAGACAAGGTTAGACGGCACAG------------CGACAGTGATC

GAAAACGGGGAGATTCGTTTTAACGGCAAAGGCAAGAAGATTCGCAAACCTCGGACAATT

TATTCCAGTTTGCAGCTTCAAGCACTGAACCACCGTTTCCAGCAAACACAGTACCTCGCT

TTACCGGAGCGCGCCGAGCTGGCCGCCTCTCTAGGACTGACGCAAACACAGgTAAAAATT

TGGTTTCAGAATAAAAGGTCAAAGTTCAAGAAGCTGCTGAAGCAAGGCGGTAACCCGCAC

GAGAGCGAGCCTATCCCAGGCTCCATGTCCCTGTCCCCGCGCTCGCCGAGCATCCCCCCA

ATCTGGGACGTGTCGGCCTCATCCAAAGGAGTAAACATGCCGGCCAACAGCTACATGCCT

GGCTACTCTCACTGGTATTCCTCCCCACATCAAGACTCAATGCAGAGA
