# Supplementary figures and images for: Sequence analyses of the distal-less homeobox gene family in East African cichlid fishes reveal signatures of positive selection
Source: BMC Evol Biol. 2013 Jul 17;13:153. doi: 10.1186/1471-2148-13-153 (PMC3728225; doi:10.1186/1471-2148-13-153)

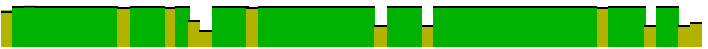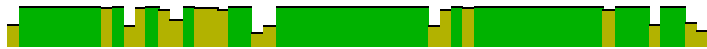[illegible]

Supplement: Additional file 5 — Maximum likelihood gene trees based on 23 cichlid species for the eight dlx loci. Bootstrap values (PAUP*) and Bayesian posterior probabilities (MrBayes) above 50% are shown respectively above and below the branches. A color key for the ten studied cichlid lineages is given in the box below the figure. (a) Dlx1a (737 base pairs (bp); TPM3uf model). Two major polytomies were recovered. The lamprologines cluster together with the Boulengerochromini, Bathybatini and the Cyphotilapiini. A. burtoni is found at the base with O. tanganicae. (b) Dlx2a (1371 bp; HKY + I model). Polytomous tree with all members of the lineages Lamprologines, Ectodines, Haplochromines and Limnochromines recovered as monophyletic clades. (c) Dlx3a (666 bp; HKY model). Polytomous tree, with only the Lamprologines recovered as monophyletic clade. (d) Dlx4a (1166 bp; TPM3uf + I + G). Polytomous relationships were observed between multiple lineages, although most lineages are monophyletic except the Haplochromines (e) Dlx3b (1972 bp; GTR + I + G). Moderately resolved tree. (f) Dlx4b (722 bp; TPM3uf). Mostly polytomous relationships between species, except the Limnochromini and most members of the Lamprologines. (g) Dlx5a (1538 bp; TIM2 + G). Basal polytomy divides ingroup species except G. permaxillaris, in two big clades. (h) Dlx6a (1710 bp; TIM3 + G). Limnochromines, Lamprologines and Haplochromines recovered as monophyletic clades, although the relationships between lineages are largely polytomous. [file 1471-2148-13-153-S5.pdf]

**A***Dlx1a*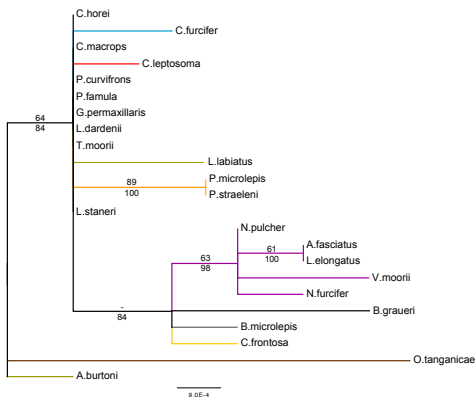**B***Dlx2a*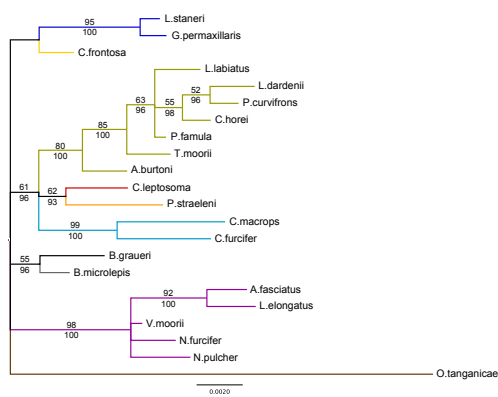**C***Dlx3a*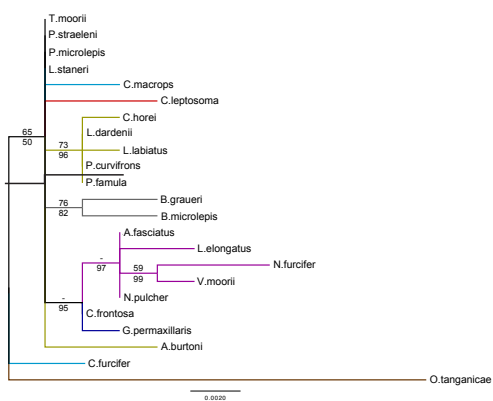**D***Dlx4a*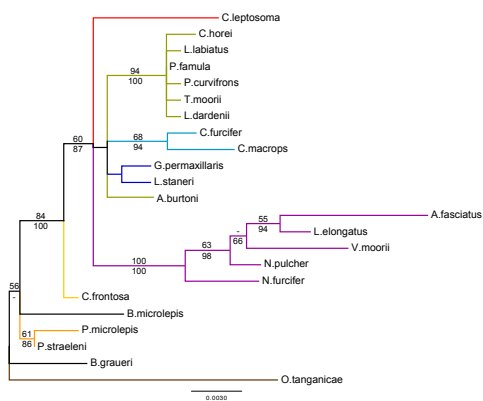**E***Dlx3b*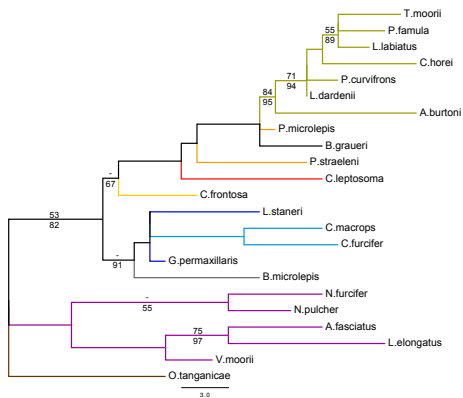**F***Dlx4b*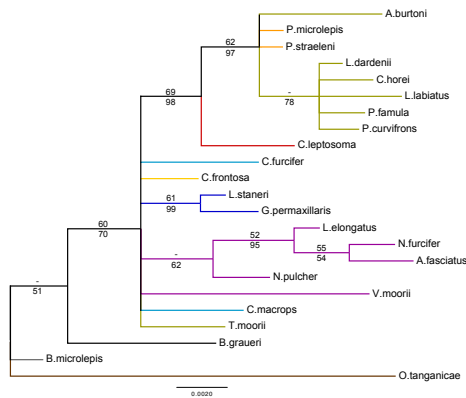**G***Dlx5a*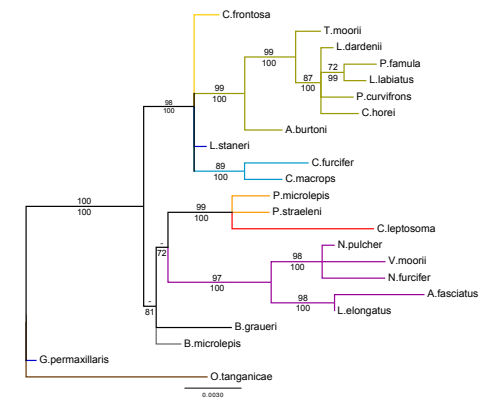**H***Dlx6a*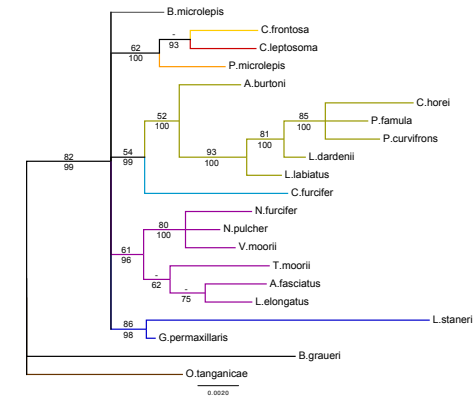

Supplement: Additional file 6 — Four partially sequenced cichlid Dlx proteins. Depicted are the amino acid sequences of Astatotilapia burtoni (a, c, d) and Ctenochromis horei (b). Secondary structure predictions were obtained from the PSIPRED server (http://bioinf.cs.ucl.ac.uk/psipred/). (a) Dlx3b. (b) Dlx4a. (c) Dlx5a. (d) Dlx6a. [file 1471-2148-13-153-S6.pdf]
